# Self-stacked small molecules for ultrasensitive, substrate-free Raman imaging in vivo

**DOI:** 10.1038/s41587-024-02342-9

**Published:** 2024-08-21

**Authors:** Shuai Gao, Yongming Zhang, Kai Cui, Sihang Zhang, Yuanyuan Qiu, Yunhui Liao, Haoze Wang, Sheng Yu, Liyang Ma, Hongzhuan Chen, Minbiao Ji, Xiaohong Fang, Wei Lu, Zeyu Xiao

**Affiliations:** 1 School of Pharmacy & Minhang Hospital, State Key Laboratory of Molecular Engineering of Polymers, Key Laboratory of Smart Drug Delivery Ministry of Education, Fudan University, Shanghai, China; 2 Department of Pharmacology and Chemical Biology, Institute of Molecular Medicine, Shanghai Key Laboratory for Nucleic Acid Chemistry and Nanomedicine, Key Laboratory of Cell Differentiation and Apoptosis of Chinese Ministry of Education, Shanghai Jiao Tong University School of Medicine, Shanghai, China; 3 Hangzhou Institute of Medicine (HIM), Chinese Academy of Sciences, Hangzhou, Zhejiang China; 4 State Key Laboratory of Surface Physics and Department of Physics, Key Laboratory of Micro and Nano Photonic Structures (Ministry of Education), Shanghai Key Laboratory of Metasurfaces for Light Manipulation, Fudan University, Shanghai, China; 5 Shuguang Lab for Future Health, Shuguang Hospital, Shanghai University of Traditional Chinese Medicine, Shanghai, China

**Keywords:** Imaging techniques and agents, Raman spectroscopy, Optical imaging, Raman spectroscopy

## Abstract

Raman spectroscopy using surface-enhanced Raman scattering (SERS) nanoprobes represents an ultrasensitive and high-precision technique for in vivo imaging. Clinical translation of SERS nanoprobes has been hampered by biosafety concerns about the metal substrates used to enhance Raman signals. We report a set of small molecules with bis-thienyl-substituted benzobisthiadiazole structures that enhance Raman signal through self-stacking rather than external substrates. In our technique, called stacking-induced charge transfer-enhanced Raman scattering (SICTERS), the self-stacked small molecules form an ordered spatial arrangement that enables three-dimensional charge transfer between neighboring molecules. The Raman scattering cross-section of SICTERS nanoprobes is 1350 times higher than that of conventional SERS gold nanoprobes of similar particle size. SICTERS outperforms SERS in terms of in vivo imaging sensitivity, resolution and depth. SICTERS is capable of noninvasive Raman imaging of blood and lymphatic vasculatures, which has not been achieved by SERS. SICTERS represents an alternative technique to enhance Raman scattering for guiding the design of ultrasensitive substrate-free Raman imaging probes.

## Abstract (web summary)

The sensitivity of in vivo Raman imaging is improved with self-stacked small molecules.

## Main

Raman spectroscopy has been widely applied in chemical, material and biomedical sciences by detecting the inelastic light scattering of small molecules^1^. Complementary to fluorescence or other imaging modalities, Raman spectroscopy for bioimaging measures chemical-bond-specific vibrational transitions that unravels chemical information of molecules such as nucleic acids, proteins and lipids, related to the genotype, phenotype and physiological state of the cell^2^. The ultranarrow spectral peak width of Raman signal offers the opportunity to detect multiple tags simultaneously using a single laser excitation, capable of multiplexed imaging^3^. Moreover, owing to the extremely short lifetimes of Raman scattering, Raman signals are stable against photobleaching or quenching, which is ideal for imaging studies of prolonged duration^4^.

Despite these advantageous properties, Raman scattering of small molecules is an extremely inefficient process, with cross-sections generally ranging from 10^−28^ to 10^−30^ cm^2^ per molecule^4,5^. To enhance signal intensity, one major strategy is to rely on the surface-enhanced Raman scattering (SERS) effect^4,6^. By adsorbing small-molecule Raman reporters on the surface of inorganic (for example, Au, Ag or Cu) or organic (for example, semiconductor films) substrates, SERS can amplify the Raman signals up to 10^8^–10^11^ times^7–9^, enabling ultrasensitive imaging with the detection sensitivity in the range of μM to pM^7^. Nevertheless, due to the biosafety concerns of these substrate materials, the use of substrates has limited the breadth of practical applications of SERS^2,10,11^. As such, a long-existing challenge in high-resolution Raman imaging in vivo is to explore small molecules as reporters for enhanced Raman scattering without relying on substrates.

Herein, we report substrate-free Raman scattering enhancement of small molecules for highly sensitive imaging, through a stacking-induced charge-transfer-enhanced Raman scattering (SICTERS) mechanism. SICTERS requires that small molecules possess a π-conjugated and planarized conformation with in-plane multi-ring vibrations, and are capable of self-stacking to form an ordered spatial arrangement with close intermolecular distances, allowing for a three-dimensional charge transfer among the neighboring molecules. Comparative experiments demonstrate that the SICTERS-based small-molecule nanoprobes outperform the SERS-based Au nanoprobes of similar size in terms of the Raman scattering cross-sections, as well as have the capability for intraoperative detection of microtumors and noninvasive imaging of blood and lymphatic vasculatures.

## Results

### Raman enhancement of self-stacked small molecules

**Fig. 1:**
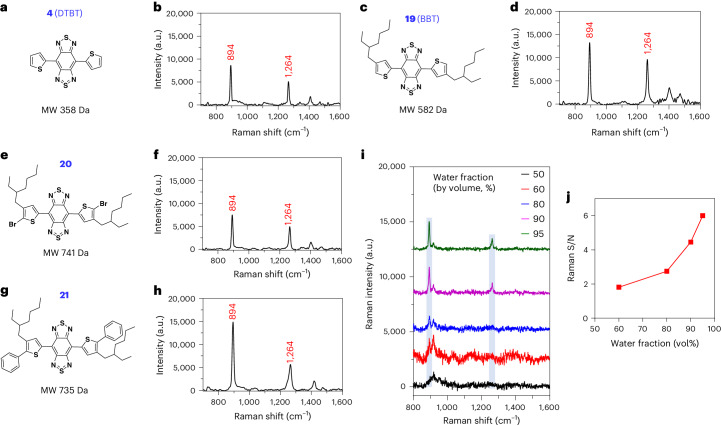
Raman spectra of the DTBT-based small molecules with enhanced Raman scattering effect through aggregation. **a**–**h**, Chemical structure and Raman spectrum of DTBT (**a** and **b**), BBT (**c** and **d**), compounds **20** (**e** and **f**) and **21** (**g** and **h**) measured in solid state, respectively. **i**, Raman spectra of BBT (10 µM) in water/THF mixture. **j**, Plot of S/N of Raman intensity at 894 cm^−1^ versus the water fraction of the THF/water mixtures in panel **i**. When the water fraction was 50%, BBT did not show signal peak at 894 cm^−1^. Raman measurement was carried out with an 830-nm laser excitation, a ×20 objective, a laser power of 6.1 × 10^−2^ mW, acquisition time of 5 s and 3 times accumulation in (**b**, **d**, **f** and **h**); a ×5 objective, a laser power of 62.6 mW, acquisition time of 1 s and 1 time accumulation in panel **i**. Blue columns in panel **i** represent the featured Raman peaks at 894 and 1,264 cm^−1^. MW, molecular weight.

The small molecules are represented by a class of π-conjugated molecules with the structure of bis-thienyl-substituted benzobisthiadiazole, such as 4,7-di(thiophen-2-yl)benzobis[*c*][1,2,5]thiadiazole (DTBT; **4**, molecular weight 358 Da) and its derivative with two alkyl chains, 4,7-bis(4-(2-ethylhexyl)thiophen-2-yl)benzobis[*c*][1,2,5]thiadiazole (BBT; **19**, molecular weight 582 Da) (Fig. 1a–d). Illumination of BBT in solid state with either 785-nm or 830-nm near-infrared (NIR) laser, but not 532-nm laser, led to resonance Raman scattering with featured and intense Raman spectral signatures at 894 and 1264 cm^−1^ (Supplementary Fig. 1). The assignment of these two characteristic peaks was investigated by comparing BBT or DTBT with their building blocks (Supplementary Figs. 2–12). The two spectral signatures were not observed after deleting the two thiophenes of DTBT (**7**, Supplementary Fig. 13a). Thiophene alone or phenyl thiophene(s) (**11** and **12**) failed to show these characteristic Raman peaks (Supplementary Fig. 13b–d). In the structure of DTBT, the replacement of central benzobisthiadiazole with dinitro-substituted benzothiadiazole (**14**) or the two thiophene units with phenyls (**5**) resulted in the disappearance of the two spectral signatures (Supplementary Fig. 13e, f). BBT coupled with bromo or phenyl groups on the α-position of the thiophene moieties (**20** and **21**), however, possessed the two signature peaks (Fig. 1e–h). These data demonstrated that the peaks of 894 and 1,264 cm^−1^ are attributed to the vibration mode from the entire backbone (that is, DTBT).

**Extended Data Fig. 1:**
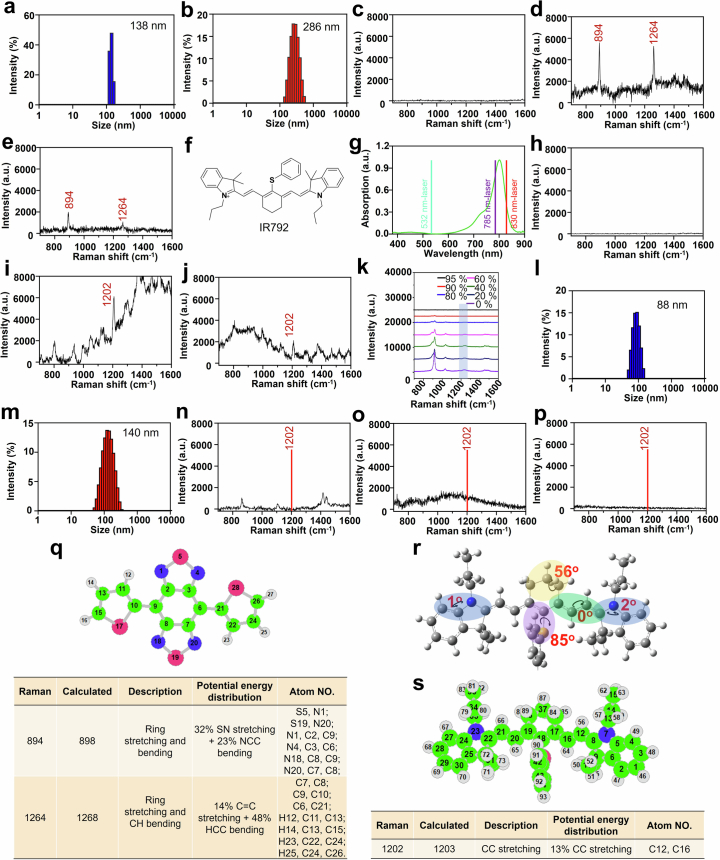
Comparison of Raman signals between BBT and IR792 in the water/THF mixture as well as the calculated vibration modes between DTBT and IR792. **a**,**b**, Size distributions of BBT in the mixture with a water fraction of 60% (**a**) and 95% (**b**) analyzed by dynamic light scattering, respectively. **c**-**e**, Raman spectra of BBT (10 μM) measured in the mixture of H_2_O and THF (95%: 5%) with 532-nm (**c**), 785-nm (**d**) and 830-nm (**e**) laser excitation, respectively. Raman measurement was carried out with a 5 × objective, acquisition time of 1 s, one time accumulation, and laser powers of 15.8 mW for 532-nm laser, 84.5 mW for 785-nm laser or 62.6 mW for 830-nm laser. **f**, The chemical structure of IR792. **g**, UV-Vis absorption spectrum of IR792 (10 μM) in THF. **h**-**j**, Raman spectra of IR792 measured in solid state with 532-nm (**h**), 785-nm (**i**) and 830-nm (**j**) laser excitation, respectively. Raman measurement was carried out with a 20 × objective, acquisition time of 10 s, one time accumulation, and laser powers of 8.2 × 10^−2^ mW for 532-nm laser, 8.2 × 10^−2^ mW for 785-nm laser or 6.1 × 10^−2^ mW for 830-nm laser. **k**, Raman spectra of IR792 (10 µM) without showing the characteristic peak at 1202 cm^−1^ (blue column) in the water/THF mixture (water fraction 0%–95%) excited at 830 nm. **l**,**m**, Size distributions of IR792 in the water/THF mixture with a water fraction of 60% (**l**) and 95% (**m**) analyzed by dynamic light scattering, respectively. **n**-**p**, Raman spectra of IR792 (10 μM) measured in the mixture of H_2_O and THF (95%: 5%) with 532-nm (**n**), 785-nm (**o**) and 830-nm (**p**) laser excitation, respectively. Raman measurement was carried out with a 5× objective, acquisition time of 10 s, one time accumulation, and laser powers of 7.9 × 10^−1^ mW for 532-nm laser, 8.45 × 10^−5^ mW for 785-nm laser or 6.26 × 10^−5^ mW for 830-nm laser. **q**, Experimental and calculated vibrational frequencies (cm^−1^) of DTBT and the corresponding assignments by DFT (Gaussian 09/B3LYP/6-31 G(d)). **r**, The optimized geometry of IR792 with the dihedral angles calculated by DFT (Gaussian 09/B3LYP/6-31 G(d)). Color circles represent dihedral angles. **s**, Experimental and calculated vibrational frequencies (cm^−1^) of IR792 and the corresponding assignments by DFT (Gaussian 09/B3LYP/6-31 G(d)).

The poor solubility of DTBT makes it difficult to perform spectroscopy in solution. Alternatively, the lipophilic BBT exhibited enhanced Raman scattering in water through its self-stacking. In the water and tetrahydrofuran (THF) mixture (50%:50%), BBT is soluble without showing the Raman signature peak (Fig. 1i, black curve). When the water fraction was increased to 60%, BBT started to aggregate with the appearance of the Raman peak at 894 cm^−1^ (Fig. 1i, red curve). The Raman signal-to-noise (S/N) ratio of BBT was continuously elevated when the water fraction was further increased (Fig. 1i,j), correlated with the increased aggregation of the molecules (Extended Data Fig. 1a–e). In comparison, a conventional Raman reporter molecule, the hydrophobic IR792, with the absorption spectrum similar to that of BBT, did not show any Raman signal intensities in water (Extended Data Fig. 1f–p).

**Fig. 2:**
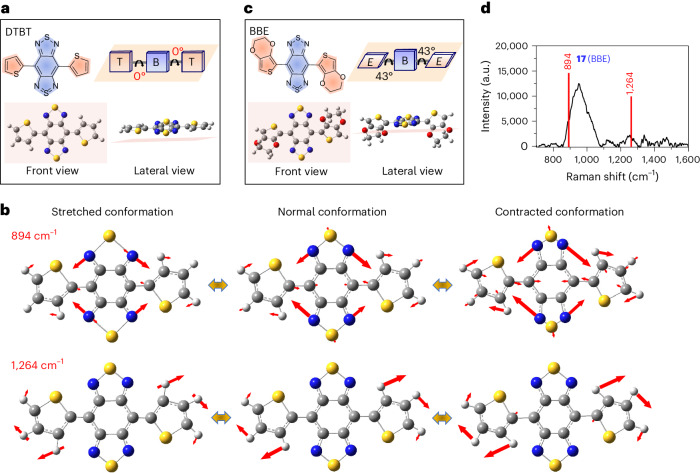
In-plane multiring vibration modes of the planar molecule DTBT. **a**, The optimized geometry of DTBT with the dihedral angles calculated by DFT (Gaussian 09/B3LYP/6-31 G(d)). B, benzobisthiadiazole unit. T, thiophene unit. **b**, Illustration of in-plane multiring vibration modes of DTBT. The peak at 894 cm^−1^ represents ring stretching and bending modes of the multiring skeleton (Supplementary Videos 1 and 2). The peak at 1,264 cm^−1^ represents ring stretching and C-H bending modes of the multiring skeleton (Supplementary Videos 3 and 4). Red arrows, the vibration amplitude and direction of atoms. **c**, The optimized geometry of BBE with the dihedral angles calculated by DFT (Gaussian 09/B3LYP/6-31 G(d)). B, benzobisthiadiazole unit; E, 3,4-ethoxylene dioxythiophene unit. **d**, Raman spectrum of BBE measured in solid state, with an absence of Raman peaks at 894 or 1,264 cm^−1^. Raman measurement was carried out with an 830-nm laser excitation, a ×20 objective, a laser power of 6.1 × 10^−2^ mW, 3 times accumulation and an acquisition time of 0.2 s.

Density functional theoretical (DFT) calculation revealed that the optimized geometry of DTBT had a planar conformation with 0° of dihedral angles between the benzobisthiadiazole core and the thiophene blocks (Fig. 2a). The two characteristic Raman peaks at 894 and 1,264 cm^−1^ were assigned to the stretching and bending of the in-plane vibration mode, derived from the whole multiring structure of DTBT (Fig. 2b and Supplementary Video 1–4). Specifically, the Raman peak at 894 cm^−1^ was assigned to the ring stretching and bending including the SN stretching and NCC bending, whereas the Raman peak at 1,264 cm^−1^ represented the ring stretching and CH bending including the C = C stretching and HCC bending (Extended Data Fig. 1q). By contrast, IR792 owned a twisted conformation, with the featured peak (1,202 cm^−1^) mainly assigned to the C-C stretching (Extended Data Fig. 1r,s and Supplementary Video 5). The comparative results indicated that the planar conformation with the in-plane multiring vibration of DTBT plays a pivotal role in the enhanced Raman scattering. To validate this point, the two thiophenes of DTBT were replaced with 3,4-ethoxylene dioxythiophenes, to obtain a molecule BBE (**17**) with 43° of dihedral angles between the benzobisthiadiazole and 3,4-ethoxylene dioxythiophenes (Fig. 2c). The enlarged dihedral angles of BBE broke the planar conformation and in-plane multiring vibration, resulting in a complete loss of these characteristic Raman peaks (Fig. 2d).

### Raman enhancement mechanism

**Fig. 3:**
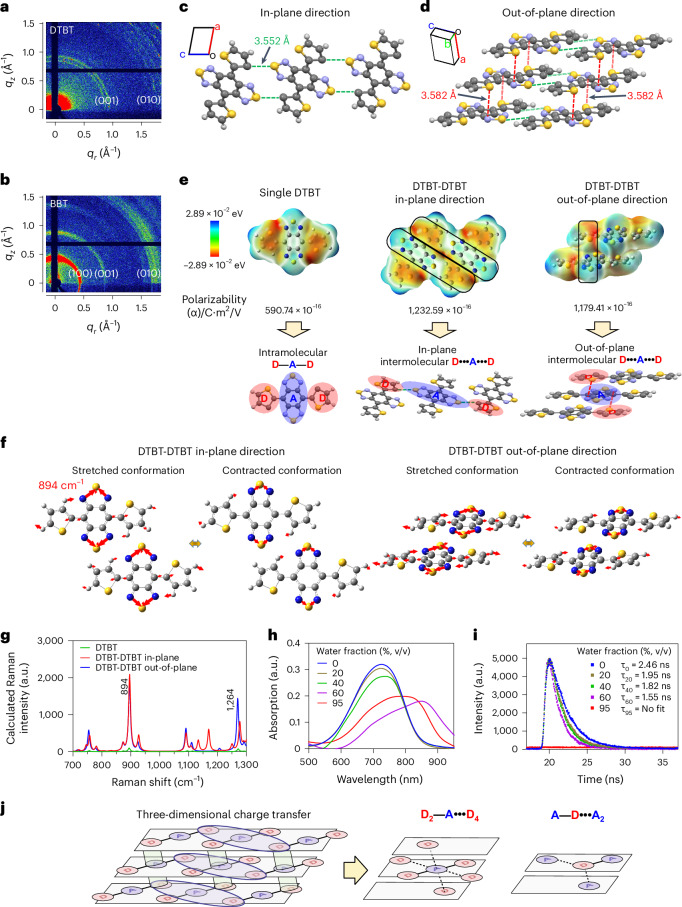
Stacking-induced intermolecular charge transfer of the DTBT-based planar D-A-D molecules. **a**,**b**, 2D-GIWAXS pattern of the thin films of DTBT (**a**) and BBT (**b**), respectively. *q*_*z*_ and *q*_*r*_, the out-of-plane and in-plane directions, respectively. **c**,**d**, BFDH theoretical crystal morphology of DTBT simulated by Mercury 3.8 based on the single-crystal structure from the Cambridge Crystallographic Data Centre (CCDC; code: 1308377). The arrangement of DTBT in the in-plane direction (**c**) and out-of-plane direction (**d**). **e**–**g**, ESP maps (**e**), vibration mode (894 cm^−1^) (**f**) and Raman spectra (**g**) of single DTBT or DTBT-DTBT packed in the in-plane or out-of-plane direction calculated by Gaussian 09/B3LYP/6-31 G(d) based on their crystal geometries from the CCDC. Color bar indicates the ESP energy value between the highest (blue) and the lowest (red) values (**e**). Boxed area in the ESP maps, intermolecular charge-transfer. **h**,**i**, Absorption spectra (**h**) or fluorescence decay curves (**i**) of BBT in water/THF mixtures with water fraction from 0% to 95%. No fit, the fluorescence emission was completely quenched. **j**, Schematic diagram of three-dimensional charge-transfer interactions of DTBT, substantially increasing the extent to electron delocalization to conduce to the enhanced Raman scattering. Green cylinders, intermolecular D•••A change-transfer in the out-of-plane direction. Blue ellipses, intermolecular D•••A change-transfer in the in-plane direction.

**Extended Data Fig. 2:**
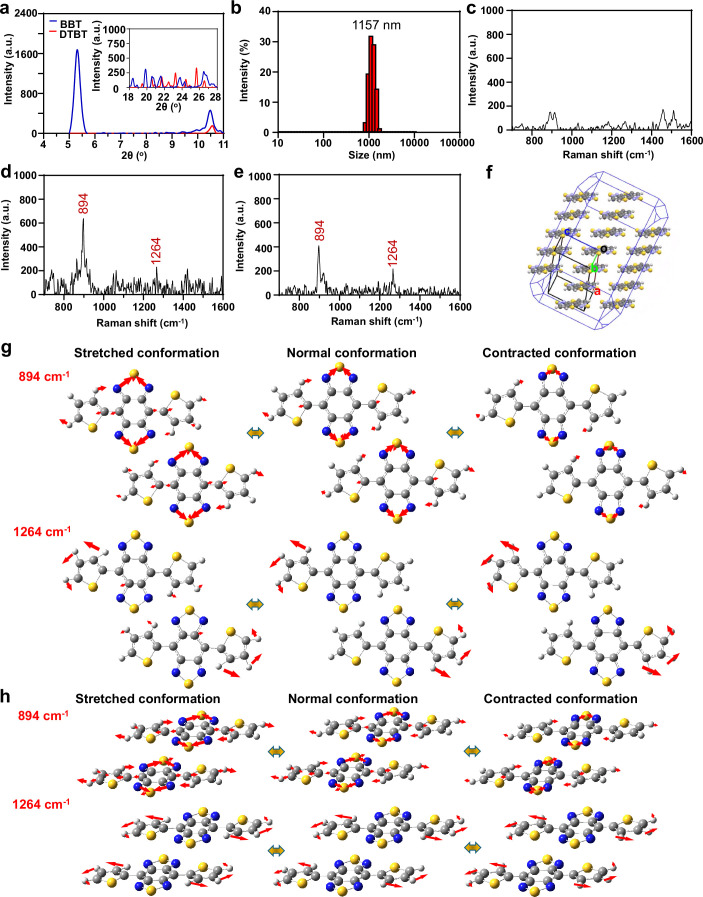
Characterization of the stacked DTBT or BBT and enhanced multi-ring vibration mode following the DTBT-DTBT stacking. **a**, XRD analysis of BBT or DTBT powder on the SiO_2_/Si substrate. The diffraction peak of 10.5° demonstrated an in-plane *d*-spacing distance of 8.5 Å of the two adjacent BBT backbones. The π-π stacking between the BBT backbones in the direction of π-orbitals of the conjugated rings, with the diffraction peaks at 18.4°, 19.6°, 20.6°, 21.6°, 23.7° and 26.5° corresponding to the *d*-spacing distances of 4.8 Å, 4.5 Å, 4.3 Å, 4.2 Å, 3.7 Å and 3.3 Å, respectively (inset, blue curves). The lamellar stacking separated the multi-ring backbones by side chains in the direction perpendicular to the π-π stacking, with the diffraction peak at around 5.3° (*d*-spacing distance = 16.6 Å). Comparatively, DTBT displayed the backbone-to-backbone repeat construction and π-π stacking, verified by the diffraction peak of 10.5° (*d*-spacing distances = 8.6 Å) and peaks between 20.6° and 25.6° (*d*-spacing distances = 4.3-3.5 Å), but lack of the lamellar stacking (red curves). **b**, Size distribution of DTBT in the water/THF mixture with a water fraction of 95% analyzed by dynamic light scattering. **c**-**e**, Raman spectra of DTBT (10 μM) measured in the mixture of H_2_O and THF (95%: 5%) with 532-nm (**c**), 785-nm (**d**) and 830-nm (**e**) laser excitation, respectively. Raman measurement was carried out with a 5 × objective, acquisition time of 1 s, one time accumulation, and laser powers of 15.8 mW for 532-nm laser, 84.5 mW for 785-nm laser or 62.6 mW for 830-nm laser. **f**, BFDH theoretical crystal morphology of DTBT simulated by Mercury 3.8 based on the single-crystal structure from the Cambridge Crystallographic Data Centre (CCDC, code: 1308377). The whole view of the packing pattern in the crystal. **g**, The amplitude and direction of atomic vibration of the two DTBT molecules packed in the in-plane direction at 894 cm^−1^ and 1264 cm^−1^ by Gaussian 09/B3LYP/6-31 G(d), respectively (Supplementary Videos 7 and 9). **h**, The amplitude and direction of atomic vibration of the two DTBT molecules packed in the out-of-plane direction at 894 cm^−1^ and 1264 cm^−1^ by Gaussian 09/B3LYP/6-31 G(d), respectively (Supplementary Videos 8 and 10). Red arrows, the amplitude and direction of the atomic vibration.

To understand how the DTBT-based planar molecules generate the enhanced Raman intensity through self-stacking, we analyzed the intermolecular stacking information by two-dimensional grazing-incidence wide-angle X-ray scattering (2D-GIWAXS) measurement. DTBT showed a backbone-to-backbone repeat distance in the molecular plane (in-plane) assigned to (001) peak at the *q* value around 0.8 Å^−1^, and a π-π stacking between the backbones in the direction of π-orbitals of the conjugated rings (out-of-plane) characterized by the (010) peak at the *q* value between 1.5 and 1.8 Å^−1^ in its diffraction pattern (Fig. 3a)^12^. Besides the existence of (001) and (010) peaks, BBT displayed a lamellar stacking (100) peak due to its alkyl side chains (Fig. 3b)^13^. Results of the X-ray diffraction (XRD) test further supported the stacking of both molecules (Extended Data Fig. 2a). Considering DTBT also exhibited the featured Raman signals upon its aggregation (Extended Data Fig. 2b–e), we concluded that the in-plane backbone-to-backbone and the out-of-plane π-π stacking rather than the lamellar stacking contribute to the intense Raman scattering of the DTBT-based molecules.

The stacking of DTBT was visually presented via the BFDH (Bravais, Friedel, Donnay and Harker) theoretical crystal morphology simulated based on the single-crystal structure from the Cambridge Crystallographic Data Centre (CCDC; code 1308377) (Extended Data Fig. 2f and Supplementary Video 6)^14,15^. The crystal formed a backbone-to-backbone parameter in the in-plane direction, with a 3.552 Å of gap between the carbon atom of the thiophene in one DTBT molecule and the sulfur atom of the benzobisthiadiazole in the neighboring DTBT molecule (Fig. 3c, green dotted lines). Meanwhile, the molecules were piled up in the out-of-plane direction to constitute a continuous π-π stacking, with a 3.582 Å of distance between the sulfur atom of the thiophene in the upper molecular plane and the nitrogen atom of the benzobisthiadiazole in the lower plane (Fig. 3d, red dotted lines).

According to electromagnetic theory, the intensity of Raman scattering is proportional to the square of the electric dipole moment (*ρ*), where *ρ* is expressed as the product of polarizability of the molecule and electric field intensity^16–18^. Small molecules with a donor-acceptor-donor (D-A-D) scaffold constructed by the conjugation of both electron-donating and electron-withdrawing blocks possess a favorable intramolecular charge-transfer property, facilitating the corresponding molecular polarizability^19–23^. The results of maps of electrostatic potential (ESP) showed substantially increased intramolecular charge-transfer characteristic and polarizability of DTBT or BBT compared with those of benzobisthiadiazole due to the formation of a strong D-A-D system (Supplementary Fig. 14a). The highest occupied molecular orbitals and lowest unoccupied molecular orbitals of the four molecules also demonstrated that the conjugated D-A-D system increased the extent to electron delocalization and lowered the energy gap (E_g_), facilitating charge-transfer excitations (Fig. 3e, left, and Supplementary Fig. 14b).

More importantly, the maps of ESPs delineated strong intermolecular charge-transfer interactions between the thiophene unit of one DTBT molecule and the benzobisthiadiazole unit of the neighboring molecule stacked in the in-plane direction (Fig. 3e, middle, boxed area). In the out-of-plane direction, the thiophene unit in the upper molecular plane was piled up on the top of the benzobisthiadiazole unit in the lower plane, thus promoting the intermolecular charge-transfer interactions as well (Fig. 3e, right, boxed area). Consequently, dual DTBT molecules stacked in either the in-plane or out-of-plane direction doubled the molecular polarizability (Fig. 3e). The enhanced intermolecular charge-transfer interactions were attributed to the in-plane intermolecular gaps of 3.552 Å and the out-of-plane intermolecular gaps of 3.582 Å shown in the crystal of DTBT, respectively (Fig. 3c,d). An intermolecular D-A distance at such a close range fostered relatively strong intermolecular charge-transfer interactions^24^. Moreover, DFT calculation demonstrated that the vibrational amplitude as well as direction of atoms in the two DTBT molecules stacked in either direction were synchronized, amplifying the Raman intensity at 894 cm^−1^ by ~20-fold (Fig. 3f,g, blue and red peaks versus green peak, Extended Data Fig. 2g,h and Supplementary Videos 7–10).

In addition, we prepared the single crystal of BBT and characterized its molecular packing by the single-crystal XRD experiment (Supplementary Fig. 15a–c and Supplementary Table 1). The crystal structure clearly demonstrated a close intermolecular distance of 3.427 Å between the sulfur atom of the thiophene in one BBT molecule and the nitrogen atom of the benzobisthiadiazole in the neighboring BBT molecule through backbone-to-backbone stacking along the *c* axis (Supplementary Fig. 15d,e). The BBT molecules were piled up in the out-of-plane direction to constitute a continuous π-π stacking, with a close distance of 3.618 Å between the sulfur atom of the thiophene in one molecular plane and the nitrogen atom of the benzobisthiadiazole in the other molecular plane (Supplementary Fig. 15f).

The absorption spectra of BBT in THF with different ratios of water were further analyzed. When the water fraction was increased to 60% or 95% in the THF:water mixture (the aggregated state), the absorption spectra of BBT exhibited a red-shifted and broader peak, indicating the existence of exciton coupling, substantiating the intermolecular charge-transfer interactions (Fig. 3h)^25^. Moreover, an increase in the aggregation of BBT reduced its fluorescent lifetime, suggesting the presence of the intermolecular charge transfer (Fig. 3i)^26^.

Taken together, the SICTERS effect requires the small molecule (for example, DTBT or BBT) possessing a D-A-D-based planar conformation and an in-plane multiring vibration mode. The molecular stacking of DTBT or BBT facilitates the intermolecular charge-transfer in both in-plane or *c* axis and out-of-plane directions. As a result, one acceptor could totally receive electrons from six donors (D_2_—A•••D_4_), including two intramolecular and four intermolecular donors. Meanwhile, one donor could donate electrons to three acceptors (A—D•••A_2_), including one intramolecular and two intermolecular acceptors (Fig. 3j and Supplementary Fig. 15g,h). These spatial arrangements readjust the charge distributions to form a three-dimensional charge transfer, substantially increasing the polarizability and the resonance Raman scattering.

### Preparation of SICTERS nanoprobes for in vivo applications

**Fig. 4:**
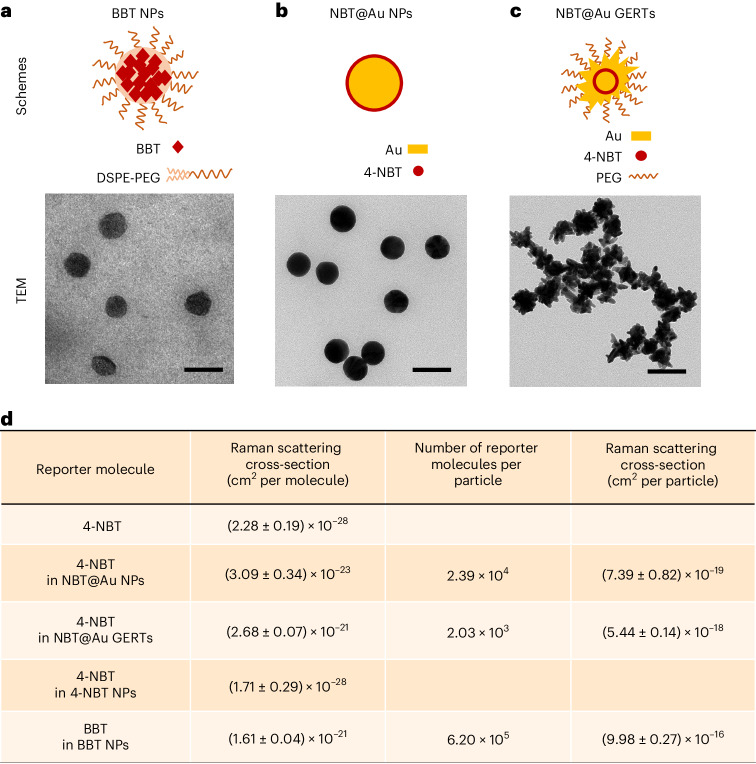
Raman scattering cross-section of the SICTERS-based small-molecule nanoprobes compared to the SERS-based Au nanoprobes. **a**–**c**, Schematic structures and representative transmission electron micrographs (TEM) of the SICTERS-based BBT NPs (**a**), the SERS-based Au NPs (NBT@Au NPs) (**b**) and the SERS-based Au GERTs (NBT@Au GERTs) (**c**) of 69.1 nm, 68.3 nm and 70.6 nm in average diameters, respectively. Scale bars, 100 nm. The reporter molecule 4-NBT was incorporated into Au NPs or Au GERTs. Image shown is representative of *n* = 3 independent replicates of experiments with similar results (**a**–**c**). **d**, The Raman scattering cross-section for 4-NBT or BBT in different nanoprobes. The cross-section values per molecule were calculated by measuring the Raman peak at 1,340 cm^−1^ excited at 532 nm for 4-NBT or 4-NBT NPs; the Raman peak at 1,340 cm^−1^ excited at 785 nm for NBT@Au NPs or NBT@Au GERTs; and the Raman peak at 894 cm^−1^ excited at 830 nm for BBT NPs, while using the methanol C-O stretch at 1,020 cm^−1^ as the internal reference. *n* = 3–5 independent samples **(d**). Data are presented as the mean ± standard deviation (s.d.) (**d**). Source data

**Extended Data Fig. 3:**
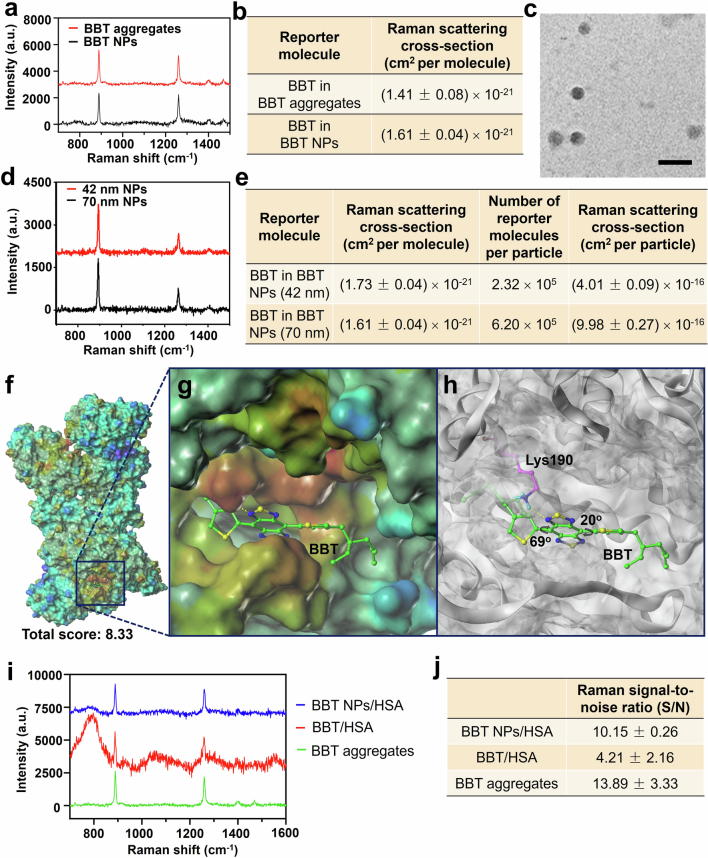
Raman signal intensities of BBT aggregates and BBT NPs. **a**, Raman spectra of BBT aggregates (10 μM) in water/THF mixture (95:5, v/v) and BBT NPs (10 μM of BBT) with a BBT/DSPE-PEG ratio of 5:1 (mol/mol) in water, respectively. **b**, The Raman scattering cross-sections for per molecule in (**a**). **c**, Transmission electron micrographs of 42 nm-BBT NPs. Scale bar, 100 nm. Image shown is representative of n = 3 independent replicates of experiments with similar results. **d**,**e**, Raman spectra of (**d**), and Raman scattering cross-sections (**e**) in BBT NPs (42 nm *v.s*. 70 nm in average diameters) with the same BBT/DSPE-PEG (5:1, mol/mol) ratio. Raman scattering cross-sections of BBT were calculated by measuring the Raman peak at 894 cm^−1^. **f**, Overview of the molecular docking of BBT (green) binding to HSA (PDB code: 6wuw). The orange indicates a high electrostatic energy. Total score, 8.33. **g**, Active pocket. **h**, Functional residue (Lys190, magenta) of HSA interacting with BBT (green). Yellow dashed line, hydrogen bond between HSA and BBT. Two twisted dihedral angles of BBT, 69° and 20°, respectively. **i**, Raman spectra of BBT (10 uM), the mixture of BBT and HSA (BBT/HSA, 1:50 mol:mol, 10 uM of BBT), and the mixture of BBT NPs and HSA (BBT NPS/HSA, 1:50 mol:mol, 10 uM of BBT), respectively. **j**, Raman signal-to-noise (S/N) ratio at 894 cm^−1^ in (**i**). Raman measurement was carried out with an 830-nm laser excitation, a 5 × objective, a laser power of 62.6 mW, acquisition time of 1 s, and one time accumulation. The cross-section values of BBT NPs were calculated by the Raman peak at 894 cm^−1^ excited at 830 nm, while using the methanol C-O stretch at 1,020 cm^−1^ as the internal reference (**b,e**). n = 3 independent samples (**b,e,j**). Data are presented as the mean ± s.d. (**b,e,j**). Source data

For in vivo bioimaging study, the SICTERS nanoprobes were prepared by encapsulating BBT with the amphiphilic 1,2-distearoyl-*sn*-glycero-3-phosphoethanolamine-*N*-[methoxy-(polyethylene glycol)-2000] (DSPE-PEG) to form core-shell BBT nanoparticles (NPs) (Fig. 4a). The Raman scattering cross-section of BBT per molecule in BBT NPs was close to that in the BBT aggregates in the water/THF (95: 5, v/v) mixture (Extended Data Fig. 3a,b). BBT NPs of different sizes in diameter (~42 nm versus ~70 nm) had roughly equivalent Raman scattering cross-sections per molecule (Extended Data Fig. 3c–e). These results indicated that the nanoassembly with DSPE-PEG and the particle size did not alter the intermolecular distance of BBT stacking.

The encapsulation with DSPE-PEG improved the stability of the NPs in serum. The BBT molecule can bind to one pocket of human serum albumin (HSA), leading to the twisted dihedral angles of 69° and 20° between the benzobisthiadiazole core and two thienyl groups (Extended Data Fig. 3f–h). In comparison to the BBT aggregates, the mixture of BBT and HSA substantially reduced the Raman S/N ratio at 894 cm^−1^ from 13.89 to 4.21, indicating that the binding interfered with the effect of SICTERS (Extended Data Fig. 3i,j). Comparatively, the Raman S/N ratio of BBT NPs mixed with HSA remained to be 10.15 (Extended Data Fig. 3j). These results demonstrated that the NP formulation with DSPE-PEG can prevent the encapsulated BBT molecule from binding to HSA, thus maintaining the Raman signal of BBT. BBT NPs were stable in 10% fetal bovine serum without significant change in size distribution or Raman signal intensity for 48 h at room temperature and for 24 h at 37 °C, respectively (Supplementary Fig. 16a–d). BBT NPs remained stable in the lysosomal lysate for 30 h (Supplementary Fig. 16e,f).

We next quantitatively compared the Raman enhancement of SICTERS with that of SERS by measuring their Raman scattering cross-section values. Two types of widely used SERS nanoprobes with a particle size similar to BBT NPs were formulated. One was the traditional type—Au NPs, with the conventional Raman reporter molecules (4-nitrobenzenethiol, 4-NBT) coated on the Au surface (NBT@Au NPs; Fig. 4b)^7,27^. The other was an advanced type considered to be among the most sensitive SERS nanoprobes for in vivo applications—Au gap-enhanced Raman tags (Au GERTs), with 4-NBT embedded in the electromagnetic hot spots from the interior nanogaps of the Au core-shell junction (NBT@Au GERTs; Fig. 4c)^9,28,29^. In both types, the Raman spectral signature of 4-NBT at 1,340 cm^−1^ was used for characterization^29^. The Raman scattering cross-section for 4-NBT in solution was measured ~10^−28^ cm^2^ per molecule (Fig. 4d and Supplementary Table 2). Through the SERS effect, Au NPs and Au GERTs enhanced the cross-sections of 4-NBT to ~10^−23^ and ~10^−21^ cm^2^ per molecule, respectively. 4-NBT NPs prepared with DSPE-PEG through self-assembly did not exhibit any Raman scattering enhancement. By contrast, the Raman scattering cross-section of BBT in substrate-free BBT NPs reached ~10^−21^ cm^2^ per molecule, which was two orders of magnitude larger than NBT@Au NPs and the same order of magnitude as NBT@Au GERTs, respectively (Fig. 4d). Different from the SERS nanoprobes like Au NPs or Au GERTs with the Au core as a substrate, the core of the SICTERS-based BBT NPs was only composed of BBT (the reporter molecules) free of substrate. Given the similar particle sizes of ~70 nm, the loading of reporter molecules in BBT NPs was 26-fold higher than that of NBT@Au NPs and 305-fold higher than that of NBT@Au GERTs, respectively. As a result, the Raman scattering cross-section per particle was enhanced by 1,350-fold for BBT NPs compared to NBT@Au NPs, and by 183-fold compared to NBT@Au GERTs.

**Extended Data Fig. 4:**
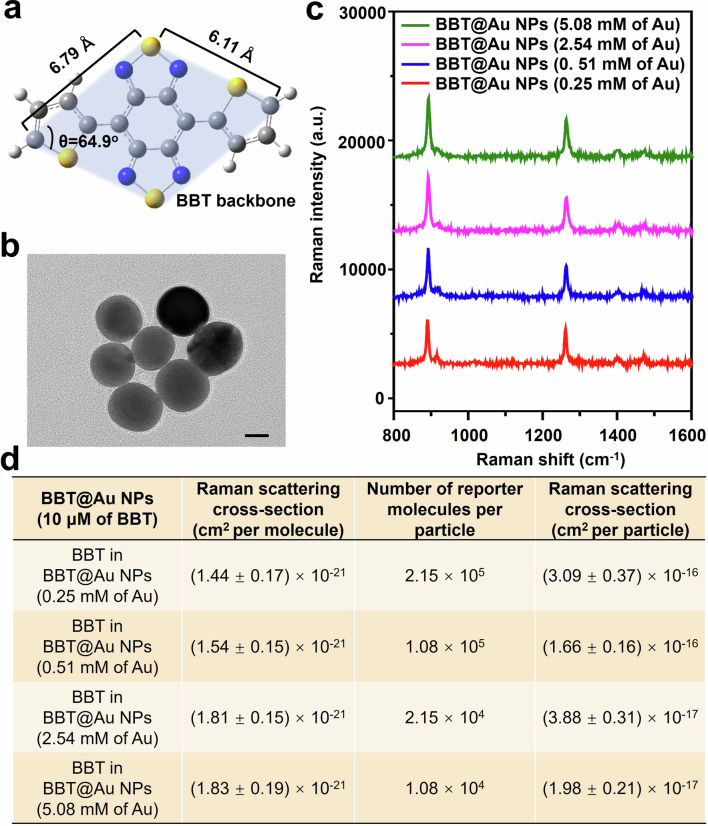
Raman scattering cross-section of SERS-based BBT@Au NPs by coating BBT on the surface of 40 nm-sized Au NPs. **a**, Illustration diagram of BBT with dimensions, optimized by Gaussian 09/B3LYP/6-31 G(d). **b**, Transmission electron micrographs of the Au NPs with a 40 nm of average diameter. Scale bar, 20 nm. Image shown is representative of n = 3 independent replicates of experiments with similar results. **c**, Raman spectra of BBT (10 μM) adsorbed on different concentrations of Au NPs. **d**, Raman scattering cross-section of BBT (10 μM) coated on the surface of different concentrations of Au NPs. Raman measurement was carried out with an 830-nm laser excitation, a 5 × objective, a laser power of 62.6 mW, acquisition time of 1 s, and one time accumulation. The cross-section values per BBT molecule were calculated by measuring the Raman peak at 894 cm^−1^, while using the methanol C-O stretch at 1,020 cm^−1^ as the internal reference. n = 3 independent samples (**d**). Data are presented as the mean ± s.d. (**d**). Source data

Moreover, we quantitatively compared the Raman scattering cross-section of SERS-enhanced BBT with that of SICTERS-enhanced BBT. For this purpose, we coated BBT on the surface of 40 nm-sized Au NPs to obtain SERS-based BBT@Au NPs, which are comparable in size to SICTERS-based BBT NPs (~42 nm in diameter). To ensure that the Raman enhancement effect was solely attributed to the SERS by Au NPs, the number of BBT Raman reporters coated on the Au NPs should not be more than the number forming a monolayer (*n*_*Monolayer*_). According to the geometry of BBT molecule optimized by Gaussian 09/B3LYP/6-31 G(d), the *n*_*Monolayer*_ was calculated to be 1.32 × 10^4^ for an NP of 40 nm in diameter (Extended Data Fig. 4a and Methods). BBT molecules (10 μM) were then absorbed on a series of concentrations of the Au NPs from 0.25 mM to 5.08 mM (Extended Data Fig. 4b,c). The theoretically calculated *n*_*Monolayer*_ was close to the actually measured numbers of 2.15 × 10^4^ per particle when 2.54 mM Au NPs were added, and 1.08 × 10^4^ per particle when 5.08 mM Au NPs were added. This finding was in line with the result that these two concentrations of Au NPs generated almost same Raman scattering cross-sections per BBT molecule, that is, 1.81 × 10^−21^ and 1.83 × 10^−21^ cm^2^ (Extended Data Fig. 4d). Because higher BBT loading in the 2.54 mM of Au NPs than that in the 5.08 mM of Au NPs, BBT in the 2.54 mM of Au NPs was used as the SERS-based BBT@Au NPs for the comparative study. The Raman scattering cross-section per BBT molecule measured in the SICTERS-based BBT NPs (diameter of ~42 nm) was 1.73 × 10^−21^ cm^2^ (Extended Data Fig. 3e), whereas the Raman scattering cross-section per BBT molecule measured in the SERS-based BBT@Au NPs (diameter of ~40 nm) was 1.81 × 10^−21^ cm^2^ (Extended Data Fig. 4d), suggesting that the Raman enhancement effect of SICTERS on each BBT molecule is similar to that of Au NP-based SERS. However, the BBT loading number in SICTERS-based BBT NPs (2.32 × 10^5^ per particle) was higher than that in SERS-based BBT@Au NPs (2.15 × 10^4^ per particle). As a result, the Raman scattering cross-section per particle in SICTERS-based BBT NPs (4.01 × 10^−16^ cm^2^ per particle) was 10.3-fold higher than that per particle in SERS-based BBT@Au NPs (3.88 ×10^−17^ cm^2^ per particle) (Extended Data Figs. 3e and 4d).

To explore whether the Raman enhancement of BBT was attributed to the *J*-aggregation-enhanced Raman scattering, we further compared the Raman scattering cross-section of BBT NPs with that of typical *J*-aggregates of astaxanthin (AXT) (Supplementary Fig. 17a)^30^. Compared with the AXT standalone molecules, the Raman scattering cross-section per AXT molecule in the *J*-aggregates was only increased by 2.29-fold, to a value of 1.008 × 10^−24^ cm^2^ per molecule (Supplementary Fig. 17b)^30^. By contrast, the Raman scattering cross-section for BBT molecule within BBT NPs was 1.61 × 10^−21^ cm^2^ per molecule (Fig. 4d), which was 1,597 times larger than that for the AXT molecule in the *J*-aggregates. The comparative data demonstrated that SICTERS but not the *J*-aggregate-induced Raman enhancement was the predominant contributor to the Raman signal enhancement in the BBT NPs.

**Extended Data Fig. 5:**
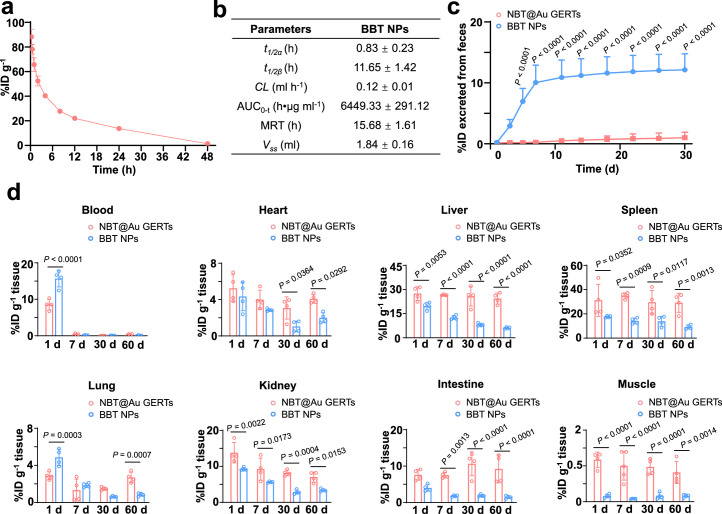
Pharmacokinetics (PK) and biodistribution of BBT NPs in mice. **a**, The plasma concentration-time curve of BBT in ICR mice following the i.v. injection of BBT NPs. **b**, The calculated PK parameters in (**a**). **c**, Cumulative BBT or Au excretion profile in feces from the mice following the injection. **d**, Biodistribution profile of NBT@Au GERTs and BBT NPs in the mice at 1, 7, 30 and 60 d after the i.v. injection, respectively. %ID g^−1^ tissue, percentage of the injected dose per g tissue. The injection doses for all the above experiment: BBT NPs (40 mg kg^−1^ of BBT) and NBT@Au GERTs (40 mg kg^−1^ of Au). n = 5 **(a,b**) and n = 4 or 5 (**c,d**) biologically independent samples. Data are presented as the mean ± s.d. (**a,b,c,d**), and two-way ANOVA with Sidak’s post hoc test was used in (**c,d)**, with *P* values indicated on the graphs. *P* < 0.05 was considered statistically significant. ID, injected dose. *t*_*1/2α*_, distribution half-life. *t*_*1/2β*_, elimination half-life. *CL*, clearance. AUC_0-t_, area under the concentration-time curve to the least measurable concentration. MRT, mean residence time. *V*_*ss*_, steady-state volume of distribution. Source data

We next investigated the pharmacokinetic parameters of BBT NPs in mice after intravenous (i.v.) injection. The plasma concentration versus time curve fit the two-compartment model (Extended Data Fig. 5a). BBT NPs underwent a rapid distribution in the distribution phase (*t*_*1/2α*_ = 0.83 h), followed by a prolonged clearance in the elimination phase (*t*_*1/2β*_ = 11.65 h) (Extended Data Fig. 5b). The biodistribution results showed that BBT NPs were metabolizable as the concentrations of BBT in major organs decreased over time within 60 d after injection (Extended Data Fig. 5c,d). By contrast, the concentrations of the Au substrates of NBT@Au GERTs in most organs except kidney remained unchanged between day 1 and day 60 after injection (Extended Data Fig. 5c,d).

BBT NPs did not show cytotoxicity to NIH 3T3 fibroblast cells at a concentration up to 400 μg ml^−1^ (Supplementary Fig. 18a). Hematologic analysis showed the majority of the hematologic parameters were in the reference ranges within 60 d after injection of BBT NPs (Supplementary Table 3). Only one value of hematocrit at day 7 and two numbers of mean corpuscular volume at days 1 and 30 slightly exceeded the reference ranges but were back to the normal ranges at day 60 (Supplementary Table 3). Blood chemistry analysis of liver function (alanine aminotransferase and aspartate aminotransferase levels) and kidney function (blood urea nitrogen levels) showed that all values were all maintained in the normal ranges (Supplementary Fig. 18b). In addition, histologic analysis did not show any pathologic changes in the major organs of mice treated with BBT NPs (Supplementary Fig. 18c). These data collectively demonstrated that BBT NPs at the imaging dose are biosafe within 60 d after injection.

### Intraoperative real-time imaging of microtumors

**Fig. 5:**
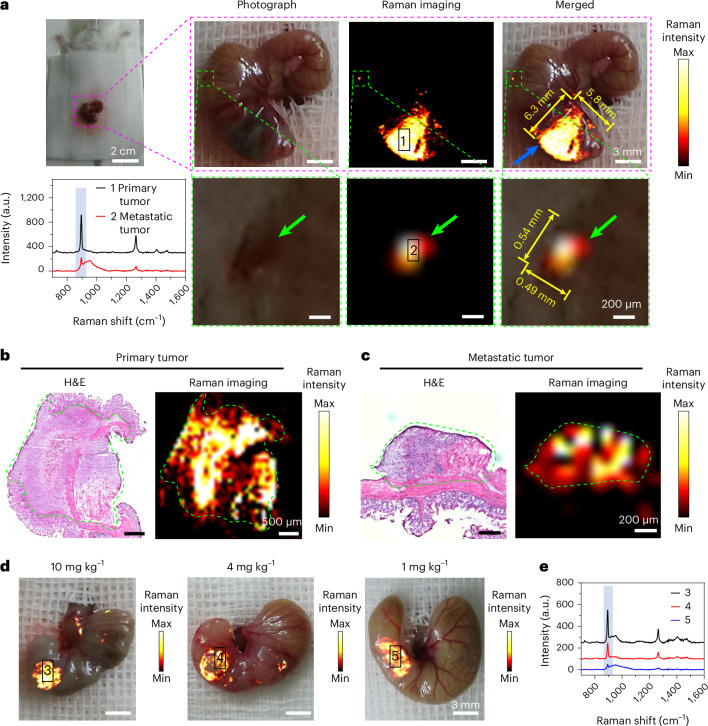
Intraoperative Raman imaging of orthotopic mouse colon tumor by SICTERS. **a**, Live Raman imaging (894 cm^−1^) of primary and metastatic CT26-Luc tumor lesions of mice following the i.v. injection of BBT NPs (40 mg kg^−1^ of BBT). When establishing the orthotopic colon tumor model, CT26-Luc cells were injected into the cecum wall of mice. The tumor formed at the injection site is referred to as primary tumor (blue arrow), while the tumor arising outside the injection site is referred to as metastatic tumor lesion (green arrow). Lower left, Raman spectra of site 1 (primary tumor) and site 2 (metastatic tumor), respectively. **b**,**c**, Histological analysis of primary (**b**) and metastatic (**c**) tumor sections following the live imaging in panel **a**. Green circles, tumor. Left, H&E staining. Right, Raman imaging (894 cm^−1^). Image shown is representative of *n* = 3 independent replicates of experiments with similar results (**b** and **c**). **d**, Intraoperative Raman imaging (894 cm^−1^) of the tumor-bearing mice following i.v. injection of BBT NPs at different doses (calculated by BBT). **e**, Raman spectra of different sites in panel **d**. Blue columns in the above spectra, peaks at 894 cm^−1^. Raman measurement was carried out with a 5 × objective, 830-nm laser excitation, laser powers of 31.3 mW (**a** and **d**) or 62.6 mW (**b** and **c**), acquisition time of 0.3 s and one time accumulation.

Intraoperative real-time detection of microtumors is considered to be one of the most promising applications of the SERS-based Raman imaging. Many researchers including our group have designed a variety of SERS nanoprobes towards this goal^27,31–33^. Using our previously reported intraoperative confocal Raman imaging platform^31^, we performed a comparative study of SICTERS- with SERS-active nanoprobes in mice bearing orthotopic CT26-Luc colon tumor. In the mimic surgical scenario, intraoperative SICTERS imaging based on BBT NPs produced intense Raman signals in the blood vessels at 15 min or 4 h after i.v. injection, whereas a clear tumor image was obtained at 24 h (Supplementary Fig. 19), most likely because the plasma concentration of BBT at 24 h after injection decreased to 15.6% of that at 15 min and tumor accumulation of BBT NPs was sufficient (Extended Data Fig. 5a and Supplementary Fig. 20). The imaging distinguished the primary tumor (5.8 mm × 6.3 mm, blue arrow) and satellite metastatic lesion (0.49 mm × 0.54 mm, green arrows) from the normal tissues in a real-time manner, with the strong characteristic signal intensity at 894 cm^−1^ (Fig. 5a). Hematoxylin and eosin (H&E) staining confirmed these Raman signal-positive areas to be tumor lesions (Fig. 5b,c). The minimum dimension of metastatic microtumor detected by SICTERS imaging was as small as ~0.25 mm × 0.35 mm (Supplementary Fig. 21a,b). This size is comparable to the reported sizes of abdominal tumor metastases detected by SERS^31,33^. By contrast, the control group injected with phosphate-buffered saline (PBS) did not yield any signature Raman peak (Supplementary Fig. 21c,d). To investigate the tumor tissue penetration of BBT NPs, we isolated the microtumor of ~2.5 mm in length from the mice at 24 h post-injection of BBT NPs, and examined them by cryosectioning at different tumor depths (Supplementary Fig. 22a). BBT NPs can penetrate into the core of the microtumor and distribute throughout the tumor cross-section at the penetration depth of 1,258 μm (Supplementary Fig. 22b). Such depth of tumor tissue penetration of the BBT NPs was sufficient for the intraoperative detection of the microtumors in the orthotopic CT26 colon cancer mouse model. For the SICTERS imaging, the Raman signal intensity of BBT in tumor was in a dose-dependent manner, with a minimum injection dose as low as 1 mg kg^−1^ BBT validated by six repeated experiments (Fig. 5d,e and Supplementary Fig. 23). Comparatively, the minimum dose of NBT@Au GERTs was 4 mg kg^−1^ Au for the intraoperative detection of the tumor (Supplementary Fig. 24a–c). A lower dose of NBT@Au GERTs (1 mg kg^−1^ of Au) was unable to provide any Raman signals in the tumor (Supplementary Fig. 24d–f). Notably, the band at 950 cm^−1^ was the background noise; it appeared when the Raman signal of BBT NPs was relatively low in the metastatic tumor (Fig. 5a, spectra 2) or in the primary tumor with low accumulation of BBT NPs (Fig. 5e, spectra 3 and 5). Accordingly, the SICTERS-based BBT NPs produce an equivalent sensitivity for the intraoperative tumor detection at a lower administration dose in comparison with the SERS-based NBT@Au GERTs.

**Extended Data Fig. 6:**
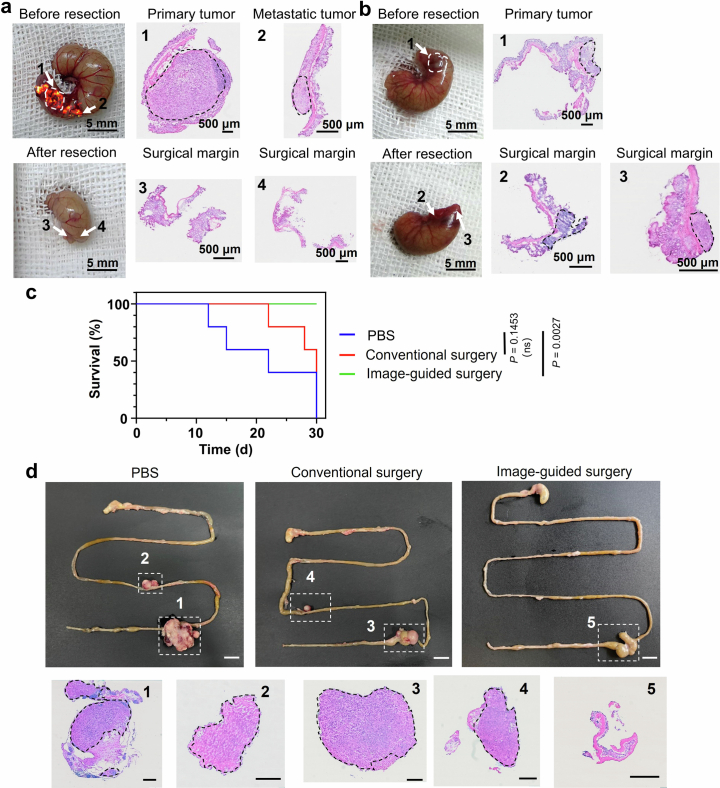
Intraoperative SICTERS-based Raman image-guided surgery for orthotopic mouse colon tumor by BBT NPs. Mice bearing orthotopic CT26-Luc tumor were divided into three groups. PBS, mice received PBS without the tumor resection. Conventional surgery, mice received 40 mg kg^−1^ of BBT NPs, followed by the tumor resection with naked eyes after 24 h. Image-guided surgery, mice received 40 mg kg^−1^ of BBT NPs, followed by the intraoperative Raman image-guided tumor resection after 24 h. **a**, The intraoperative Raman imaging-guided tumor resection. White dotted circles indicated the primary tumor. Raman image and histologic analysis with H&E staining showed primary tumor (region 1, black dotted circle) and microtumor/metastatic tumor (region 2, black dotted circle). No tumor cells remained in the surgical margin (regions 3 and 4) after the intraoperative SICTERS-based Raman imaging-guided surgery. **b**, Conventional surgery of tumors by naked eyes. Histologic analysis with H&E staining confirmed the primary tumor (region 1, black dotted circle) and residual tumors in the surgical margin (regions 2 and 3, black dotted circles). Image shown is representative of n = 5 independent replicates of experiments with similar results (**a,b**). **c**, Kaplan–Meier survival curves of mice. *n* = 5 biologically independent mice. Kaplan–Meier survival curves were analyzed using log-rank (Mantel–Cox) test, with *P* values indicated on the graphs. *P* < 0.05 was considered statistically significant. ns denotes no significant difference. **d**, Histological analysis of the resected gastrointestinal tract and tumor or cecum of mice at the end of different treatments in (**c**). Black dotted circles indicated the tumor. Upper bars, 10 mm; lower bars, 1 mm. Image shown is representative of n = 5 independent replicates of experiments with similar results. Source data

We then performed SICTERS-based Raman image-guided surgery in mice bearing orthotopic CT26-Luc colon tumor at 24 h after injection of BBT NPs. Intraoperative SICTERS-based Raman imaging delineated both primary tumor (region 1) and metastasis (region 2) (Extended Data Fig. 6a). Under the guidance of Raman imaging, the tumor tissues were completely removed, evidenced by the surgical margins free of tumor cells by the H&E staining (regions 3 and 4) (Extended Data Fig. 6a). Comparatively, by conventional surgery, only primary tumor (region 1) was visualized by naked eyes (Extended Data Fig. 6b, before resection). After the surgery, the residual tumors remained in the surgical margins (regions 2 and 3, black dotted circles) (Extended Data Fig. 6b, after resection). Moreover, the results of bioluminescence imaging and end point histopathologic analysis confirmed that the mice were free of tumor or metastases following the SICTERS-based Raman image-guided surgery (Supplementary Fig. 25 and Extended Data Fig. 6c,d). These results demonstrated the feasibility of the SICTERS-based Raman image-guided surgery that may be used to improve the precision of tumor resection.

### Noninvasive imaging of lymphatic drainage and blood vessels

**Fig. 6:**
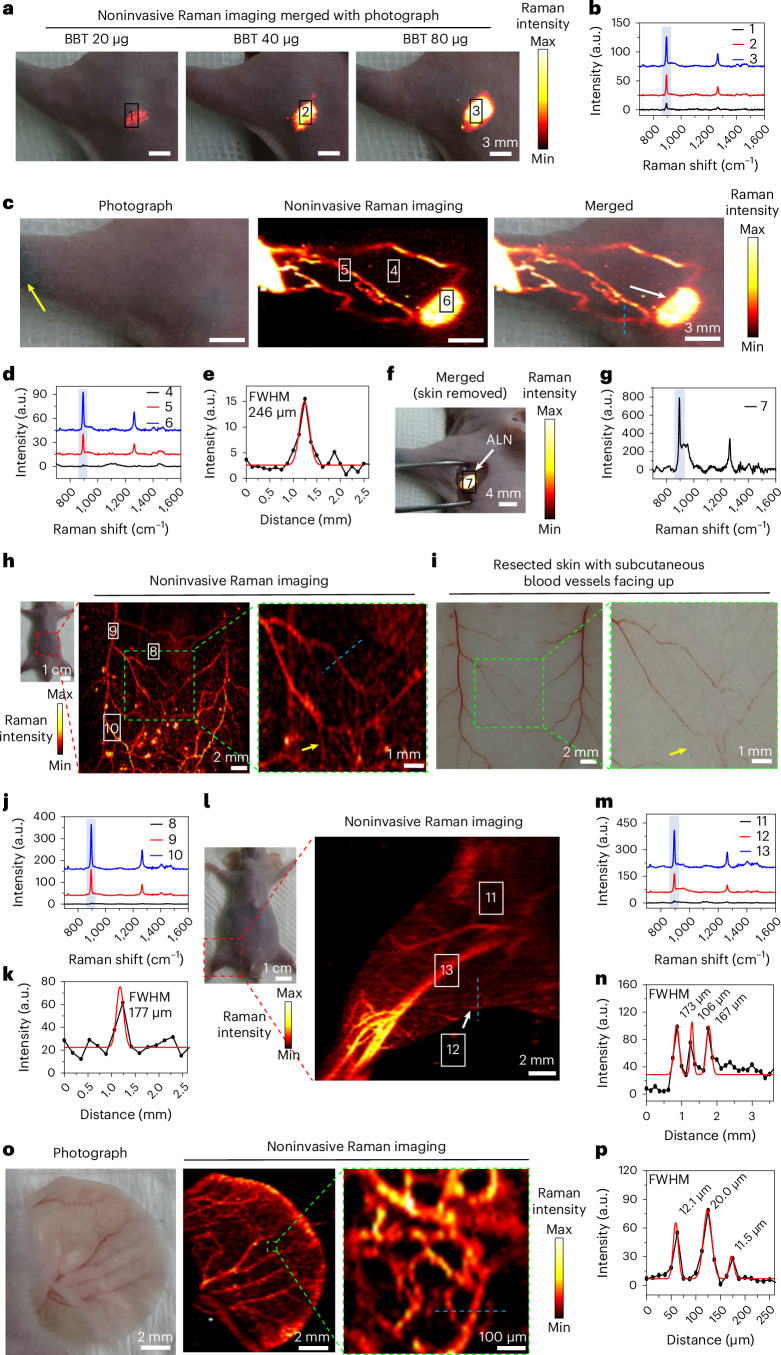
Noninvasive Raman imaging of lymphatic drainage and blood vessels by SICTERS-based BBT NPs. **a**, In vivo Raman imaging (894 cm^−1^) of ALNs merged with photographs of mice after 15 min of the intradermal injection with BBT NPs in the left front paw of nude mice. **b**, Raman spectra of different sites in panel **a**. **c**, Noninvasive Raman imaging (894 cm^−1^) of lymphatic drainage of mice after 15 min of the injection with BBT NPs (150 μg BBT). Yellow arrow, the injection site. White arrow, ALN. **d**, Raman spectra of different sites in panel **c**. **e**, FWHM analysis of lymphatic vessels along the blue dotted line in panel **c**. **f**, Raman imaging (894 cm^−1^) merged with photograph of the exposed ALN with skin removed following the experiment in panel **c**. **g**, Raman spectrum of the ALN in panel **f**. **h**, Noninvasive transdermal Raman imaging (894 cm^−1^) of subcutaneous blood vessels in mouse abdomen following i.v. injection of BBT NPs. Green boxed image, the enlarged area. **i**, The vertically flipped image of the photographs of the resected abdominal skin with the subcutaneous blood vessels facing up, following the live Raman imaging of mice in panel **h** and euthanasia. The yellow arrow in panel **h** represents the Raman imaging signal corresponding to the microvessel indicated by the yellow arrow in panel **i**. **j**, Raman spectra of different sites in panel **h**. **k**, FWHM analysis of micro-blood vessels along the blue dotted line in panel **h**. **l**, In vivo Raman imaging (894 cm^−1^) of blood vessels of mouse hindlimb following i.v. injection of BBT NPs. **m**, Raman spectra of different sites in panel **l**. **n**, FWHM analysis of micro-blood vessels along the blue dotted line in panel **l**. **o**, Noninvasive transdermal Raman imaging (894 cm^−1^) of blood vessels of mouse ear following i.v. injection of BBT NPs. **p**, FWHM analysis of micro-blood vessels along the blue dotted line in panel **o**. Black and red lines in the FWHM analysis, the cross-sectional intensity profiles (894 cm^−1^) of the blood vessels and the Gaussian fitting curves, respectively. Blue columns in the above Raman spectra represent peaks at 894 cm^−1^. Raman measurement was carried out with a ×5 objective, a laser power of 62.6 mW (**a**, **c**, **f**, **h** and **l**) or an LW ×50 objective and a laser power of 22.0 mW (**o**), 830-nm laser excitation, acquisition time of 0.3 s and one time accumulation.

**Extended Data Fig. 7:**
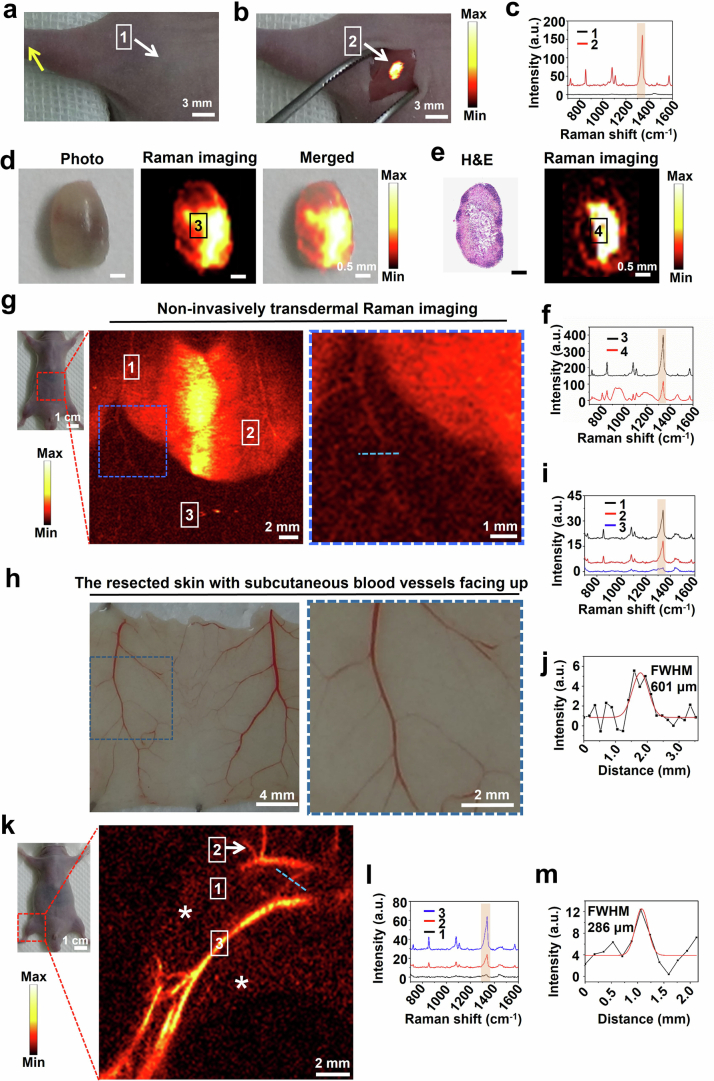
In vivo SERS imaging by NBT@Au GERTs. **a**,**b**, SERS imaging (1340 cm^−1^) merged with photographs of ALNs after 15 min of the intradermal injection with NBT@Au GERTs (80 μg of Au) in the left front paw of mice. Yellow arrow, the injection site. White arrows, ALNs. Raman imaging was performed before (**a**) and after (**b**) removing the skin, respectively. **c**, Raman spectra of sites 1 or 2 in (**a**) or (**b**). **d**, SERS imaging (1340 cm^−1^) of the resected ALN following the imaging experiment in (**b**). **e**, H&E staining (8-μm thickness) and Raman imaging (50-μm thickness) analysis of the adjacent sections of ALN in (**d**), respectively. Image shown is representative of n = 3 independent replicates of experiments with similar results. **f**, Raman spectra of the sites in (**d**) and (**e**), respectively. Orange columns, peaks at 1340 cm^−1^. **g**, SERS imaging (1340 cm^−1^) of mouse abdominal subcutaneous blood vessels following i.v. injection of NBT@Au GERTs (40 mg kg^−1^ of Au). Blue boxed image, the enlarged area. **h**, The vertically flipped image of the photographs of the resected abdominal skin with the subcutaneous blood vessels facing up, after the Raman imaging in (**g**) and euthanasia. **i**, Raman spectra of different sites in (**g**). Orange column, peaks at 1340 cm^−1^. **j**, FWHM analysis of blood vessels along the blue dotted line in (**g**). Black line, the cross-sectional intensity profile (1340 cm^−1^) of the blood vessel. Red line, the Gaussian fitting curve. **k**, Raman imaging (1340 cm^−1^) of blood vessels in leg of mice following i.v. injection of NBT@Au GERTs (40 mg kg^−1^ of Au). Arrow, microvessels. Asterisks, the regions of hindlimb without distinguishable signals of microvessels. **l**, Raman spectra of different sites in (**k**). Orange column, peaks at 1340 cm^−1^. **m**, FWHM analysis of micro-blood vessels along the blue dotted line in (**k**). Black line, the cross-sectional intensity profile (1340 cm^−1^) of the blood vessels. Red line, the Gaussian fitting curve. Raman measurement was carried out with a 5 × objective, 830-nm laser excitation, a laser power of 62.6 mW, acquisition time of 0.3 s and one time accumulation (**a**,**b**,**d**,**g**,**k**) or 1 s in (**e**), and one time accumulation.

**Extended Data Fig. 8:**
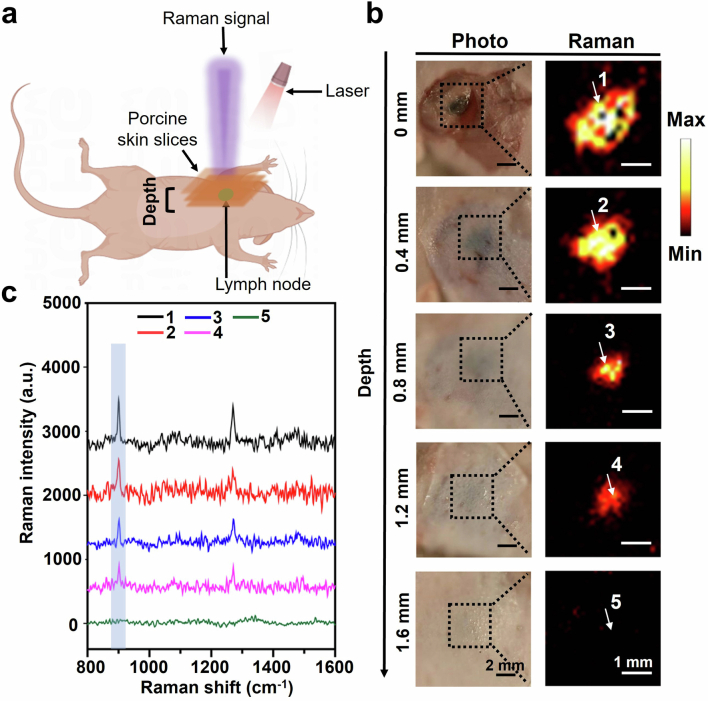
In vivo depth analysis of SICTERS imaging by covering BBT NPs-bearing ALNs with porcine skin slices. **a**, Illustration of the experimental setup. BBT NPs (80 μg of BBT) were injected though the left front paw of mice. Fifteen minutes after injection, ALNs were exposed and then covered with different number of porcine skin slices for imaging. The thickness of each piece of porcine skin slice was 0.4 mm. **b**, The SICTERS-based Raman imaging of BBT NPs-bearing ALNs, covered with porcine skin slices with thicknesses of 0 mm, 0.4 mm, 0.8 mm, 1.2 mm, and 1.6 mm, respectively. **c**, Raman spectra of different regions in (**b**). Blue column, peaks at 894 cm^−1^. Raman measurement was carried out with a 5 × objective, 830-nm laser excitation, a laser power of 62.6 mW, acquisition time of 1 s and one time accumulation.

Noninvasive lymphatic imaging is essential to analyze the functions of lymphatic vessels and understand lymphatic metastasis of tumor. SERS is reported to merely image the exposed lymph nodes when the skin is opened during surgery^34,35^. Consistent with the previous reports, the intradermal injection of NBT@Au GERTs (with 80 μg Au) at the front paw of mice did not offer transdermal Raman imaging of axillary lymph nodes (ALNs) (Extended Data Fig. 7a). Their characteristic Raman mapping was not accomplished until the removal of skin to expose the lymph nodes (Extended Data Fig. 7b–f). Similarly, SERS-based BBT@Au NPs (with 80 µg Au) could not yield any Raman signals unless the skin was removed (Supplementary Fig. 26). By contrast, the injection of BBT NPs at a minimum dose of 20 μg BBT achieved noninvasive transdermal imaging of ALNs (Fig. 6a,b). Notably, the SICTERS-based Raman imaging noninvasively delineated the drainage of BBT NPs from the injection site into the intermodal collecting lymphatic vessels and ALNs (Fig. 6c and Supplementary Fig. 27a). The high S/N ratios of BBT NPs allowed for differentiating ALNs (site 6) or lymphatic vessels (site 5) from their surrounding tissues (site 4) with high resolution (Fig. 6c,d). The cross-sectional intensity profile of the selected lymphatic vessel (Fig. 6c, blue dotted line) showed the full-width at half-maximum (FWHM) of ∼246 μm given by SICTERS (Fig. 6e). The area of positive signals in the Raman imaging correlated well with the H&E staining of the excised ALNs (Fig. 6f,g and Supplementary Fig. 27b,c). Moreover, by covering ALNs with various pieces of porcine skin slices, SICTERS was shown to have a maximum imaging depth of 1.2 mm (Supplementary Fig. 28 and Extended Data Fig. 8). Collectively, SICTERS broadens the application of Raman imaging toward noninvasively depicting lymphatic vessels and lymph nodes.

Optical microangiography by identifying subsurface vascular and structural features provides critical information for diagnosing and monitoring the therapeutic response of skin pathologies, such as psoriasis, hemangiomas or skin cancer^36^. The skin thickness of male BALB/c mice was reported to be 750 ± 30 μm^37^. The i.v. injection of BBT NPs (40 mg kg^−1^ BBT) successfully outlined the microvessels at such depth (Fig. 6h,j and Supplementary Fig. 29). The Raman imaging of subcutaneous microvessel matched well with its photograph (Fig. 6h,i, yellow arrows). The cross-sectional intensity profile illustrated the microvessel across the blue dotted line with the FWHM of 177 μm (Fig. 6h,k). By contrast, SERS with NBT@Au GERTs (40 mg kg^−1^ of Au) offered blurred imaging of the blood vessels with the FWHM of 601 μm, incapable of detection of smaller vessels (Extended Data Fig. 7g–j). Moreover, the vasculatures in mouse hindlimbs with the FWHM as small as 106 μm was clearly visualized following the i.v. injection of BBT NPs (Fig. 6l–n and Supplementary Fig. 30). By comparison, NBT@Au GERTs provided the Raman imaging of large microvessels with the FWHM of 286 μm (Extended Data Fig. 7k–m). NBT@Au GERTs were incapable of depicting smaller vasculatures in leg (Extended Data Fig. 7k, asterisks). These results demonstrated that the SICTERS effect of BBT NPs lights up the subcutaneous and hindlimb microvasculatures of mice unambiguously by noninvasive Raman imaging and affords a superior microangiography to SERS with NBT@Au GERTs. By using a ×50 objective lens, the network of microvessels in the mouse ear with BBT NPs was highlighted with the FWHM as small as 11.5 μm (Fig. 6o,p). Histological analysis did not show any damage to the ear, abdominal or leg skin following SCITERS-based Raman imaging by BBT NPs (Supplementary Fig. 31). SICTERS offers noninvasive and high-resolution Raman imaging of subcutaneous microvasculature in mice.

## Discussion

In summary, we describe the SICTERS effect of small molecules, a principle different from that of Raman scattering enhancement through the previously reported π–π stacking^26,38^. A recent study has shown the photon-induced interfacial charge-transfer processes between the nanostructured film of the organic semiconductor DFP-4T and the Raman reporter molecule methylene blue (MB) through π–π stacking^26^. This intermolecular charge transfer increased the Raman cross-section of MB to 2.4 × 10^−24^ cm^2^ sr^−1^. Besides, a compound [3_4_](1,2,4,5)cyclophane with the intramolecularly π-stacked two benzene rings exhibits 100-fold enhancement of the Raman scattering cross-section to be 9.1 × 10^−25^ cm^2^ (ref. ^38^). Notably, the Raman scattering cross-section of BBT in BBT NPs was measured 1.61 × 10^−21^ cm^2^, which is 670-fold higher than that of MB on the organic semiconductor film and 1,769-fold higher than that of [3_4_](1,2,4,5)cyclophane, respectively. These comparative results indicate that only π-π stacking through the intermolecular charge-transfer between organic semiconductor and reporter molecules or intramolecular charge-transfer was not sufficient to contribute such huge enhancement of the Raman scattering cross-section of BBT. Distinctively, in the substrate-free SICTERS, the spatial arrangement of small molecules provides the intermolecular charge transfer between the D and A units of two adjacent molecules, through backbone-to-backbone in the in-plane or *c* axis direction as well as the π-π stacking in the out-of-plane direction (Fig. 3j and Supplementary Fig. 15g). As a result, charge transfer occurs freely among these excited small molecules in multiple directions and dimensions. The three-dimensional ‘supramolecular’ D-A structure remarkably enhances the effect of charge transfer than the reported π–π stacking.

SICTERS offers two advantages over current SERS toward in vivo applications of Raman imaging. First, SICTERS does not require substrate enhancement, thus avoiding the biosafety concern of the inorganic substrates used in SERS. Our comparative study shows that SICTERS-active small molecules can be metabolized, whereas SERS-based Au substrates are nonmetabolizable (Extended Data Fig. 5c,d), consistent with the previous report that Au is considered as non-biodegradable^11^. Second, SICTERS outperforms SERS in in vivo imaging sensitivity, spatial resolution and imaging depth. Currently reported SERS techniques are limited to superficial detection of the Raman signals of the exposed tumor or lymphatic drainage in an intraoperative scenario^35,39^. The Raman scattering cross-section per particle of SICTERS-based BBT NPs surpassed that of the most sensitive SERS-based NBT@Au GERTs. This finding was attributed to the fact that BBT NPs have a comparable Raman scattering cross-section per molecule and much higher loading efficiency for the reporter molecules compared to NBT@Au GERTs. This advantage of SICTERS enables noninvasive transdermal imaging of lymphatic drainage and microvessels with a FWHM of 11.5 μm with high resolution, which has not been achieved by SERS techniques.

In addition to the substrate-free Raman imaging technique of SICTERS, stimulated Raman scattering (SRS) and coherent anti-Stokes Raman scattering (CARS) represent advanced techniques capable of achieving substrate- and label-free Raman imaging, by relying on two synchronized laser pulses (pump light and Stokes light) to gain signal amplification through optical coherence and vibrational resonance^40,41^. Due to their differential enhancement principles, SICTERS offers the advantages of higher detection sensitivity and imaging depth compared with SRS or CARS techniques. Specifically, the detection sensitivity of SRS or CARS is reported to be in the range of 0.2 mM to 29 mM^42–45^. Indeed, using an alkyne-based probe (for example, the 1-dodecyne molecule in our experiments), which is widely used and considered to be the most sensitive SRS/CARS imaging probe^46^, we measured its sensitivity to be 7 mM by SRS imaging (Supplementary Fig. 32). By contrast, the sensitivity of BBT NPs was 1.25 pM by SICTERS imaging (Supplementary Fig. 33), which is about 10^8^ to 10^9^ times higher than that of SRS-based alkyne probes. In addition, the imaging depth of CARS/SRS is generally limited between 0.2 mm and 0.4 mm without tissue clearing, largely because of the strict requirement of tight laser focusing and overlapping between the two wavelengths (pump and Stokes), which is more easily degraded due to the scattering and aberration of the tissue^47,48^. Indeed, by preparing an alkyne-based probe (that is, 1-dodecyne NPs, referred to as DOD NPs) with a formulation and size similar to that of BBT NPs, the imaging depth of SRS/CARS imaging in biological tissues was verified to be less than 0.4 mm (Supplementary Fig. 34). In contrast, the imaging depth of SICTERS-based BBT NPs in biological tissues can reach 1.2 mm, and thus, SICTERS can noninvasively image ALNs in mice (Extended Data Fig. 8 and Fig. 6a–e).

More generally, as a complement to fluorescence imaging, SICTERS-based Raman imaging offers the advantages in intraoperative multiplex imaging, mainly because Raman spectra consist of narrower peaks compared to broadband fluorescence spectra, with a FWHM of 1–2 nm^27^. The unique ultranarrow spectral peaks distinguish different Raman probes from each other and from the biologic background, allowing them to be detected simultaneously at the same excitation wavelength. Indeed, by a sequential i.v. injection of two different SICTERS nanoprobes, we were able to simultaneously delineate the blood vessels and tumors in an orthotopic colon tumor model with a single laser excitation, respectively (Supplementary Fig. 35), which may favor the guidance of intraoperative tumor resection without damaging the blood vessels.

Despite the above-mentioned advantages, SICTERS bioimaging was limited by its imaging depth. The imaging depth of NIR-II fluorescence imaging has been demonstrated to exceed 1 cm^49^, whereas the imaging depth of SICTERS is measured to be ~1.2 mm. Because human skin, including epidermis and dermis, ranges from 1 to 2 mm^50^, noninvasive SICTERS imaging may not be possible when the depth of human skin or subcutaneous lesions exceeds 1.2 mm. To realize a deeper penetration, further experiments will be focused on the molecular design of SICTERS probes to enhance the Raman scattering cross-section and improve sensitivity. Positron emission tomography and magnetic resonance imaging are whole-body scans that provide deep anatomical localization of tissues. The complementary nature of preoperative positron emission tomography/magnetic resonance imaging and intraoperative SICTERS-based Raman imaging is expected to enable accurate preoperative disease diagnosis and precise intraoperative imaging-guided surgery.

## Methods

### Characterization of the synthetic chemicals

Ultraviolet-visible-NIR absorption spectra were recorded on a UV-2401PC UV/vis spectrophotometer (Shimadzu). The NIR fluorescence emission spectra were measured using a fluorescence spectrometer (PTI QM40). Specular XRD was performed on a D2 PHASER diffraction system (Bruker) with a Cu Kα source, with specular measuring out-of-plane alignment. Samples were approximately 10 mm × 10 mm in size and were measured with an integration time of 4 min per degree for specular XRD. The XRD data were analyzed using MDI Jade 6 software. GIWAXS measurements were carried out using a small/wide-angle X-ray scattering system (Xenocs) with 8 keV primary beam, 0.15° incidence angle and a Pilatus3R detector. The samples for GIWAXS were dissolved in chloroform and deposited onto silicon substrates via thermal evaporation. The crystal structures were analyzed using Mercury 3.8 software.

### DFT calculations

DFT calculations are performed with Gaussian 09 software using the B3LYP functional in combination with 6-31 G(d) basic set. The polarizable continuum model was introduced to consider the effects of the water. Maps of ESP surfaces, highest occupied molecular orbitals, lowest unoccupied molecular orbitals, dihedral angles, polarizability values and Raman spectra of these molecules were obtained based on their optimized S_0_ geometries. Vibrational assignments were analyzed using the VEDA4 program, which generates an optimized set of internal coordinates based on structures of molecules.

### Calculation of the cross-sections for Raman scattering

Raman scattering cross-sections under the excitation of 532-, 785- and 830-nm lasers were obtained using the following equations^51,52^:

1 $${\sigma }_{\rm{s}}={\sigma }_{\rm{r}}\left(\frac{{I}_{\rm{s}}}{{I}_{\rm{r}}}\right)\left[\frac{{v}_{0}{({v}_{0}-{v}_{\rm{r}})}^{3}}{{v}_{0}{({v}_{0}-{v}_{\rm{s}})}^{3}}\right]\left(\frac{{C}_{\rm{r}}}{{C}_{\rm{s}}}\right)L({v}_{0})$$

2 $$L({v}_{0})={\left(\frac{{n}^{2}+2}{3}\right)}^{4}.$$

In the equations, *σ*_s_ and *σ*_r_ are the Raman scattering cross-sections of samples and the reference, respectively. *I*_s_ and *I*_r_ are the Raman intensities of samples and the reference, respectively. *C*_s_ and *C*_r_ are the concentrations of samples and the reference in the solution, respectively. *v*_0_ is the wavenumber (cm^−^^1^) of the excitation laser. *v*_s_ and *v*_r_ are the Raman shifts from samples and the reference, respectively. *L*(*v*_0_) is the local field correction factor, and *n* is the refractive index of the solvent. In our case, the methanol C–O stretch at 1,020 cm^−^^1^ was used as the internal reference, with a reported Raman cross-section value of 1.6 ×10 ^−29^ cm^2^ per molecule^53,54^.

### Molecular docking of BBT bound to HSA

The X-ray structure of HSA was downloaded from the Protein Data Bank (PDB) database (https://www.rcsb.org/, PDB code: 6wuw). The structures were prepared by SYBYL-6.9 (including residue repair and energy minimization) using the following method: Powell; termination: gradient with 0.001 kcal mol^−1^; max iterations: 5,000; force field: Tripos; charges: Gasteiger-Huckel.

### Preparation and characterization of BBT NPs or 4-NBT NPs

The THF mixture containing 1 mg of BBT and 1 mg of DSPE-PEG was poured into water with 10-fold volume. The THF/water mixtures were then sonicated for 2 min (sonicate for 3 seconds, then stop for 3 seconds, 20 cycles) using an ultrasound sonicator (Scientz) at 20 W or 50 W of the output power for the preparation of NPs with different sizes. After THF evaporation by stirring the mixture in fume hood for 8 h, the resulting BBT NPs (5 ml, 0.2 mg ml^−1^ of BBT) were obtained by filtration through a 0.22-µm filter. 4-NBT NPs were prepared in the same method as BBT NPs, except that 1 mg 4-NBT and 0.5 mg DSPE-PEG were used and the output power of ultrasound sonicator was 40 W. The particle size was measured using a dynamic light scattering (DLS) analyzer (Malvern 3000, UK) with the Zetasizer 7.12 software.

### Measurement of the number of NPs

The number of BBT NPs and BBT@Au NPs were measured by NP tracking analysis using ZetaView PMX 110 (Particle Metrix). Briefly, the prepared BBT NPs and BBT@Au NPs were diluted to 10^7^–10^8^ particles ml^−1^ and injected into the sample tank using a syringe. The software ZetaView 8.04.02 (SP2) was used for data acquisition and analysis. To determine the number of NBT@Au NPs and NBT@Au GERTs, Au cores (~22 nm) were quantified based on an absorbance at 450 nm according to a previous report^55^. Then, the Au cores (~22 nm) were used to prepare the 70 nm-Au NPs or Au GERTs according to the above-described method. The number of Au cores (~22 nm) was used to quantify the number of particles. The measurement was repeated three times for each sample.

### Number of BBT molecules in a single-layer coating on Au NP

The number of BBT molecules in a monolayer coating on each Au NP was calculated. Considering the average particle size of Au NPs was 40 nm in diameter, the surface area per Au NP was calculated to be ~5,024 nm^2^ (*S*_*Au*_ = 5,024 nm^2^) according to Eq. 3.

3 $${S}_{\rm{Au}}=\uppi \,{d}^{2}$$

where *S*_Au_ is the surface area of per particle and *d* is the diameter of Au NPs.

According to the geometry of BBT molecule optimized by Gaussian 09/B3LYP/6-31 G(d), the backbone structure of BBT was considered to be a planar conformation. Assuming the planar structure (parallelogram) of the BBT backbone attached on the NP surface without consideration of the flexible side chains, the attached area was calculated to be ~0.38 nm^2^ per molecule (*S*_BBT_ = 0.38 nm^2^) based on Eq. 4.

4 $${S}_{\rm{BBT}}=a\times b\times \sin\theta,$$

where *S*_BBT_ is the surface area of BBT molecule, *a* and *b* are the lengths of two different sides in the parallelogram, respectively and *θ* is the angle between the two adjacent sides.

Therefore, the number of BBT molecules coated on each Au NP to form a monolayer (*n*_Monolayer_) was calculated to be 1.32 × 10^4^ per particle according to Eq. 5.

5 $${n}_{\rm{Monolayer}}={S}_{\rm{Au}}/{S}_{\rm{BBT}}.$$

### Stability test of BBT NPs in vitro

CT26 cells were collected (1 × 10^7^ cells) for the preparation of the lysosomal extract using the lysosomal extraction kit (BestBio). The lysosomal extract was mixed with 1 ml PBS and frozen-thawed to obtain the lysosomal lysate. BBT NPs (0.6 g liter^−1^) were added to PBS, 10% fetal bovine serum or the lysosomal lysate for incubation at 37 °C or room temperature. DLS analysis and Raman intensity measurement at 894 cm^−1^ were done at various time points after incubation. Raman measurement was carried out with a ×5 objective, 785-nm laser excitation, a laser power of 31.3 mW, acquisition time of 0.8 s and one time accumulation.

### Raman measurements

For all in vitro and in vivo studies, Raman spectra or images were acquired using an inVia Raman microscope with a 1,040 × 256 pixel charge-coupled device detector (Renishaw). This confocal Raman spectroscopy was equipped with 532 nm, 785 nm and 830 nm lasers and ×5, ×20 and LW ×50 objectives. The Raman images were generated and analyzed by a signal to baseline algorithm (WiRE 4.3 software, Renishaw). The laser parameters used for the different measurements are detailed in Supplementary Table 4. The laser power density values were calculated according to the formula: laser power density = laser power/laser spot area. The laser powers used for SICTERS-based in vivo applications ranged from 22 mW to 84.5 mW, which was consistent with previously reported laser power values of confocal Raman spectroscopy used in clinical applications (ranging from 23 mW to 100 mW)^56–59^.

### Animal studies

All animal experiments were performed at the Experimental Animal Center of Shanghai Jiao Tong University School of Medicine, with housing conditions of 12-h light/12-h dark cycle, 24 °C and 50% humidity. Animal procedures were in agreement with the guidelines of the Institutional Animal Care and Use Committee of Shanghai Jiaotong University School of Medicine. Crl:CD1 (ICR) mice (female, 4–6 weeks), BALB/c mice (male, 6–8 weeks) and nude mice (male, 6–8 weeks) were purchased from Shanghai SLAC Laboratory Animal Co. During the imaging experiment, mice were anesthetized by inhalation of 2% isoflurane.

### Pharmacokinetics of BBT NPs

ICR mice were i.v. injected with BBT NPs (40 mg kg^−1^ BBT) (*n* = 5). Blood was collected using heparinized tubes at 0.25, 0.5, 1, 2, 4, 8, 12, 24 and 48 h, respectively. Blood samples were centrifugated at 1,503 × *g* for 10 min to collect plasma. Each plasma sample (10 μl) was diluted with 90 μl PBS and extracted by toluene. The fluorescence intensity of BBT (excitation = 830 nm, emission = 1,020 nm) was measured using a fluorescence spectrophotometer (PTI QM40). Pharmacokinetic parameters of BBT NPs were calculated using PKSolver 2.0 software (China Pharmaceutical University).

### Intraoperative Raman imaging of the CT26-Luc colon tumor

The orthotopic CT26-Luc colon tumor model was established in BALB/c mice (male, 6–8 weeks) according to our previously reported method^60^. Tumor growth was monitored by bioluminescence after intraperitoneal injection of 3 mg d-luciferin potassium salt in 200 μl PBS using the IVIS Spectrum Imaging System (Caliper, PerkinElmer), and data were analyzed with Living Image Software (version 4.4, PerkinElmer). 4 d after tumor cell inoculation, mice were i.v. injected with BBT NPs in PBS containing 40 mg kg^−1^ BBT. To test the minimum dose of BBT NPs for imaging, BBT NPs in PBS with 1, 4 and 10 mg kg^−1^ BBT were injected, respectively. To measure the minimum dose of NBT@Au GERTs for imaging, NBT@Au GERTs in PBS with 1 and 4 mg kg^−1^ Au were injected, respectively. 24 h after injection, mice received an abdominal skin incision under anesthesia. The cecum was exposed for Raman imaging. In the control group, the tumor-bearing mice were i.v. injected with PBS (200 μl). The cecum was exposed for Raman imaging 24 h after the injection. Raman scanning was performed in StreamLine high-speed acquisition mode with 124.8 μm step size and 0.3 s acquisition time. Characteristic peak at 894 cm^−1^ for BBT NPs or 1,340 cm^−1^ for NBT@Au GERTs was selected for the image processing.

The resected tumor tissue was embedded in OCT (Sakura Finetek) for frozen sectioning (50 μm thickness). Raman imaging of the tissue sections was acquired using the Renishaw Streamline function, 830-nm laser, 87.4 μm step size and 0.3 s exposure time. Adjacent tissue sections with a thickness of 8 μm were used for H&E staining.

### Intraoperative Raman-imaging-guided surgery

BALB/c mice bearing orthotopic CT26-Luc tumor were randomly divided into three groups: (1) mice receiving BBT NPs and Raman-imaging-guided surgery, (2) mice receiving BBT NPs and surgery based on visual inspection (conventional surgery) and (3) mice treated with PBS as the control group. For the BBT NP injection groups, the mice were i.v. injected with BBT NPs (40 mg kg^−1^ BBT). For the Raman-imaging-guided surgery group, at 24 h after injection, intraoperative Raman imaging of the tumor was performed according to the described method above. Primary bulk tumor and microtumor were removed under the guidance of Raman imaging and confirmed by H&E staining. Tumor growth was monitored by bioluminescence intensity using the IVIS Spectrum Imaging System (Caliper, PerkinElmer) within 30 d after treatment, and data were analyzed with Living Image Software (version 4.4, PerkinElmer). Tumor and normal colon tissue were collected at the end point of the experiment and sectioned for H&E staining.

### Imaging depth of BBT NPs

To measure the maximum imaging depth of BBT NPs in biological tissues, the porcine skin was sectioned into slices with the thickness of ~0.4 mm each. BBT NPs (80 μg BBT) were sealed in glass capillary tube with 1 mm inner diameter. The BBT NP-loaded glass capillary tube was covered with various pieces of porcine skin slices for Raman imaging. For in vivo Raman imaging, nude mice were injected in the left front paw under anesthesia with BBT NPs (80 μg of BBT) in PBS. 15 min after injection, ALNs were surgically exposed and covered with various pieces of porcine skin slices. Raman imaging was conducted in StreamLine high-speed acquisition mode with a ×5 objective, 830-nm laser excitation, laser powers of 62.6 mW, acquisition time of 1 s and one time accumulation. The characteristic peak at 894 cm^−1^ for BBT NPs was used for the image processing.

### Raman imaging of ALNs

For imaging of ALNs and the lymphatic drainage, nude mice (male, 6–8 weeks) were injected in the left front paw under anesthesia with BBT NPs in PBS solution containing 0.15 mg BBT. Imaging was conducted 15 min after injection. To investigate the minimum injection dose for the imaging, equal volumes of BBT NPs solution with 0.02, 0.04 or 0.08 mg of BBT were injected. ALNs were surgically removed under the guidance of the Raman imaging and embedded in OCT for frozen sectioning (50 μm thickness). Raman imaging of the tissue sections was acquired using the Renishaw StreamLine function, 87.4 μm step size and 0.3 s acquisition time. Adjacent tissue sections with a thickness of 8 μm were used for H&E staining.

For Raman imaging of SERS-based BBT@Au NPs, nude mice were injected in the left front paw under anesthesia with the BBT@Au NPs in PBS solution containing 80 μg Au and 0.93 μg BBT. 15 min after injection, ALNs of mice with or without skin removal were subjected to Raman imaging in StreamLine high-speed acquisition mode with a ×5 objective, 830-nm laser excitation, laser powers of 62.6 mW, acquisition time of 0.3 s and one time accumulation. The characteristic peak at 894 cm^−1^ for BBT NPs was selected for image processing.

### Raman imaging of subcutaneous blood vessels

To image vasculatures in the abdominal skin or legs, nude mice were i.v. injected with BBT NPs or NBT@Au GERTs in PBS containing 40 mg kg^−1^ BBT or Au. The mice were immediately subjected to Raman imaging under anesthesia. After imaging, mice were euthanized. The abdominal skin was resected with the subcutaneous blood vessels facing up for photographing. The photograph was vertically flipped to match the Raman image.

To image the microvessels in mouse ear, nude mice were i.v. injected with BBT NPs in PBS containing 40 mg kg^−1^ BBT. Mice were in supine position under anesthesia, with ears fully extended, and subjected to Raman imaging immediately. Raman scanning was performed in StreamLine high-speed acquisition mode with an LW ×50 objective, 12.5 μm step size and acquisition time of 0.3 s.

### Biodistribution of BBT NPs

BALB/c mice bearing CT26-Luc orthotopic tumor were i.v. injected with BBT NPs (40 mg kg^−1^ BBT) and euthanized after 1 h and 24 h, respectively. Blood and major organs were collected and weighted. Liver, kidney or intestine was mixed with PBS (three times the tissue weight). Heart, spleen, muscle or lung was mixed with 1 ml PBS. Tumor was mixed with 300 μl PBS. The mixture was homogenized by a tissue lapping apparatus for 3 min. To extract BBT from different samples, 400 μl toluene was added into 100 μl of the tissue homogenate or plasma sample. The mixture was emulsified by ultrasonic cell crusher (Scientz) and then centrifuged for 10 min at 21,130 × *g*. The supernatant was collected for measurement of BBT (excitation = 830 nm, emission = 1,000 nm) on a fluorescence spectrophotometer (PTI QM40).

In another experiment, ICR mice were randomly divided into two groups and i.v. injected with BBT NPs (40 mg kg^−1^) or NBT@Au GERTs (40 mg kg^−1^). Mice were euthanized at 1, 7, 30 or 60 d after injection. Blood and major organs including heart, liver, spleen, lung, kidney, intestine and muscle were collected and weighted. The analysis of BBT in the samples follows the above procedure. For mice treated with NBT@Au GERTs, blood and tissues were digested with aqua regia. The Au content was quantified by an inductively coupled plasma optical emission spectrometer (ICP-OES, iCAP 7400, Thermo Fisher Scientific).

### Analysis of BBT or Au in feces

ICR mice were randomly divided into two groups and individually housed in metabolic cages. Mice were i.v. injected with BBT NPs (40 mg kg^−1^) or NBT@Au GERTs (40 mg kg^−1^). Feces were collected every day for 1 month. All of the samples were dried and weighted. For mice treated with BBT NPs, the samples were added with 4 times of THF in volume, milled, vortexed and sonicated for 15 min. After centrifugation, the supernatant was collected for measurement of BBT on fluorescence spectrophotometer (PTI QM40). For mice treated with NBT@Au GERTs, samples were digested with aqua regia for ICP-OES analysis.

### Statistics and reproducibility

For quantitative analysis, a minimum of three independent replicates were used. All the in vitro, ex vivo, and in vivo imaging experiments were independently repeated at least three times. Statistical analyses were performed using GraphPad Prism 10.0 software. All values and error bars are reported as mean ± s.d. One-way analysis of variance with Dunnett’s multiple comparisons test or Tukey’s multiple comparisons test was used to determine statistical significance when more than two groups were analyzed, and two-way analysis of variance with Sidak’s post hoc test was used when two parameters were considered. The log-rank (Mantel–Cox) test was used to analyze Kaplan–Meier survival curves. *P* values less than 0.05 were considered significant.

### Reporting summary

Further information on research design is available in the Nature Portfolio Reporting Summary linked to this article.

## Online content

Any methods, additional references, Nature Portfolio reporting summaries, source data, extended data, supplementary information, acknowledgements, peer review information; details of author contributions and competing interests; and statements of data and code availability are available at 10.1038/s41587-024-02342-9.

## Supplementary information

Supplementary Information Supplementary Methods, Figures 1-35, Tables 1-4, and References 1–4.

Reporting Summary

Supplementary Video 1 Ring stretching and bending modes of the multi-ring skeleton of DTBT corresponding to the Raman peak at 894 cm^−1^ (front view).

Supplementary Video 2 Ring stretching and bending modes of the multi-ring skeleton of DTBT corresponding to the Raman peak at 894 cm^−1^ (lateral view).

Supplementary Video 3 Ring stretching and C-H bending modes of the multi-ring skeleton of DTBT corresponding to the Raman peak at 1264 cm^−1^ (front view).

Supplementary Video 4 Ring stretching and C-H bending modes of the multi-ring skeleton of DTBT corresponding to the Raman peak at 1264 cm^−1^ (lateral view).

Supplementary Video 5 C-C stretching modes of IR792 corresponding to the Raman peak at 1202 cm^−1^.

Supplementary Video 6 BFDH (Bravais, Friedel, Donnay and Harker) theoretical crystal morphology of DTBT simulated based on the single-crystal structure from the Cambridge Crystallographic Data Centre (CCDC, code: 1308377).

Supplementary Video 7 Ring stretching and bending modes of the multi-ring skeleton of the two DTBT molecules stacked in the in-plane direction based on their crystal geometries from the CCDC corresponding to the Raman peak at 894 cm^−1^.

Supplementary Video 8 Ring stretching and bending modes of the multi-ring skeleton of the two DTBT molecules stacked in the out-of-plane direction based on their crystal geometries from the CCDC corresponding to the Raman peak at 894 cm^−1^.

Supplementary Video 9 Ring stretching and C-H bending modes of the multi-ring skeleton of the two DTBT molecules stacked in the in-plane direction based on their crystal geometries from the CCDC corresponding to the Raman peak at 1264 cm^−1^.

Supplementary Video 10 Ring stretching and C-H bending modes of the multi-ring skeleton of the two DTBT molecules stacked in the out-of-plane direction based on their crystal geometries from the CCDC corresponding to the Raman peak at 1264 cm^−1^.

Supplementary Data 1 Statistical Source Data.

Supplementary Data 2 Crystallographic data for BBT.

Supplementary Data 3 CheckCIF file for BBT.

## Source data

Source Data Fig. 4 Statistical Source Data.

Source Data Extended Data Fig. 3 Statistical Source Data.

Source Data Extended Data Fig. 4 Statistical Source Data.

Source Data Extended Data Fig. 5 Statistical Source Data.

Source Data Extended Data Fig. 6 Statistical Source Data.

## Data Availability

All data that support the findings of this study are available within the article and its Supplementary Information or from the corresponding author upon reasonable request. Data regarding the X-ray structure of HSA are publicly available on the PDB database (https://www.rcsb.org/structure/6WUW). Source data are provided with this paper.
