# Supplementary material for: Self-stacked small molecules for ultrasensitive, substrate-free Raman imaging in vivo
Source: Nat Biotechnol. 2024 Aug 21;43(6):936–47. doi: 10.1038/s41587-024-02342-9 (PMC12167709; doi:10.1038/s41587-024-02342-9)
Supplement: Supplementary file 1 — Supplementary Methods, Figures 1-35, Tables 1-4, and References 1–4. [file 41587_2024_2342_MOESM1_ESM.pdf]

nature biotechnology

Supplementary information https://doi.org/10.1038/s41587-024-02342-9

Self-stacked small molecules for ultrasensitive, substrate-free Raman imaging in vivo

In the format provided by the authors and unedited

Table of Contents

Supplementary Methods

Supplementary Figs.

Supplementary Fig.1 | UV-Vis absorption spectrum and Raman spectra of BBT excited at different wavelength.

Supplementary Fig. 2 | Schemes of the synthetic routes of the designed compounds.

Supplementary Figs. 3-12 | Characterization of compounds **4**, **5**, **7**, **11**, **12**, **14**, **17**, **19**, **20** and **21**.

Supplementary Fig. 13 | Chemical structures and Raman spectra of respective molecules.

Supplementary Fig. 14 | Increased electron delocalization and reduced energy gap of the DTBT-based D-A-D system.

Supplementary Fig. 15 | The molecular stacking of BBT determined by single crystal X-ray diffraction, with close intermolecular distances allowing for a three-dimensional charge-transfer between the neighboring molecules.

Supplementary Fig. 16 | *In vitro* stability of BBT NPs.

Supplementary Fig. 17 | Absorption spectra and Raman scattering cross-section of AXT *J*-aggregates.

Supplementary Fig. 18 | Biosafety analysis of BBT NPs *in vitro* and *in vivo*.

Supplementary Fig. 19 | Intraoperative Raman imaging of the orthotopic mouse colon tumor by SICTERS following the injection of BBT NPs at different time points.

Supplementary Fig. 20 | Biodistribution of BBT in CT26-Luc orthotopic tumor-bearing mice at 1 h or 24 h after i.v. injection of BBT NPs at 40 mg kg^{-1} of BBT.

Supplementary Fig. 21 | Intraoperative Raman imaging (894 cm^{-1}) of orthotopic CT26-Luc colon tumor in mice following i.v. injection of BBT NPs (40 mg kg^{-1} of BBT) or PBS.

Supplementary Fig. 22 | Tumor tissue penetration of BBT NPs.

Supplementary Fig. 23 | Intraoperative Raman imaging of orthotopic colon tumor by BBT NPs.

Supplementary Fig. 24 | Intraoperative Raman imaging of orthotopic mouse colon tumor by SERS.

Supplementary Fig. 25 | Tumor growth of mice following intraoperative SICTERS-based Raman image-guided surgery.

Supplementary Fig. 26 | *In vivo* Raman imaging of ALNs by BBT@Au NPs and BBT NPs.

Supplementary Fig. 27 | *In vivo* Raman imaging of lymphatic drainage by SICTERS.

Supplementary Fig. 28 | *In vitro* depth analysis of SICTERS imaging by covering BBT NPs-loaded glass capillary tube with porcine skin slices.

Supplementary Fig. 29 | The enlarged SICTERS image of Fig. 6h.

Supplementary Fig. 30 | *In vivo* Raman imaging (894 cm^{-1}) of blood vessels of mouse hindlimb following i.v. injection of BBT NPs.

Supplementary Fig. 31 | Representative histological analysis of ear, abdominal and leg skin of mice with or without receiving Raman imaging by H&E staining.

Supplementary Fig. 32 | Detection sensitivity measurement of stimulated Raman scattering (SRS)-based alkyne probes (1-dodecyne).

Supplementary Fig. 33 | Detection sensitivity measurement of SICTERS-based BBT NPs.

Supplementary Fig. 34 | Depth analysis of SRS or CARS imaging of mouse ALNs using alkyne-based probes.
Supplementary Fig. 35 | Intraoperative multiplexed Raman imaging of tumors and blood vessels using SICTERS-based NPs.

Supplementary Tables

Supplementary Table 1 | Crystal data and structure refinement for mj23030_0m (BBT).
Supplementary Table 2 | The Raman scattering cross-sections for 4-NBT in various nanoparticles under the excitation at the different wavelengths.
Supplementary Table 3 | Hematologic analysis of mice after i.v. injection of BBT NPs.
Supplementary Table 4 | Laser parameters used for different measurements in our SICTERS-based confocal Raman imaging system.

References

Supplementary Methods

Chemical synthesis

Chemical reagents were purchased from Bidepharm, Energy, Adamas-beta or Titan, and used as received or purified according to Purification of Common Laboratory Chemicals. Solvents were purchased from Titan and used directly without further purification. DSPE-PEG was purchased from AVT Pharmaceutical Tech Co., Ltd.

Synthesis of 4,7-di(thiophen-2-yl)benzo[1,2-*c*:4,5-*c*']bis([1,2,5]thiadiazole) (compound 4). To a solution of 4,7-dibromo-benzo [1,2-*c*:4,5-*c*']bis([1,2,5]thiadiazole) (compound **1**, 0.2 g, 0.57 mmol) and tributyl(thiophen-2-yl)stannane (compound **2**, 0.57 g, 1.52 mmol) in 1,4-dioxane (20 ml) was added tetrakis(triphenylphosphin)palladium ($Pd[PPh_3]_4$, 57 mg, 0.05 mmol). The mixture was stirred for 12 h at 105 °C. After being cooled to room temperature, the mixture was poured into saturated aqueous potassium fluoride and extracted with dichloromethane (CH_2Cl_2). The organic layer was washed with brine before being dried over $MgSO_4$. After the evaporation of solvent, the residue was purified by column chromatography on silica gel with CH_2Cl_2: petroleum ether = 1:1 (v/v) as the eluent to afford the targeted product (83 mg, 41%) as a blue solid. ^{1}H Nuclear magnetic resonance (NMR) (600 MHz, THF-d_8) δ 7.58 -7.53 (m, 2H), 7.50-7.48 (m, 2H), 7.43-7.38 (m, 2H). ^{13}C NMR (151 MHz, THF-d_8) δ 150.49, 134.47, 134.16, 133.54, 131.80, 131.25, 131.18, 130.75, 130.15, 129.35, 127.58, 127.50, 126.86, 126.69. MALDI-TOF MS Calcd for: $C_{14}H_7N_4S_4^+$ ($[M+H]^+$): 359.9571. Found: 359.9655.

Synthesis of 4,7-diphenylbenzo[1,2-*c*:4,5-*c*']bis([1,2,5]thiadiazole) (compound 5). Compound **5** was prepared in a manner similar to that of compound **4**. Yield: 42%; purple solids.^{1}H

NMR (600 MHz, $CDCl_3$) δ 8.23 (d, *J* = 7.5 Hz, 4H), 7.69 (t, *J* = 7.7 Hz, 4H), 7.58 (t, *J* = 7.5 Hz, 2H). ^{13}C NMR (151 MHz, $CDCl_3$) δ 152.18, 134.36, 131.10, 128.48, 127.81, 121.04. MALDI-TOF MS Calcd for: $C_{18}H_{11}N_4S_2^+$ ($[M+H]^+$): 347.0420. Found: 347.0637.

Synthesis of benzo[1,2-*c*:4,5-*c*']bis([1,2,5]thiadiazole (compound 7). Benzene-1,2,4,5-tetra amine (compound **6**, 0.15 g, 0.53 mmol), N-sulfinylaniline (0.36 g, 2.64 mmol) and chlorotrimethylsilane (0.57 g, 5.30 mmol) in dried pyridine (15 ml) were stirred at 85 °C for 6 h under an argon atmosphere. After being cooled to room temperature, the mixture was poured into water and the resulting solids were collected by filtration. The crude product was purified by column chromatography (CH_2Cl_2) to give compound **7** as a blue solid (0.49 g, 48%). ^{1}H NMR (600 MHz, $CDCl_3$) δ 7.45 (dt, *J* = 22.0, 7.9 Hz, 1H), 7.01 (dd, *J* = 22.7, 7.5 Hz, 1H). ^{13}C NMR (151 MHz, $CDCl_3$) δ 128.23, 124.52, 124.41, 117.50, 117.38. MALDI-TOF MS Calcd for: $C_6H_3N_4S_2^+$ ($[M+H]^+$): 194.9794. Found: 194.9937.

Synthesis of 2-phenylthiophene (compound 11). In the degassed 1,4-dioxane, 2-bromothiophene (compound **8**, 0.67 g, 4.10 mmol) and phenylboronic acid (compound **9**, 0.60 g, 4.92 mmol) were dissolved. Afterwards, $Pd[PPh_3]_4$ was added quickly. The degassed 2 M of aqueous K_2CO_3 (1.25 ml) was added into the solution, which was further degassed for 10 min by sparging with argon gas. The reaction mixture was heated at 105 °C for 10 h under argon atmosphere. The solvent was removed with rotary evaporator. The residue was dissolved in EtOAc. The organic layer was washed with water, dried over anhydrous Na_2SO_4 and purified by chromatography on a silica gel column (petroleum ether/EtOAc = 40:1, v/v) to obtain compound **11** as a white solid (0.64 g, 98% yield). ^{1}H NMR (600 MHz, DMSO-d_6) δ 7.66 (dd, *J* = 8.2, 1.0 Hz, 2H), 7.55 (dd, *J* = 5.1, 0.9 Hz, 1H), 7.52 (dd, *J* = 3.6, 1.0 Hz, 1H), 7.42 (t, *J* = 7.8 Hz, 2H), 7.31 (t, *J* = 7.4 Hz, 1H), 7.14 (dd, *J* = 5.0, 3.6

Hz, 1H). ^{13}C NMR (151 MHz, DMSO-d_6) δ 143.17, 133.59, 128.95, 128.33, 127.41, 126.53, 125.50, 125.25, 123.53. MALDI-TOF MS Calcd for: $C_{10}H_9S^+$ ([M+H]$^+$): 161.0420. Found: 161.0468.

Synthesis of 1,4-di(thiophen-2-yl)benzene (compound 12). Compound **12** was synthesized in a manner similar to that described above. Yield: 88%; white solids. ^{1}H NMR (400 MHz, $CDCl_3$) δ 7.62 (s, 4H), 7.34 (d, *J* = 3.0 Hz, 2H), 7.29 (d, *J* = 4.9 Hz, 2H), 7.13-7.06 (m, 2H). ^{13}C NMR (151 MHz, DMSO-d_6) δ 143.16, 133.58, 128.96, 128.77, 128.33, 127.42, 126.54, 125.52, 125.25, 123.53. MALDI-TOF MS Calcd for: $C_{14}H_{11}S_2^+$ ([M+H]$^+$): 243.0297. Found: 242.0386.

Synthesis of 5,6-dinitro-4,7-di(thiophen-2-yl)benzo[*c*][1,2,5]thiadiazole (compound 14). 4,7-Dibromo-5,6-dinitro-2,1,3-benzothiadiazole (compound **13**, 0.50 g, 1.31 mmol) and Pd[PPh_3]$_4$ (0.07 g, 0.06 mmol) in anhydrous 1,4-dioxane (10 ml) were added into a round bottom flask charged with nitrogen. Tributyl(thiophen-2-yl)stannane (compound **2**, 1.46 g, 3.92 mmol) was added. The reaction mixture was refluxed for 4 h under nitrogen followed by cooled down to room temperature. The solvent was removed with a rotary evaporator. The residue was dissolved in CH_2Cl_2. The organic layer was washed with water, dried over anhydrous Na_2SO_4 and purified by chromatography on a silica gel column (petroleum ether/EtOAc = 30:1, v/v) to obtain compound **14** as an orange solid (0.35 g, 69% yield). ^{1}H NMR (600 MHz, $CDCl_3$) δ 7.77 (dd, *J* = 5.1, 0.8 Hz, 2H), 7.54 (dd, *J* = 3.7, 0.8 Hz, 2H), 7.26 (dd, *J* = 5.0, 3.8 Hz, 2H). ^{13}C NMR (151 MHz, $CDCl_3$) δ 151.60, 141.26, 133.70, 130.81, 130.37, 128.93, 127.38, 120.87. MALDI-TOF MS Calcd for: $C_{14}H_7N_4O_4S_3^+$ ([M+H]$^+$): 390.9624. Found: 390.8067.

Synthesis of 5,6-dinitro-4,7-di(thiophen-2-yl)benzo[1,2-*c*:4,5-*c*']bis[1,2,5]thiadiazole (compound 17). To a solution of 2,3-dihydrothieno[3,4-*b*] [1,4]dioxine (compound **15**, 1.0 g, 7.03 mmol) in dry THF at -78 °C was added *n*-butyllithium (*n*-BuLi, 1.6 M, 4.84 ml) dropwise. After 1 h,

a solution of tributyltin chloride ($SnBu_3Cl$, 2.48 ml, 9.19 mmol) was added and the reaction mixture was warmed to ambient temperature for 12 h. After the complete conversion of the starting material, the reaction was quenched with water and extracted with CH_2Cl_2. The organic layers were dried over Na_2SO_4 and concentrated under reduced pressure. The crude compound was obtained as orange oil which can be used in the next step without further purification.

A mixture of the crude compound and 4,7-dibromo-5,6-dinitrobenzo[*c*][1,2,5]thiadiazole (1.0 g, 2.24 mmol) in 1,4-dioxane bubbled with argon for 20 min. $Pd[PPh_3]_4$ (0.54 g, 0.47 mmol) was added to the above mixture. The mixture was heated at 105 °C for 12 h under argon atmosphere. Then, the solvent was removed *in vacuo*. The residue was chromatographed (CH_2Cl_2/petroleum ether = 1:2, v/v) on silica gel to obtain compound **16** as an orange solid (0.81 g, 51% yield). ^1H NMR (400 MHz, $CDCl_3$) δ 6.77 (s, 2H), 4.23 (d, *J* = 11.2 Hz, 8H). ^{13}C NMR (151 MHz, $CDCl_3$) δ 151.99, 142.42, 141.88, 140.57, 119.63, 104.89, 103.97, 64.05, 63.74.

Compound **16** (1.20 g, 2.38 mmol), iron powder (1.33 g, 23.75 mmol) in acetic acid (15 ml) and chloroform (15 ml) was mixed and stirred at 80 °C for 4 h under an argon atmosphere. After being cooled to room temperature, the mixture was poured into water, and the resulting solids were collected by filtration and recrystallized by chloroform as yellow solids. Without further purification, the product and N-sulfinylaniline (3.31 g, 23.8 mmol) and chlorotrimethylsilane (2.58 g, 23.8 mmol) in dried pyridine (35 ml) were stirred at 85 °C for 6 h under an argon atmosphere. After being cooled to room temperature, the mixture was poured into water and the resulting solids were collected by filtration. The crude product was purified by column chromatography (CH_2Cl_2/petroleum ether = 1:1, v/v) to give compound **17** as a blue solid (0.37 g, 33%). ^1H NMR (400 MHz, $CDCl_3$) δ 6.77 (s, 2H), 4.35-4.29 (m, 2H), 4.23-4.19 (m, 2H). ^{13}C NMR (151 MHz, $CDCl_3$) δ 152.00, 141.20, 140.58, 112.98, 109.81,

102.96, 64.14, 63.91. MALDI-TOF MS Calcd for: $C_{18}H_{11}N_4O_4S_4^+$ ($[M+H]^+$): 475.9683. Found: 475.9888.

Synthesis of 4,7-bis(4-(2-ethylhexyl)thiophen-2-yl)benzo[1,2-*c*:4,5-*c*']bis[1,2,5]thiadiazole (compound 19, BBT). To a solution of compound **18** (1.0 g, 5.10 mmol) in dry THF at -78 °C was added *n*-BuLi (1.6 M, 4.78 ml) dropwise. After 30 min, a solution of $SnBu_3Cl$ in hexane (1.0 M) was added and the reaction mixture was warmed to ambient temperature for 12 h. After a complete conversion of the starting material, the reaction was quenched with water and extracted with CH_2Cl_2. The organic layers were dried over Na_2SO_4 and concentrated under reduced pressure. The crude product was obtained as orange oil which can be used in the next step without further purification.

A mixture of the crude product above and 4,7-dibromo-benzo [1,2-*c*:4,5-*c*']bis([1,2,5]thiadiazole (compounds **1**, 0.78 g, 2.23 mmol) in 1,4-dioxane was bubbled with argon for 20 min. $Pd[PPh_3]_4$ (0.23 g, 0.20 mmol) was added to the above mixture. The mixture was heated at 105 °C for 12 h under argon atmosphere. Then, the solvent was removed *in vacuo*. The residue was chromatographed (petroleum ether/EtOAc = 20:1, v/v) on silica gel to obtain BBT as a blue solid (0.73 g, 56% yield). ^{1}H NMR (400 MHz, $CDCl_3$) δ 8.67 (s, 2H), 7.28 (s, 2H), 2.66 (d, *J* = 6.8 Hz, 4H), 1.84-1.71 (m, 2H), 1.45-1.31 (m, 16H), 1.05-0.87 (m, 12H). ^{13}C NMR (151 MHz, $CDCl_3$) δ 150.55, 142.29, 136.50, 133.95, 126.50, 113.00, 39.79, 33.97, 31.90, 28.30, 25.07, 22.51, 13.60, 10.30. MALDI-TOF MS Calcd for: $C_{30}H_{37}N_4S_4^-$ ($[M-H]^-$): 582.1924. Found: 582.2315.

Synthesis of 4,7-bis(5-bromo-(4-(2-ethylhexyl)thiophen-2-yl)benzo[1,2-*c*:4,5-*c*']bis[1,2,5] thiadiazole (compound 20). To a solution of BBT (0.11 g, 0.19 mmol) in chloroform at 0 °C was added N-bromosuccinimide (NBS, 0.07 g, 0.39 mmol) in dry N,N-dimethylformamide (DMF) dropwise. After stirring for 5 min, the reaction was quenched with ice-water and extracted with CH_2Cl_2.

The organic layers were washed with saturated aqueous brine before being dried over Na_2SO_4. After the evaporation of solvents, the residue was purified by column chromatography on silica gel with petroleum ether: EtOAc = 120:1 (v/v) as the eluent to afford compound **20** (53 mg, 38% yield) as a blue solid. ^{1}H NMR (400 MHz, $CDCl_3$) δ 8.57 (s, 2H), 2.63 (d, J = 6.8 Hz, 4H), 1.84-1.70 (m, 2H), 1.46-1.33 (m, 16H), 0.98-0.92 (m, 12H). ^{13}C NMR (151 MHz, $CDCl_3$) δ 149.89, 141.62, 136.38, 133.35, 117.31, 111.74, 39.39, 33.21, 31.91, 28.18, 25.16, 22.54, 13.60, 10.32. MALDI-TOF MS Calcd for: $C_{30}H_{37}Br_2N_4S_4^+$ ([M+H]$^+$): 742.0269. Found: 742.0602.

Synthesis of 4,7-bis(4-(2-ethylhexyl)-5-phenylthiophen-2-yl)benzo[1,2-*c*:4,5-*c*']bis[1,2,5] thiadiazole (compound 21). To a solution of compound **9** (24 mg, 0.19 mmol) and compound **20** (56 mg, 0.076 mmol) in 1,4-dioxane (10 ml) was bubbled with argon for 5 min. Then, 2 M K_2CO_3 (0.12 ml) and $Pd[PPh_3]_4$ (8 mg, 0.0076 mmol) were added to the reaction mixture under an argon atmosphere. The reaction mixture was heated at 105 °C for 2 h and then concentrated *in vacuo*. The residue was dissolved in CH_2Cl_2, and washed with water, saturated aqueous brine and dried over $MgSO_4$. The combined organic layers were concentrated, and the resulting product was purified using a column chromatography (petroleum ether/EtOAc = 150:1, v/v) to give the desired compound **21** as a green solid (42 mg, 75%). ^{1}H NMR (400 MHz, $CDCl_3$) δ 8.88 (s, 2H), 7.63 (d, J = 6.9 Hz, 4H), 7.49 (t, J = 7.8 Hz, 4H), 7.41 (t, J = 6.0 Hz, 2H), 2.80 (d, J = 5.9 Hz, 4H), 1.80-1.70 (m, 2H), 1.41-1.27 (m, 8H), 1.23 (m, 8H), 0.89-0.79 (m, 12H). ^{13}C NMR (151 MHz, $CDCl_3$) δ 151.31, 144.77, 139.15, 136.03, 135.79, 134.82, 129.48, 128.53, 127.75, 113.26, 40.56, 32.80, 32.61, 28.69, 25.85, 23.09, 14.14, 10.83, 1.03. MALDI-TOF MS Calcd for: $C_{42}H_{47}N_4S_4^+$ ([M+H]$^+$): 736.2708. Found: 736.1809.

NMR and mass spectra measurement

NMR spectra were measured on a Bruker AV 400 spectrometer or a Bruker AV 600 spectrometer. Chemical shifts (δ) were reported in parts per million (ppm). ^{1}H NMR spectra were recorded at 400 or 600 MHz in NMR solvents (THF-d_8, $CDCl_3$ or DMSO-d_6) and referenced internally to corresponding solvent resonance. ^{13}C NMR spectra were recorded at 151 MHz and referenced to corresponding solvent resonance. All NMR spectra were analyzed using MestReNova 6.1.0-6224 software. Matrix-assisted laser desorption ionization-time of flight mass spectrometry (MALDI-TOF MS) spectra were recorded in positive reflection mode on a 5800 proteomic analyzer using an Nd: YAG laser (Applied Biosystems, USA). All MALDI-TOF spectra were present using Data Explorer (TM) 4.3 software.

Preparation and characterization of Au NPs

Au NPs were synthesized according to our previous work with modification[1,2]. The Au seed solution was prepared by vigorously mixing 10 ml of cetyltrimethylammonium chloride (CTAC) aqueous solution (0.1 M) with 0.5 ml of $HAuCl_4$ (2 mg ml^{-1}) and 0.5 ml of $NaBH_4$ solution (0.02 M). The seed solution was allowed to stand at 30 °C for 1 h and diluted with double distilled water. Then, 20 ml of CTAC aqueous solution (0.1 M), 1 ml of $HAuCl_4$ (2 mg ml^{-1}) and 150 µl of ascorbic acid (0.04 M) were mixed. Under ultrasonication, 100 µl of the seed diluent was injected into the above mixed solution, and allowed to stand for 2 d in the dark to obtain the Au cores (~22 nm).

To prepare Au NPs with a diameter of ~70 nm, the above synthesized ~22 nm Au cores (1 ml) were added to a mixed growth solution containing 10 ml of CTAC solution (0.1 M), 0.6 ml of ascorbic acid (0.04 M) and 0.6 ml of chloroauric acid ($HAuCl_4$, 2 mg ml^{-1}). The mixture was sonicated and placed in the dark for 120 d to obtain Au NPs (~70 nm). To prepare Raman reporter molecule-modified Au NPs (~70 nm), the synthesized Au NPs were washed once and resuspended in 10 ml of ultrapure

water. Then, 1 ml of DMSO solution with 4-NBT (4 mM) was mixed with the Au NPs and sonicated for 30 min. The 4-NBT-loaded Au NPs (NBT@Au NPs) were washed for 3 times with 0.05 M of CTAC solution and resuspended in 0.1 M of CTAC solution.

To prepare the SERS-based BBT@Au NPs, a seed solution of Au NPs was firstly prepared by vigorously mixing 0.5 ml of $HAuCl_4$ (2 mg ml^{-1}) and 10 ml of CTAC (0.1 M) with 0.5 ml of $NaBH_4$ (0.04 M). The resulting seed solution was then left at 30 °C for 1 h, followed by a 10-fold dilution. Subsequently, a mixture containing 20 ml of CTAC (0.1 M), 0.5 ml of $HAuCl_4$ (2 mg ml^{-1}), and 0.5 ml of ascorbic acid (0.04 M) was prepared. To this solution, a small volume (0.1 ml) of the diluted seed solution was added under sonication. The mixture was allowed to settle in darkness for 2 d, resulting in the formation of highly uniform spherical AuNPs with a diameter of ~40 nm. The synthesized Au NPs were washed three times by centrifuge at 16639 × g for 5 min and then dispersed in 10 ml of CTAC aqueous solution (0.5 mM). Then, the corresponding amount of BBT and DSPE-PEG (1.0 mg) in THF (1 ml) was added under vigorous sonication for 5 min. The final concentrations of Au were from 0.25 mM to 5.08 mM. The resulting BBT in Au NPs were washed three times by centrifuge at 8801 × g for 5 min and dispersed in PBS.

Preparation and characterization of NBT@Au GERTs

To prepare Raman reporter molecule-modified Au cores (~22 nm), the above synthesized Au cores were washed once and resuspended in 20 ml of ultrapure water. Then, 1 ml of dimethyl sulfoxide (DMSO) solution with 4-NBT (4 mM) was mixed with the Au cores and sonicated for 30 min. The 4-NBT-loaded Au cores were washed for 3 times with 0.05 M of CTAC solution and resuspended in 0.1 M of CTAC solution.

Au GERTs were synthesized according to the previously reported method with modification[1,3]. The above synthesized 4-NBT-loaded Au cores (1 ml) were added to the mixed growth solution with 10 ml of CTAC solution (0.1 M), 0.25 ml of ascorbic acid (0.04 M) and 0.25 ml of $HAuCl_4$ (2 mg ml^{-1}) under ultrasonication. NBT@Au GERTs were obtained after 15 min. After centrifugation and washed with distilled water, an excess amount of methoxy-poly (ethylene glycol)-thiol (mPEG-SH, 20 kDa) was added and sonicated for 30 min, followed by washing with water for 3 times.

The nanoparticles were examined by a transmission electron microscopy (Talo L120C G2, USA) operated at 120 kV. Raman spectra were collected using an inVia Raman microscope equipped with a 1040 × 256 pixels charge-coupled device detector (Renishaw, UK).

Measurement of the penetration depth of BBT NPs in tumors

BALB/c mice bearing CT26-Luc orthotopic tumor were i.v. injected with BBT NPs (40 mg kg^{-1} of BBT) and euthanized after 24 h. The tumor was resected and embedded in OCT (Sakura Finetek, Japan) for frozen sectioning (50 μm thickness). Raman imaging of the tissue sections at different depths was acquired using the Renishaw Streamline function (inVia Raman microscope, Renishaw, UK), 830-nm laser, 62.6 mW, 5 × objective, 87.4 μm step size and 1 s exposure time. The adjacent tissue section for each depth with a thickness of 8 μm was used for H&E staining.

Preparation and characterization of DOD NPs

THF containing 1 mg of 1-dodecyne and 1 mg of DSPE-PEG was added into a 10-fold volume of water. The THF/water mixture was then sonicated for 2 min using an ultrasound sonicator at 20 W

of the output power (Scientz, China). After evaporation of THF by stirring the mixture in the fume hood for 8 h, the resulting DOD NPs were obtained by filtration through a 0.22-μm filter.

SRS and CARS imaging

In the SRS microscope, a femtosecond laser (OPO, Insight DS^+, Newport) with a fixed 1040 nm Stokes beam and a tunable 680 – 1300 nm pump beam was used. Pulse durations were extended to ~2.3 ps for the pump and ~1.2 ps for the Stokes beam using SF57 glass rods. The intensity of Stokes beam was modulated at a 1/4 of the laser pulse repetition rate (80 MHz) with an electro-optical modulator (EOM). These pulses were aligned spatially and temporally using a dichroic mirror and delay stage, then focused onto the sample in a transmitted light laser scanning microscope (FV1200, Olympus) with an objective lens (UPlanFL 10X, 0.3 NA, Olympus). After passing through the sample, the beams were collected with a high-NA condenser lens (oil immersion, 1.4 NA, Nikon). The Stimulated Raman Loss (SRL) signal was filtered, detected by a photodiode, demodulated with a lock-in amplifier (LIA) (HF2LI, Zurich Instruments), and used to form SRS images. Laser powers at the sample were: pump 20 mW and Stokes 20 mW.

The CARS signal was collected by the objective and filtered by a 791 nm dichroic mirror (FF791-SDi01, Semrock), a 750 nm short pass filter (FF01-750/SP-25, Semrock), and a 710 nm bandpass filter (FF01-710/40-25, Semrock) in sequence. The signals were then detected by a photomultiplier tube integrated in a reflected light laser scanning microscope to yield CARS images. Laser powers at the sample were: pump 20 mW and Stokes 20 mW.

For SRS and CARS imaging of the ALNs, nude mice were injected in the left front paw under anesthesia with DOD NPs in PBS solution containing 150 μg of 1-dodecyne. After 15 min of the

injection, the mice were euthanized and the ALNs were collected. The isolated ALNs were covered with a coverslip for the SRS and CARS Raman imaging. In a separate experiment, the isolated ALNs were covered with a piece of porcine skin slice (~0.4 mm) or mouse skin (~0.75 mm), then with a coverslip for the SRS and CARS Raman imaging.

Multiplexed Raman imaging of tumor and blood vessels

BXZ NPs (20 mg kg^{-1} of BXZ) was i.v. injected into the CT26-Luc orthotopic tumor-bearing BALB/c mice. After 24 h, BBT NPs (40 mg kg^{-1} of BBT) were i.v. injected. Intraoperative multiplexed Raman imaging (inVia Raman microscope, Renishaw, UK) was performed immediately after the injection of BBT NPs. The imaging was conducted in StreamLine high-speed acquisition mode with 785-nm laser, 84.5 mW, 5 × objective, 124.8 μm step size and 0.15 s acquisition time. The characteristic peaks at 894 cm^{-1} for BBT and 1290 cm^{-1} for BXZ were selected for the image processing, respectively.

Supplementary Figs.

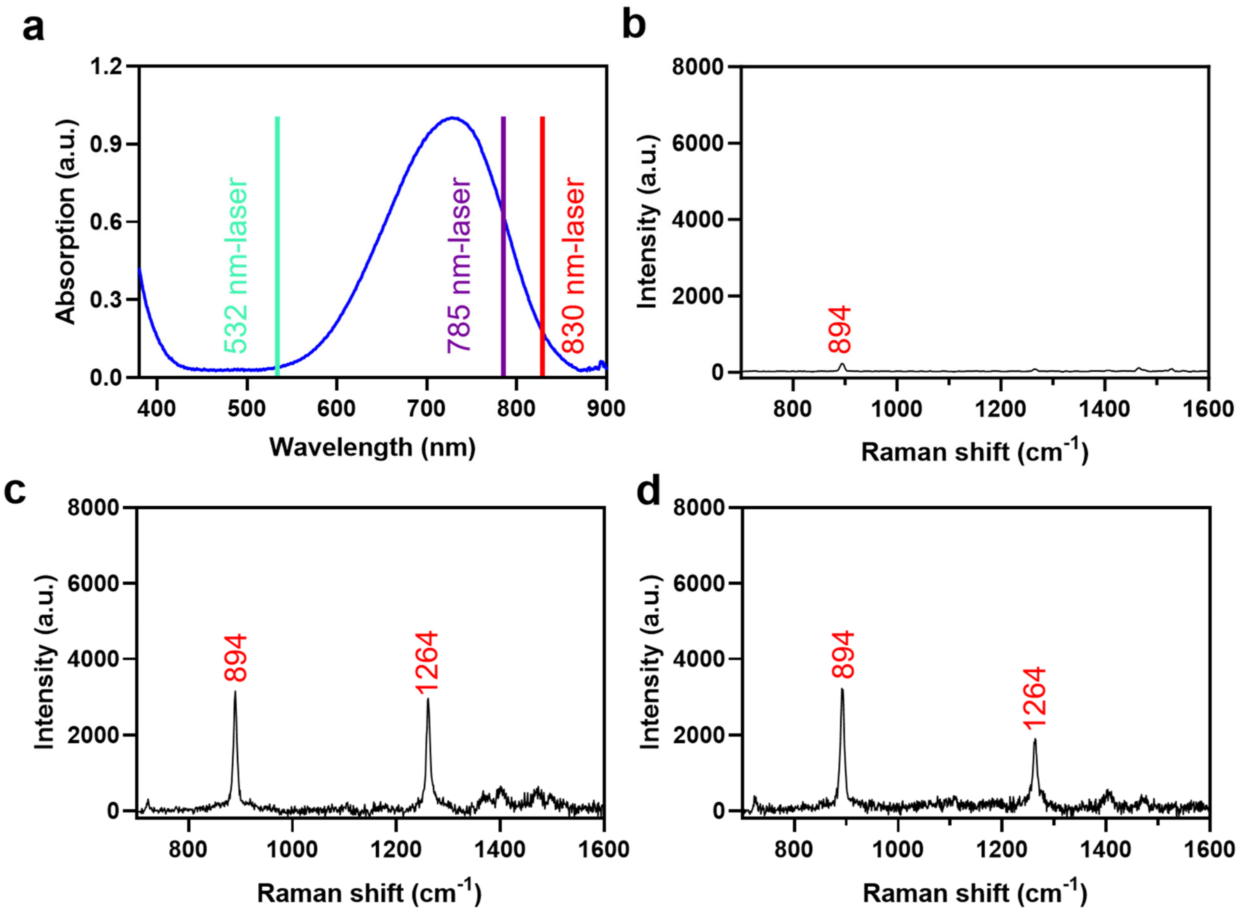

Supplementary Fig. 1 | UV-Vis absorption spectrum and Raman spectra of BBT excited at different wavelength. a, UV-Vis absorption spectrum of BBT (10 μM) in THF. **b-d**, Raman spectra of BBT measured in solid state with 532-nm (**b**), 785-nm (**c**) and 830-nm (**d**) laser excitation, respectively. Raman measurement was carried out with a 20 × objective, acquisition time of 10 s, one time accumulation, and laser powers of 1.6×10^{-2} mW for 532-nm laser, 8.2×10^{-2} mW for 785-nm laser, or 6.1×10^{-2} mW for 830-nm laser.

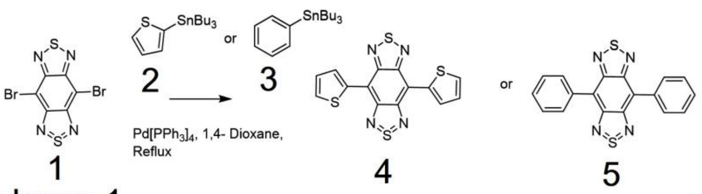

Scheme 1

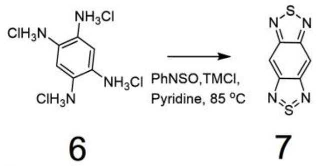

Scheme 2

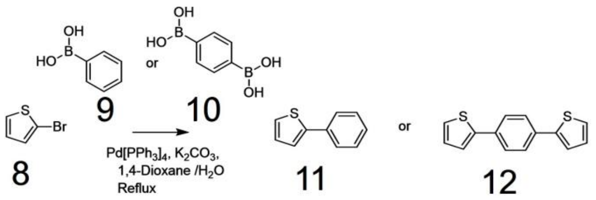

Scheme 3

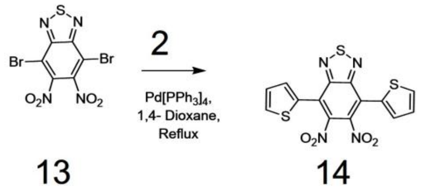

Scheme 4

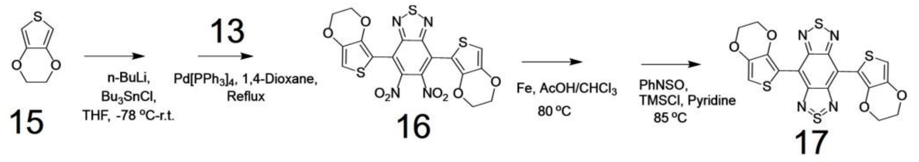

Scheme 5

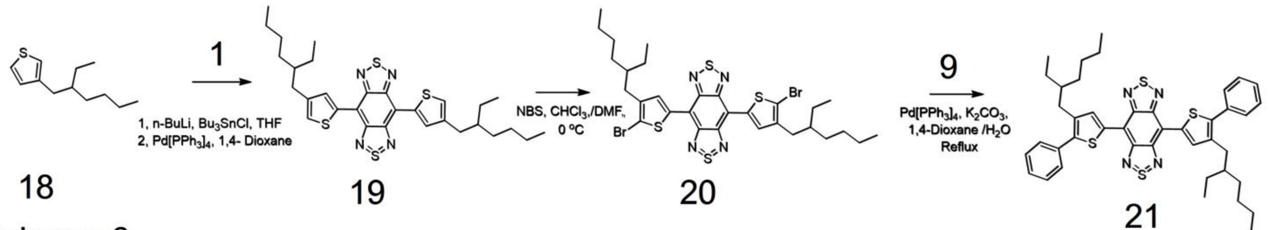

Scheme 6

Supplementary Fig. 2 | Schemes of the synthetic routes of the designed compounds.

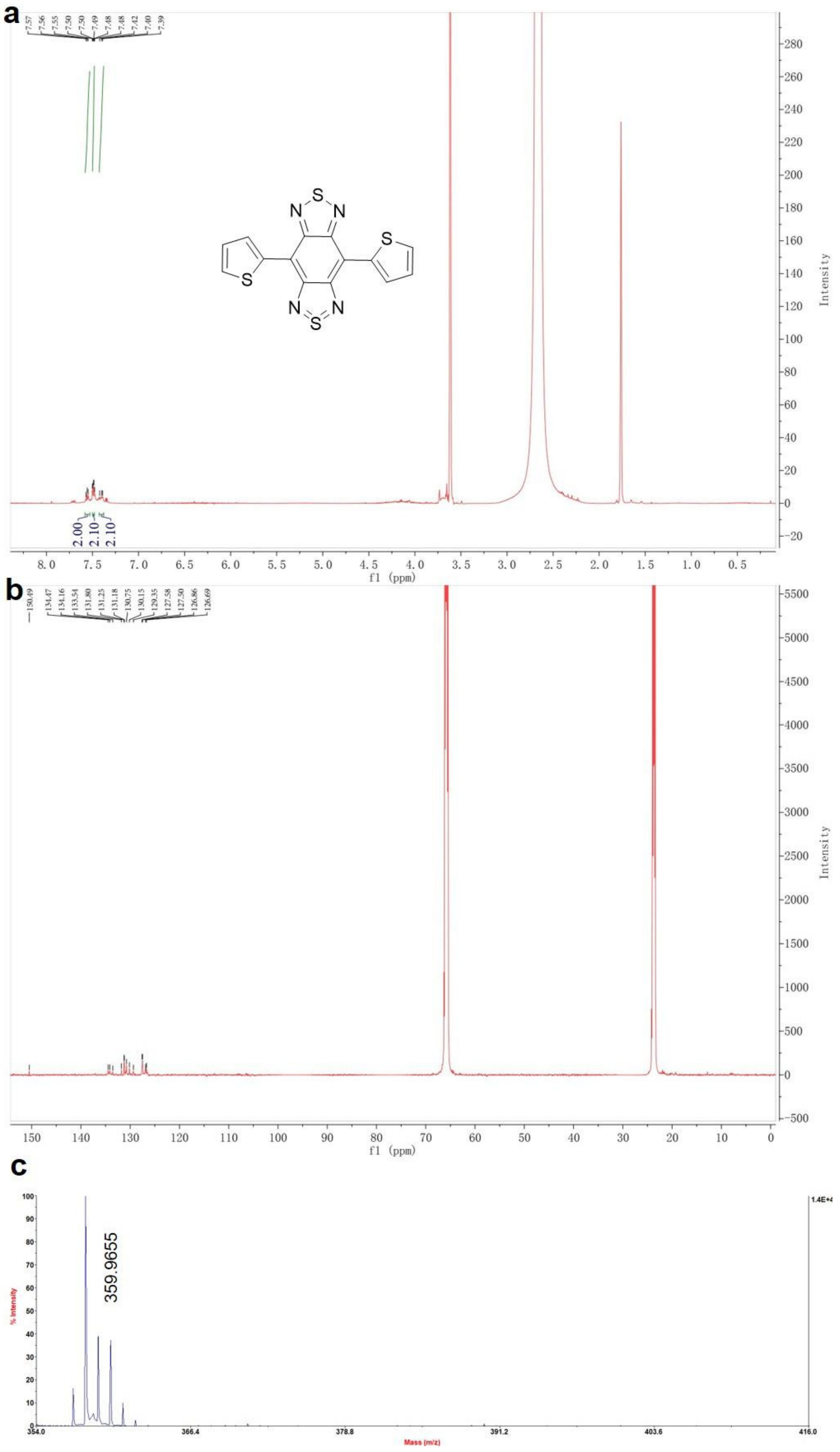

Supplementary Fig. 3 | Characterization of compound 4. **a**, ^{1}H Nuclear magnetic resonance (NMR) spectrum of compound **4** in THF-*d₈*. **b**, ^{13}C NMR spectrum of compound **4** in THF-*d₈*. **c**, MALDI-TOF (MS) spectrum of compound **4**.

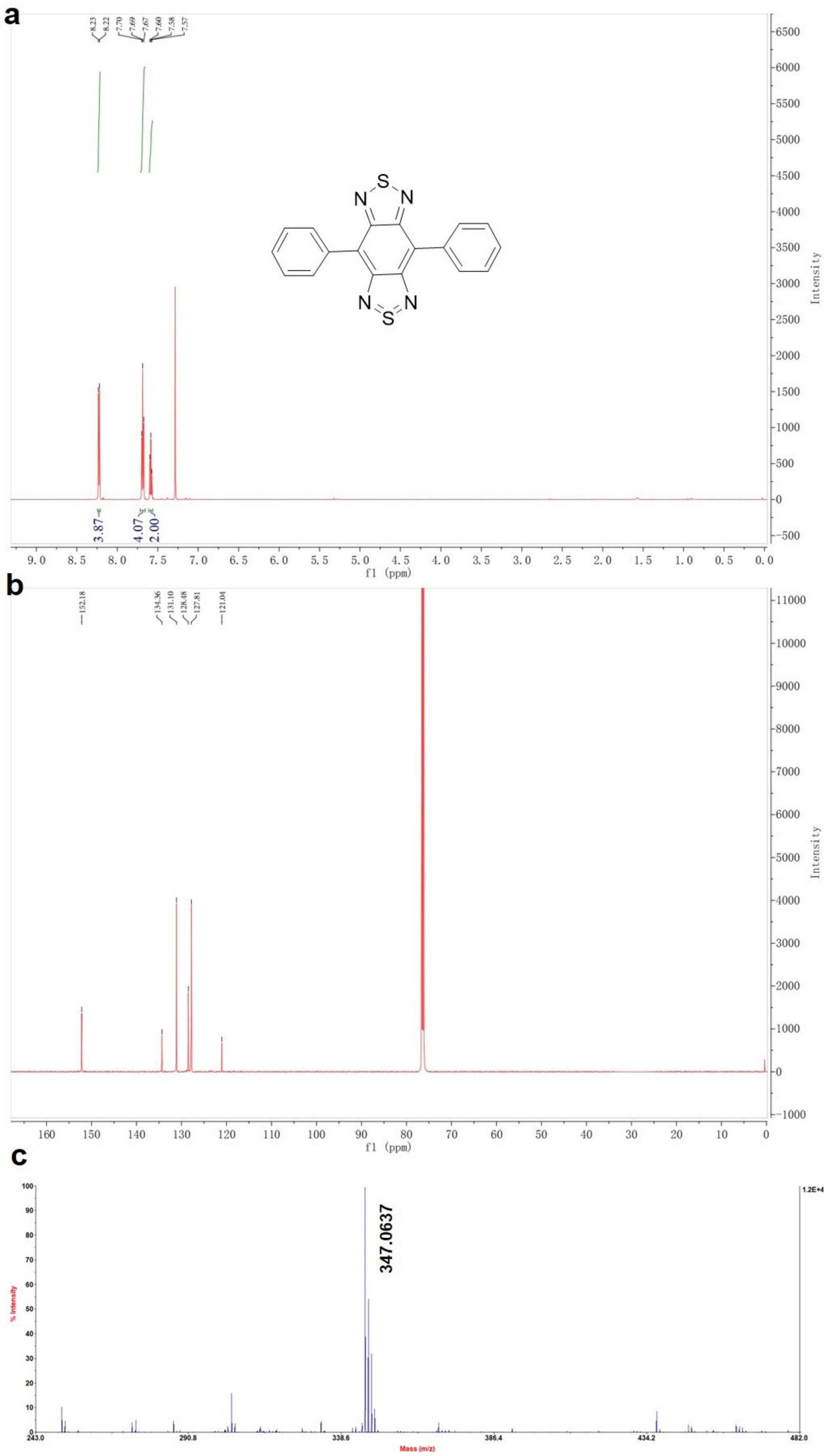

Supplementary Fig. 4 | Characterization of compound 5. a, ^{1}H NMR spectrum of compound **5** in $CDCl_3$. **b**, ^{13}C NMR spectrum of compound **5** in $CDCl_3$. **c**, MALDI-TOF (MS) spectrum of compound **5**.

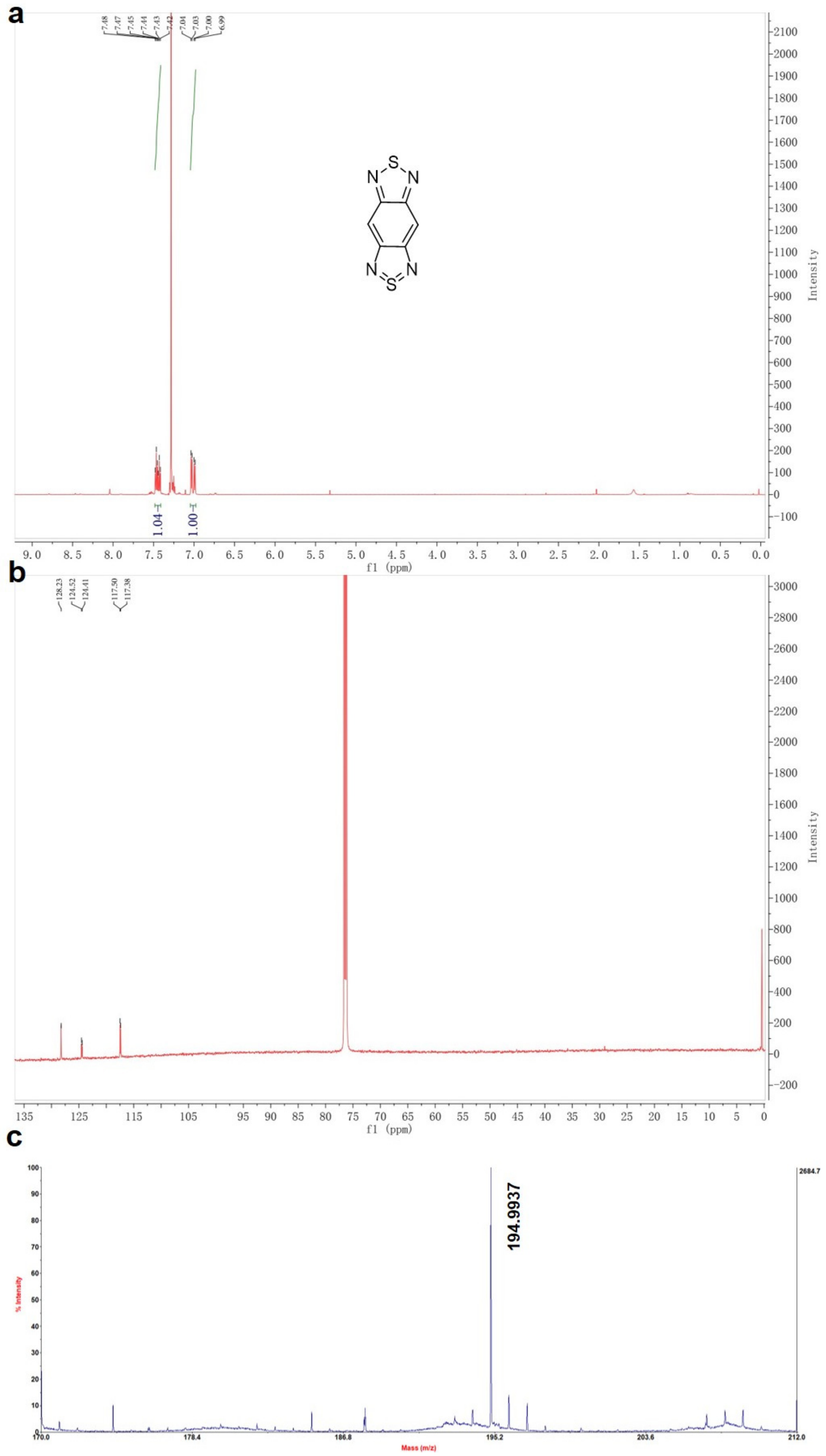

Supplementary Fig. 5 | Characterization of compound 7. a, ^{1}H NMR spectrum of compound **7** in $CDCl_3$. **b**, ^{13}C NMR spectrum of compound **7** in $CDCl_3$. **c**, MALDI-TOF (MS) spectrum of compound **7**.

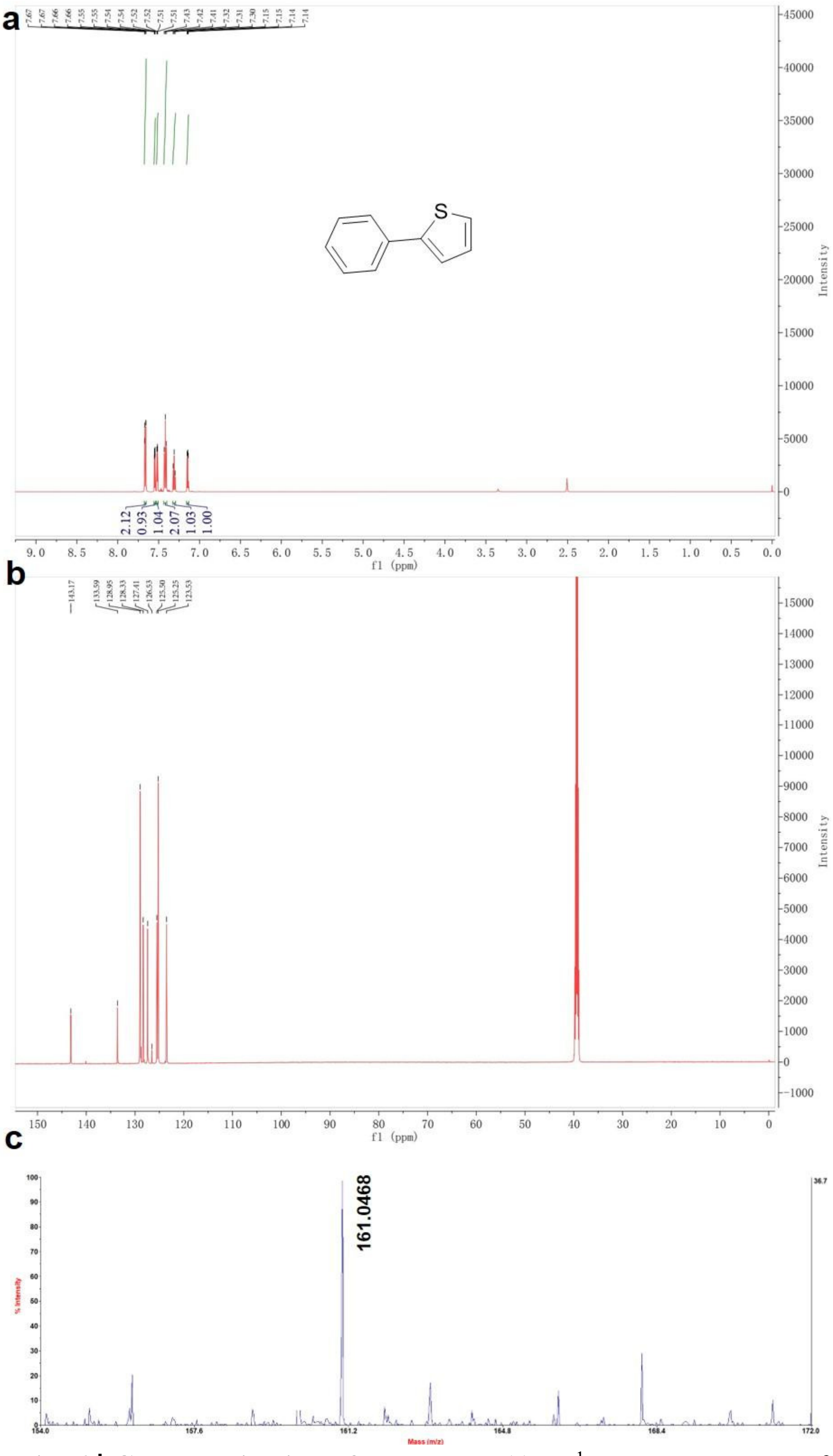

Supplementary Fig. 6 | Characterization of compound 11. a, ^{1}H NMR spectrum of compound **11** in DMSO-d_6. **b**, ^{13}C NMR spectrum of compound **11** in DMSO-d_6. **c**, MALDI-TOF (MS) spectrum of compound **11**.

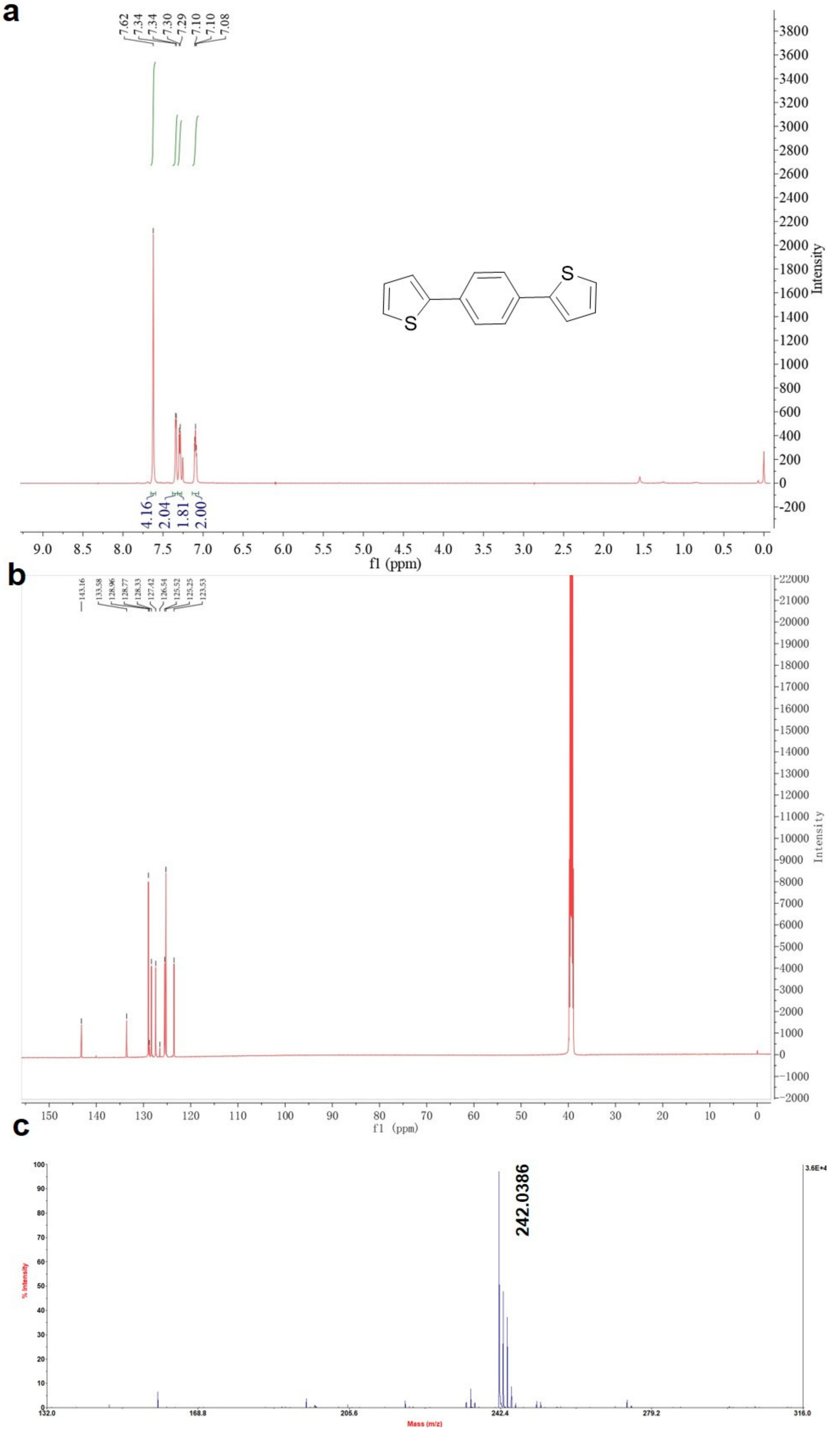

Supplementary Fig. 7 | Characterization of compound 12. a, ^{1}H NMR spectrum of compound **12** in $CDCl_3$. **b**, ^{13}C NMR spectrum of compound **12** in DMSO-d_6. **c**, MALDI-TOF (MS) spectrum of compound **12**.

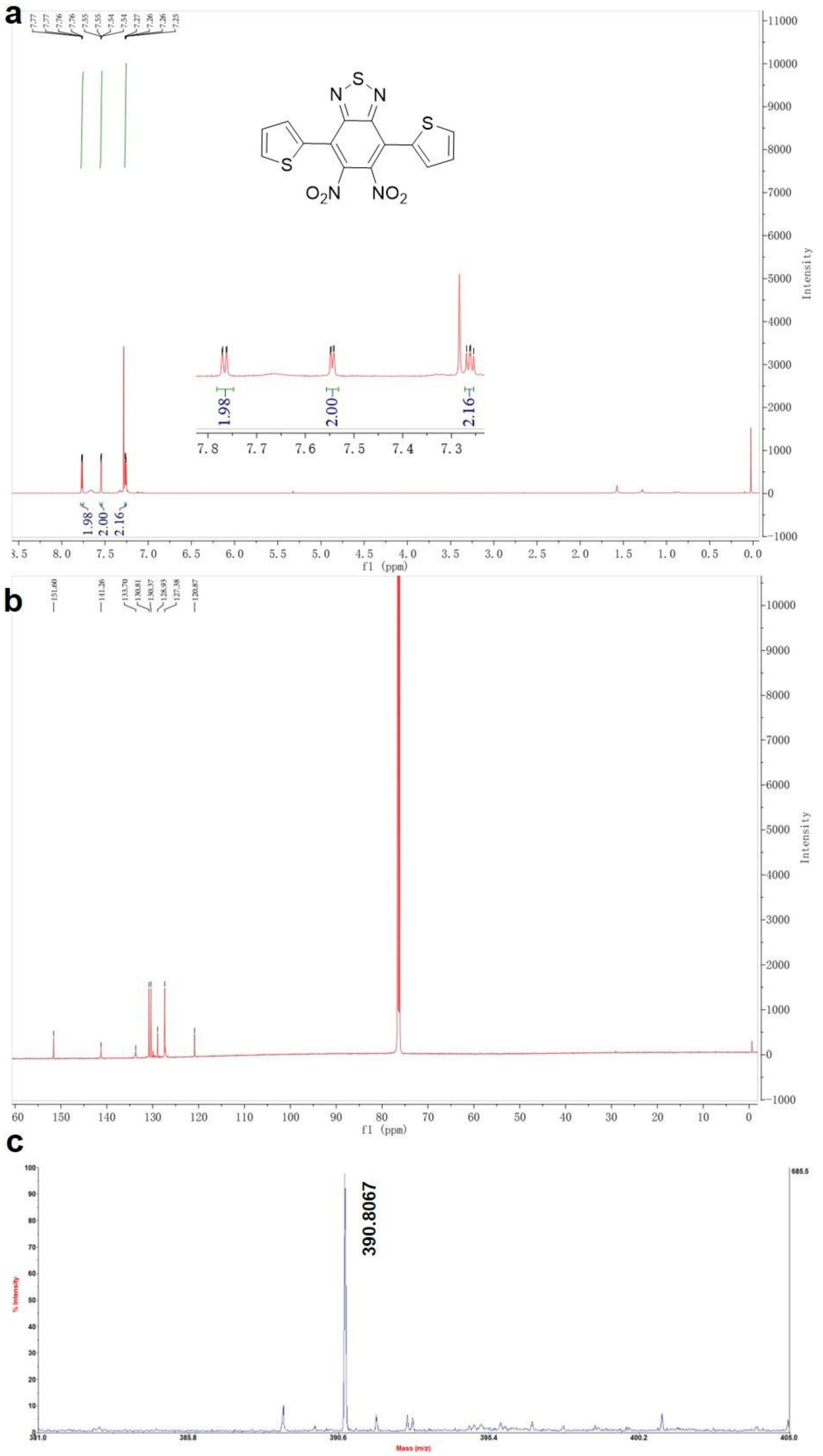

Supplementary Fig. 8 | Characterization of compound 14. a, ^{1}H NMR spectrum of compound **14** in $CDCl_3$. **b**, ^{13}C NMR spectrum of compound **14** in $CDCl_3$. **c**, MALDI-TOF (MS) spectrum of compound **14**.

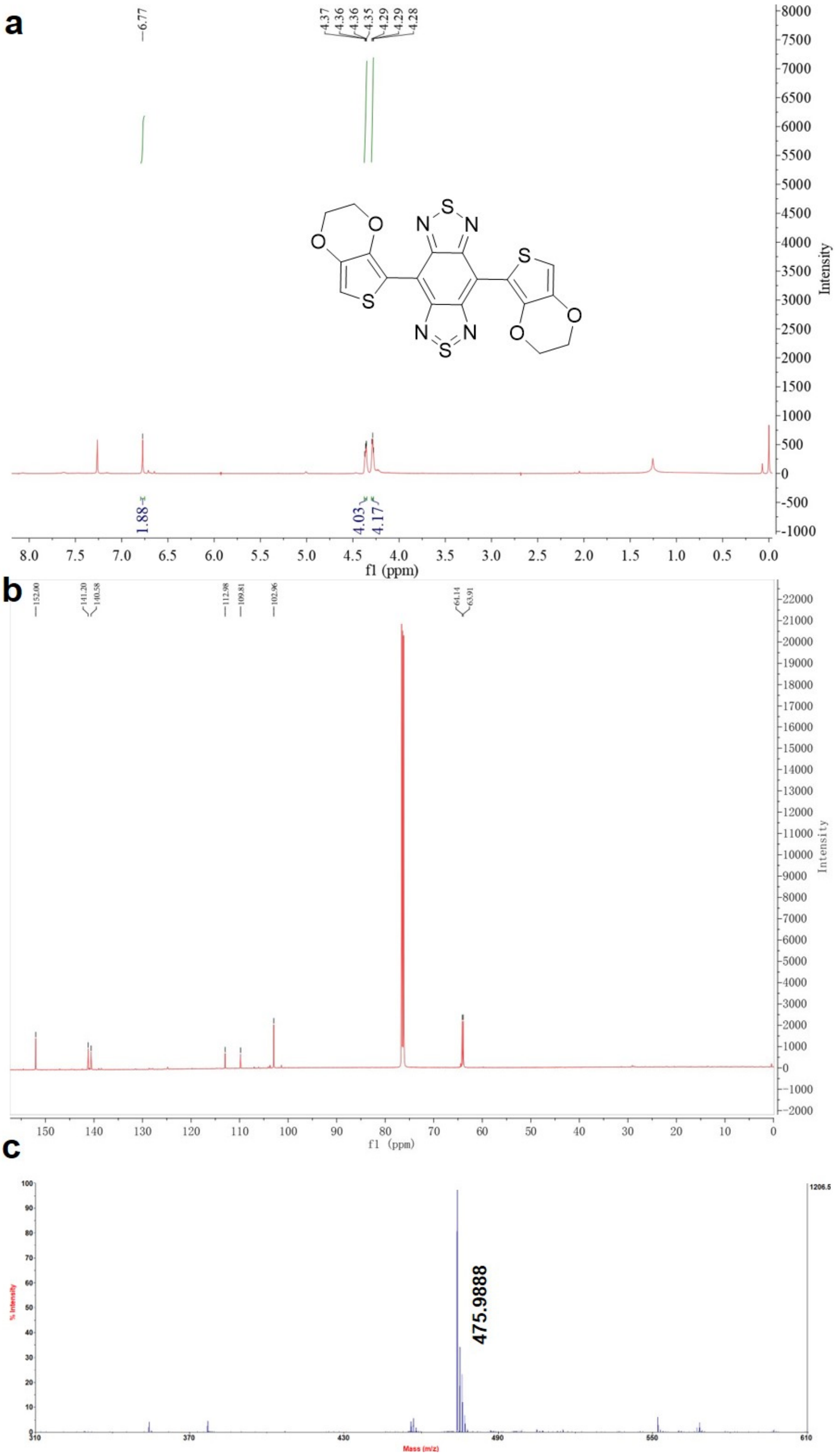

Supplementary Fig. 9 | Characterization of compound 17. a, ^{1}H NMR spectrum of compound **17** in $CDCl_3$. **b**, ^{13}C NMR spectrum of compound **17** in $CDCl_3$. **c**, MALDI-TOF (MS) spectrum of compound **17**.

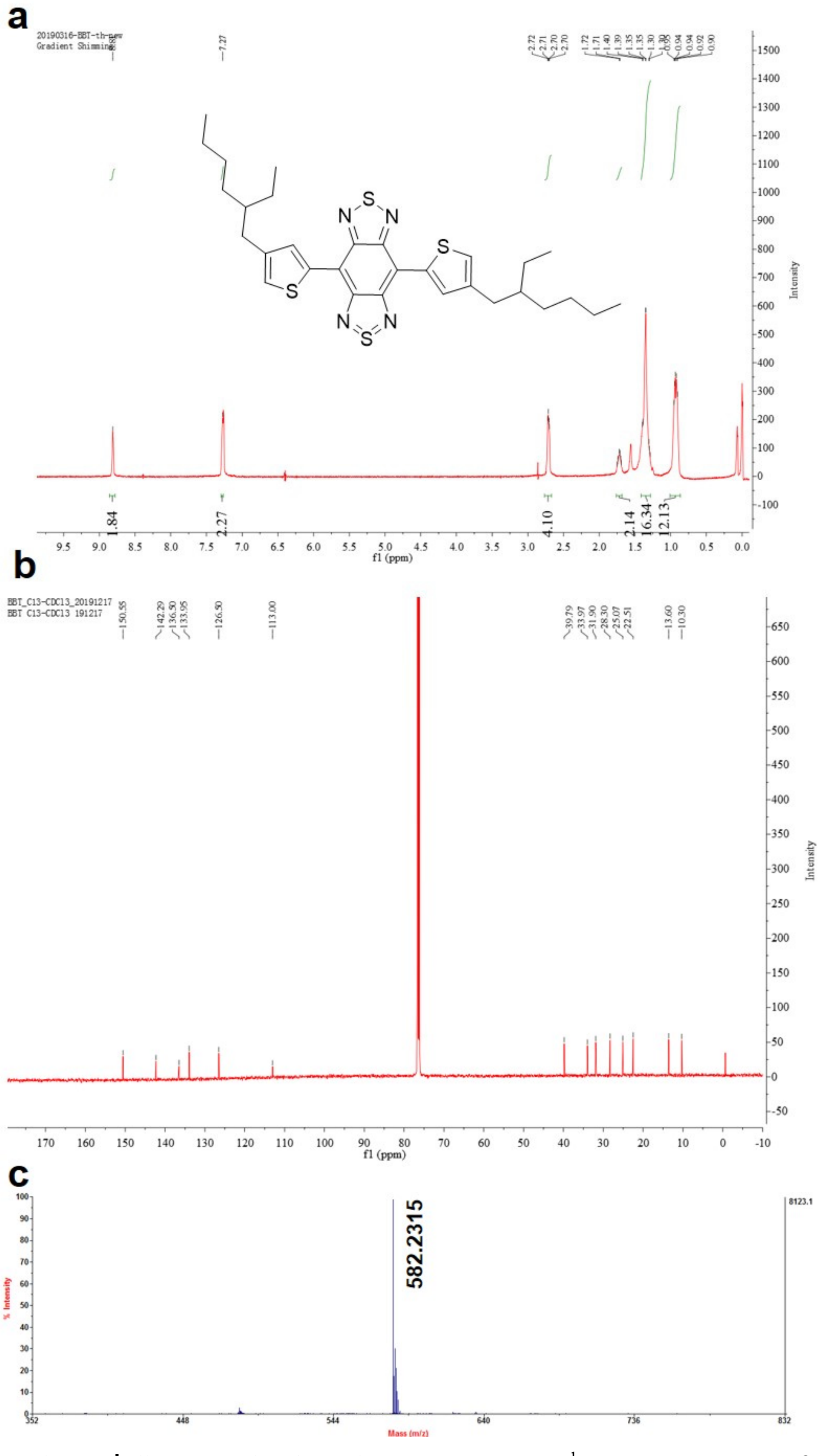

Supplementary Fig. 10 | Characterization of compound 19. a, ^{1}H NMR spectrum of compound **19** (BBT) in $CDCl_3$. **b**, ^{13}C NMR spectrum of BBT in $CDCl_3$. **c**, MALDI-TOF (MS) spectrum of BBT.

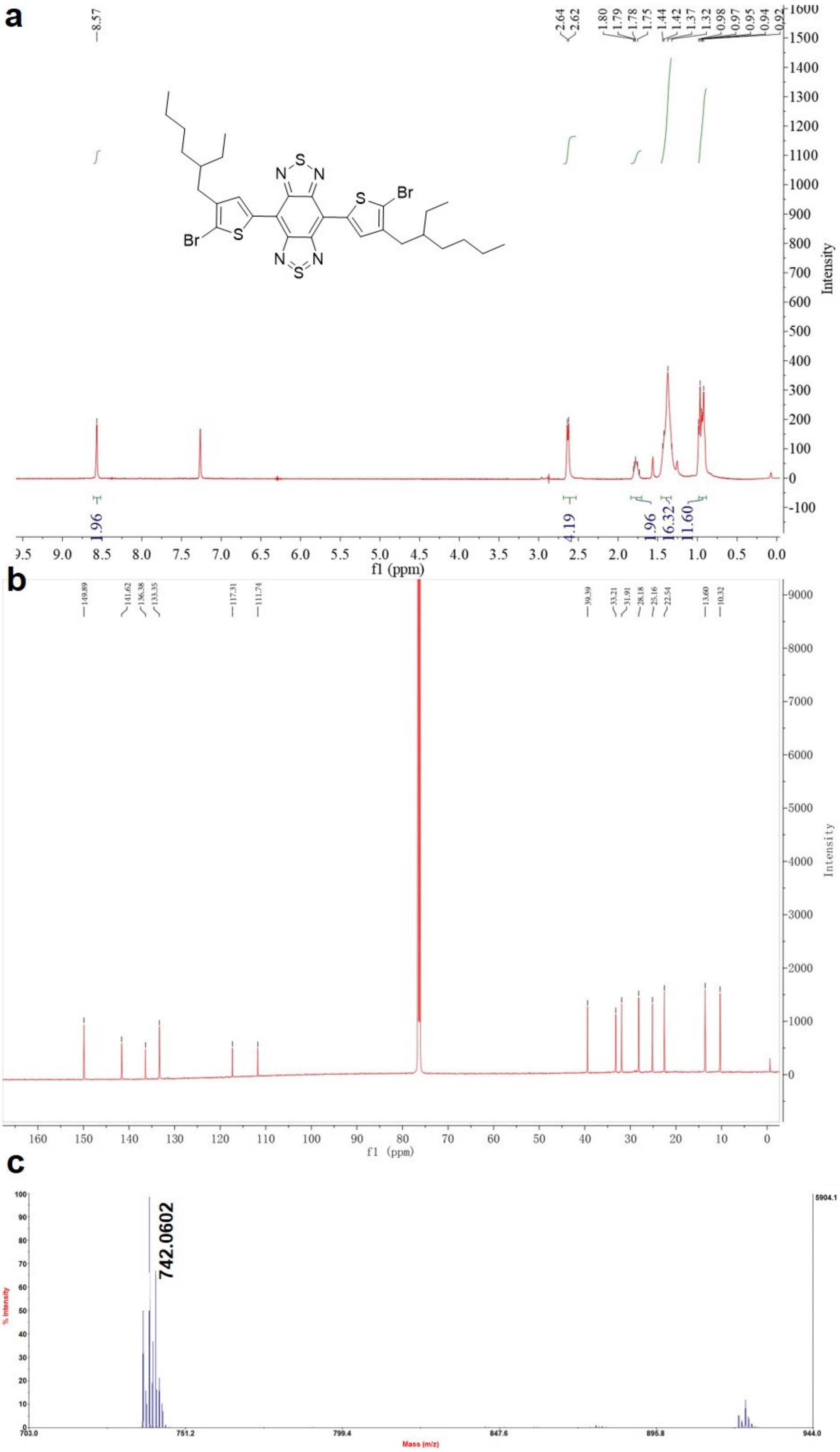

Supplementary Fig. 11 | Characterization of compound 20. a, ^{1}H NMR spectrum of compound **20** in $CDCl_3$. **b**, ^{13}C NMR spectrum of compound **20** in $CDCl_3$. **c**, MALDI-TOF (MS) spectrum of compound **20**.

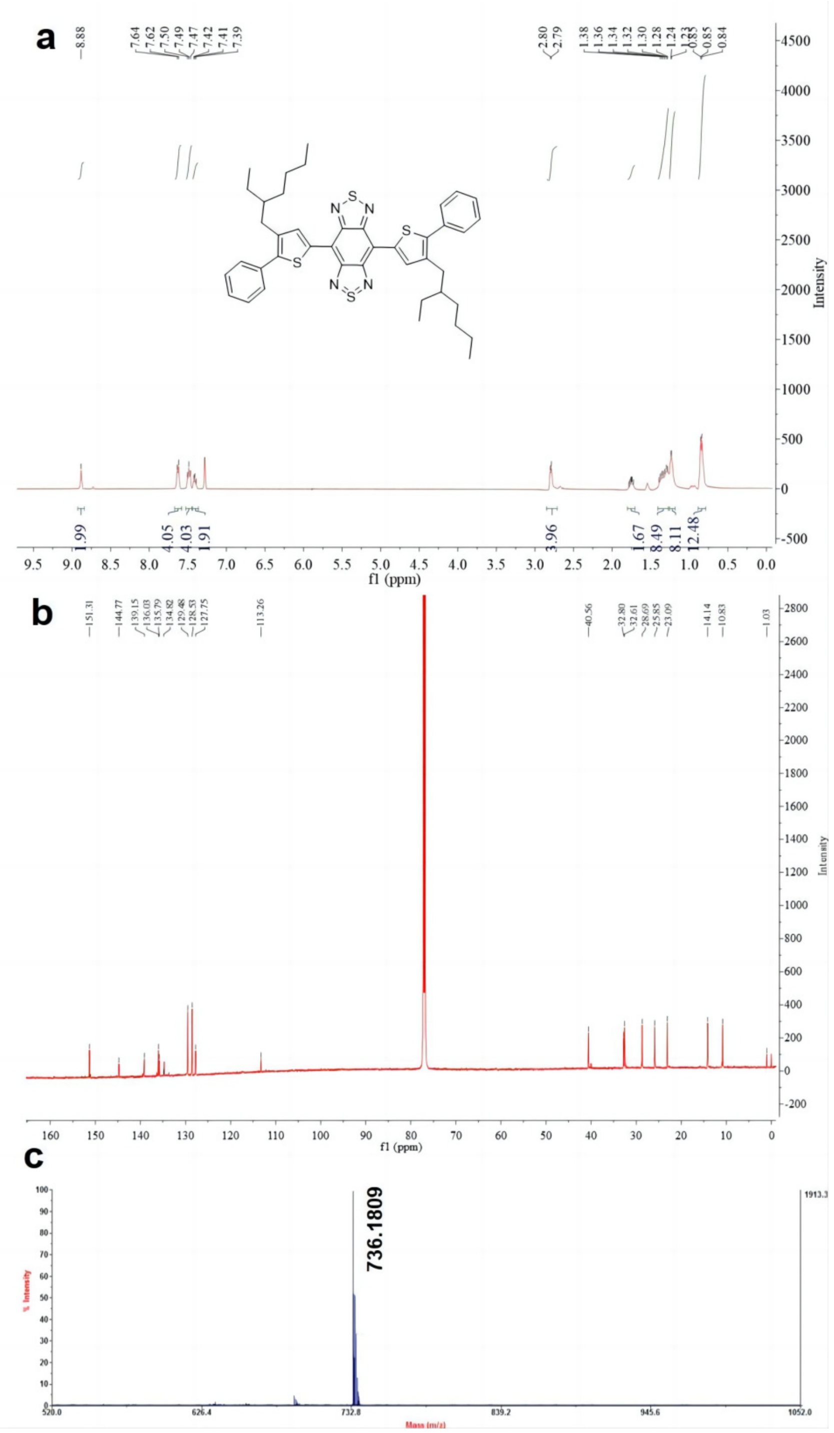

Supplementary Fig. 12 | Characterization of compound 21. a, ^{1}H NMR spectrum of compound **21** in $CDCl_3$. **b**, ^{13}C NMR spectrum of compound **21** in $CDCl_3$. **c**, MALDI-TOF (MS) spectrum of compound **21**.

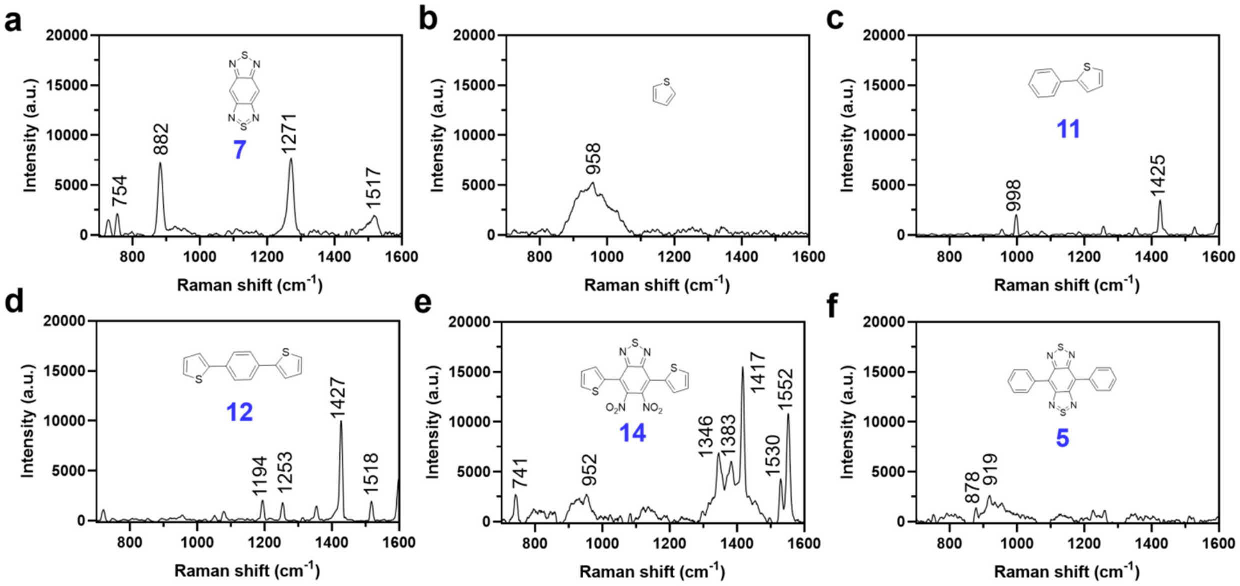

Supplementary Fig. 13 | Chemical structures and Raman spectra of respective molecules. a-f, Chemical structures of various molecules and their respective Raman spectra measured in solid state with 830-nm laser excitation. Raman measurement was carried out with a 20 × objective, a laser power of 6.1 × 10^{-2} mW, 3 times accumulation, and acquisition time of 5 s for compounds **7**, **11**, **12**, **14** and thiophene; acquisition time of 0.2 s for compound **5**.

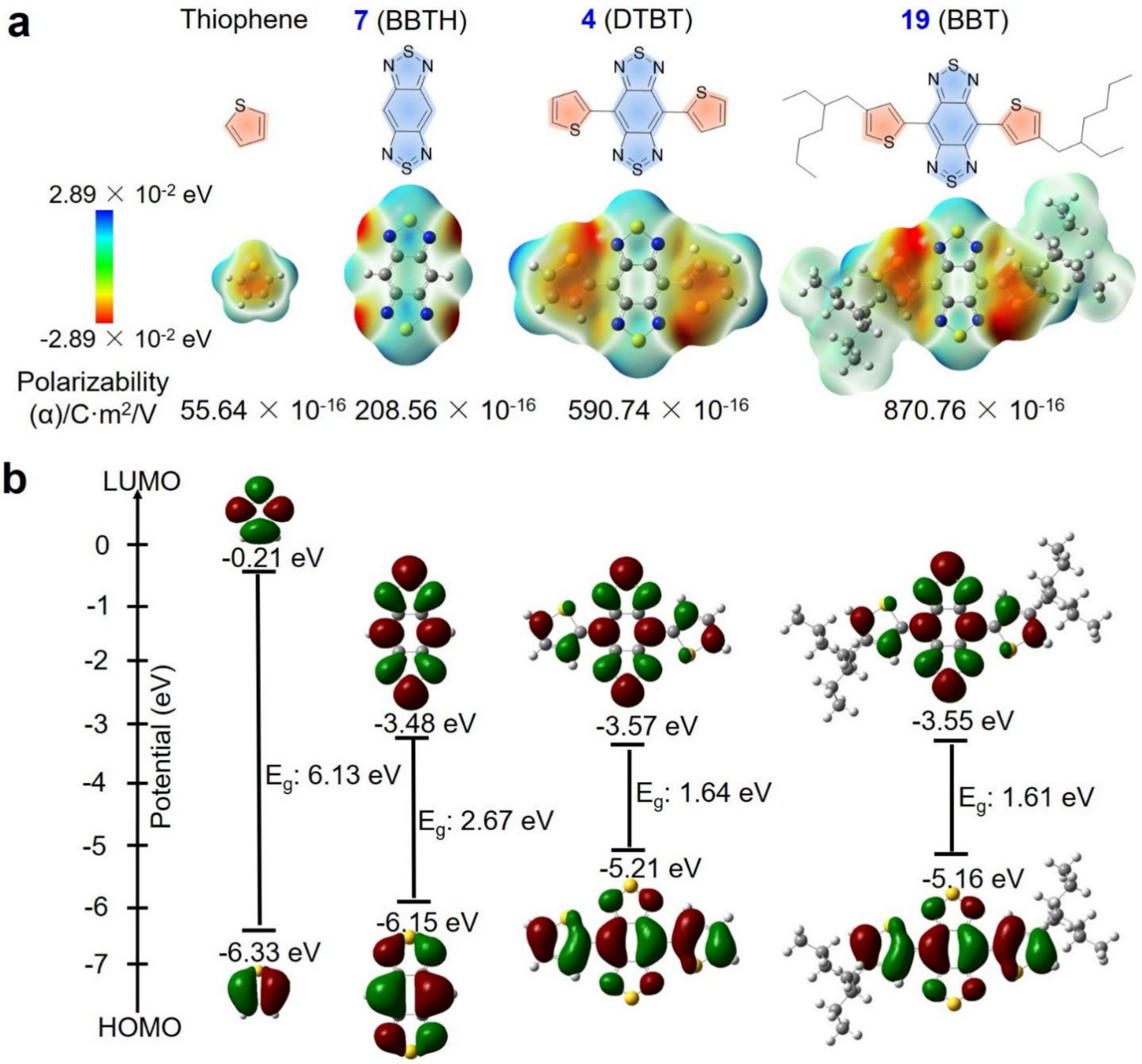

Supplementary Fig. 14 | Increased electron delocalization and reduced energy gap of the DTBT-based D-A-D system. a, Maps of electrostatic potential (ESP) for thiophene, **7** (BBTH), **4** (DTBT) and **19** (BBT), respectively. The ESP maps are plotted using the same color range from red (-2.89 × 10^{-2} eV, negative) to blue (2.89 × 10^{-2} eV, positive). The red and blue colors in the ESP maps represent the lowest and highest electrostatic potential energy values, respectively. The polarizability values are calculated by Gaussian 09/B3LYP/6-31G(d). **b**, Highest occupied molecular orbitals (HOMOs) and lowest unoccupied molecular orbitals (LUMOs) distributions of the compounds. The optimized geometries of the HOMO and LUMO with the energy gap at S_0 are calculated by DFT (Gaussian 09/B3LYP/6-31G(d)).

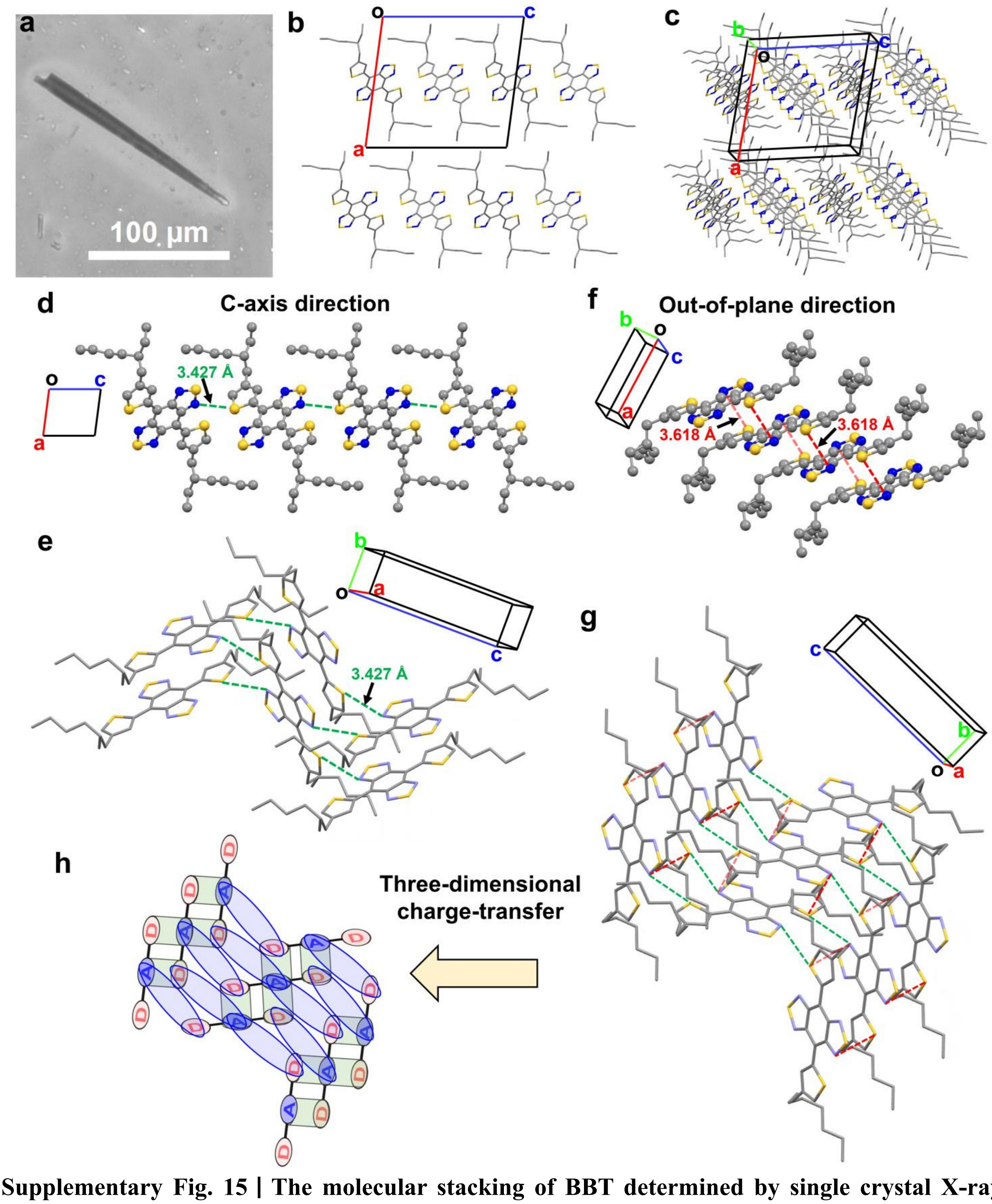

Supplementary Fig. 15 | The molecular stacking of BBT determined by single crystal X-ray diffraction, with close intermolecular distances allowing for a three-dimensional charge-transfer between the neighboring molecules. **a**, An optical micrograph of the BBT single crystal. **b**,**c**, The whole view of the packing pattern of BBT in the crystal. **d**,**e**, A backbone-to-backbone stacking of BBT molecules along c-axis to form a 3.427 Å of gap (green dotted lines) between the sulfur atom of the thiophene in one BBT molecule and the nitrogen atom of the benzobisthiadiazole in the neighboring BBT molecule. **f**, The BBT molecules were piled up in the out-of-plane direction to

constitute a continuous π-π stacking, with a 3.618 Å of distance (red dotted lines) between the sulfur atom of the thiophene in one molecular plane and the nitrogen atom of the benzobisthiadiazole in the other molecular plane. **g**, The arrangement of BBT in the single crystal structure showed the intermolecular distance in both the c-axis direction (3.427 Å, green dotted lines) and the out-of-plane direction (3.618 Å, red dotted lines). **h**, Schematic diagram of three-dimensional charge-transfer interactions of BBT. The stacking of the BBT-based planar D-A-D molecules illustrates that one acceptor could totally receive electrons from six donors (D_2—A•••D_4), including two intramolecular and four intermolecular donors. Meanwhile, one donor could donate electrons to three acceptors (A—D•••A_2), including one intramolecular and two intermolecular acceptors. Green cylinders, intermolecular D•••A change-transfer in the out-of-plane direction. Blue ellipses, intermolecular D•••A change-transfer along c-axis.

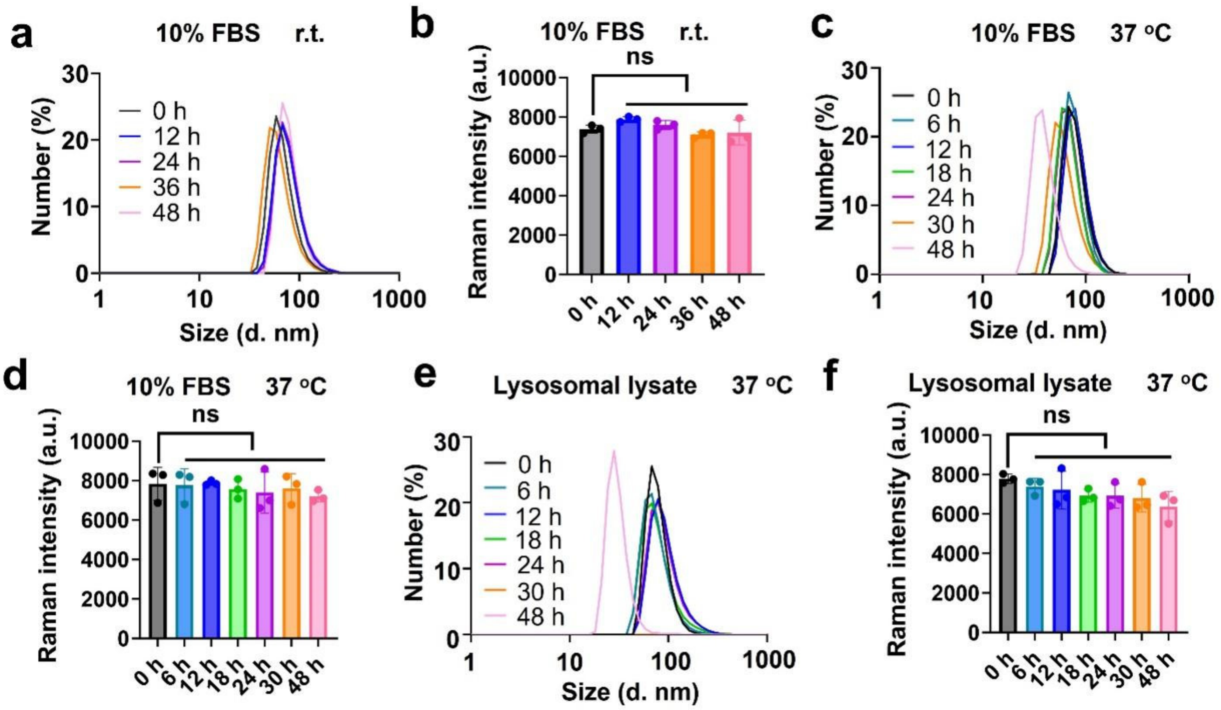

Supplementary Fig. 16 | *In vitro* stability of BBT NPs. a,**b**, Size distribution (**a**) and Raman signal (894 cm^{-1}) (**b**) of BBT NPs following the incubation with 10% FBS for different times at room temperature (r.t.). **c**,**d**, Size distribution (**c**) and Raman signal (894 cm^{-1}) (**d**) of BBT NPs following the incubation with 10% FBS for different times at 37 ºC. **e**,**f**, Size distribution (**e**) and Raman signal (894 cm^{-1}) (**f**) of BBT NPs following the incubation with the lysosomal lysate for different times at 37 ºC. Data shown is representative of $n = 3$ independent replicates of experiments with similar results (**a,c,e**). $n = 3$ (**b,d,f**) biologically independent samples. Data are presented as the mean ± s.d. (**b**,**d**,**f**), and one-way ANOVA with Dunnett's multiple comparisons test was used in (**b,d,f**). ns denotes no significant difference ($P > 0.05$).

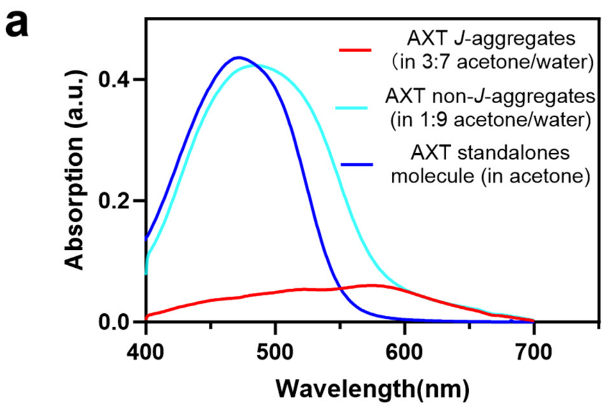

b

AXT in different states	Raman scattering cross-section (x 10^{-25} cm^2 per molecule, 532 nm)	Raman scattering cross-section (x 10^{-25} cm^2 per molecule, 785 nm)	Raman scattering cross-section (x 10^{-25} cm^2 per molecule, 830 nm)
J-aggregates (in 3:7 acetone/water)	10.08 ± 0.38	3.81 ± 0.06	0.13 ± 0.01
non-*J*-aggregates (in 1:9 acetone/water)	4.06 ± 1.42	2.01 ± 0.08	0.11 ± 0.01
Standalone molecules (in acetone)	4.41 ± 0.23	2.70 ± 0.04	0.17 ± 0.05

Supplementary Fig. 17 | Absorption spectra and Raman scattering cross-section of AXT *J*-aggregates. a, UV-visible spectra of astaxanthin (AXT, 10 μM) *J*-aggregates in acetone/water (3:7, v/v) mixture, non-*J*-aggregates in acetone/water (1:9, v/v) mixture, and standalone molecules in acetone, respectively. **b**, Raman scattering cross-section of AXT in difference states in (**a**). The cross-section per AXT molecule was calculated by measuring the Raman peak at 1515 cm^{-1} for *J*-aggregates and 1520 cm^{-1} for non-*J*-aggregates and monomer under the excitation at the wavelengths of 532 nm, 785 nm and 830 nm, respectively. Raman measurement was carried out with a 5 × objective, acquisition time of 1 s, one time accumulation, and laser powers of 15.8 mW for 532-nm laser, 84.5 mW for 785-nm laser or 62.6 mW for 830-nm laser. $n = 3$ independent samples (**b**). Data are presented as the mean ± s.d. (**b**).

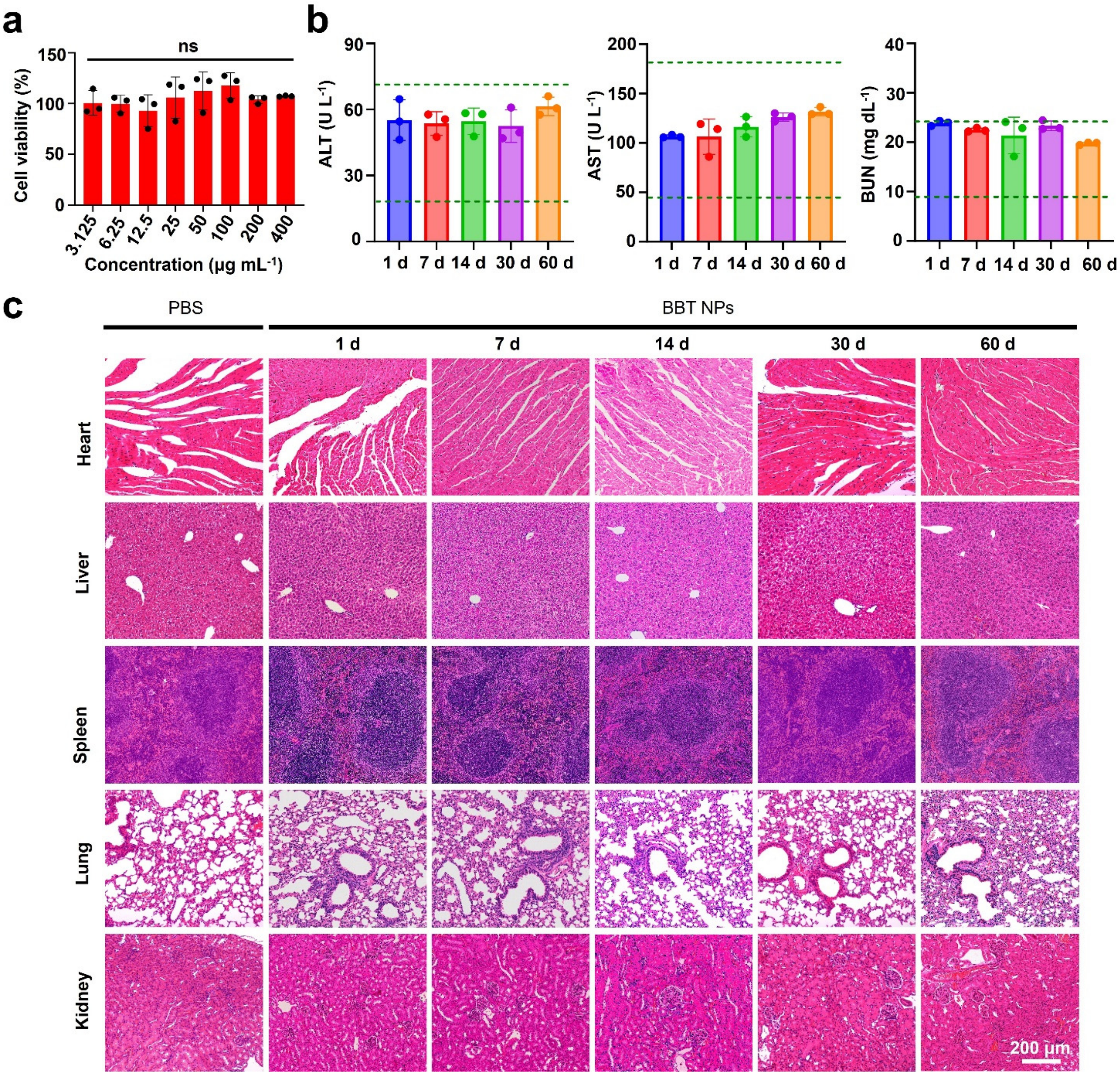

Supplementary Fig. 18 | Biosafety analysis of BBT NPs *in vitro* and *in vivo*. a, Cell viability of NIH 3T3 cells after incubation with BBT NPs at various concentrations for 24 h. **b,** Serum biochemistry including alanine aminotransferase (ALT) and aspartate aminotransferase (AST) and blood urea nitrogen (BUN) levels of ICR mice at 1, 7, 14, 30 and 60 d after intravenous injection of BBT NPs (40 mg kg^{-1}). Reference ranges of hematology data of healthy ICR mice were obtained from Charles River Laboratories: (http://www.criver.com/), dotted lines were used to indicate upper and lower limits. **c,** Histologic analysis of major organs of healthy ICR mice at 1, 7, 14, 30 or 60 d post-injection of BBT NPs (40 mg kg^{-1} of BBT) or PBS following the H&E staining. Bars, 200 μm. Image shown is representative of $n = 3$ independent replicates of experiments with similar results (**c**). $n = 3$ (**a,b**) biologically independent samples. Data are presented as the mean ± s.d. (**a,b**), and one-way ANOVA with Tukey's multiple comparisons test was used in (**a**). ns denotes no significant difference ($P > 0.05$).

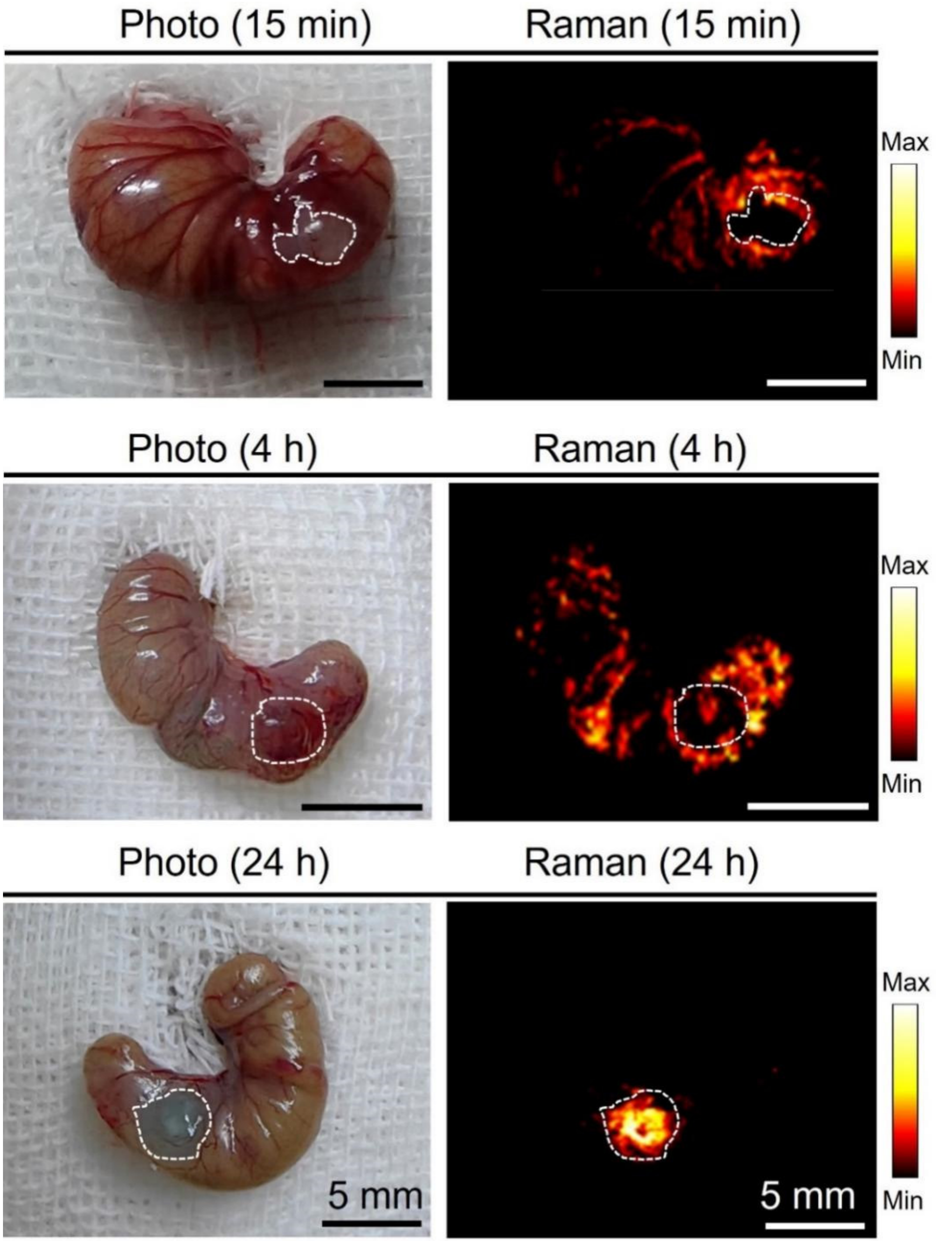

Supplementary Fig. 19 | Intraoperative Raman imaging of the orthotopic mouse colon tumor by SICTERS following the injection of BBT NPs at different time points. Left, photographs; Right, live Raman imaging (894 cm^{-1}) of CT26-Luc tumor lesions of mice following the i.v. injection of BBT NPs (40 mg kg^{-1} of BBT) for 15 min, 4 h and 24 h, respectively. Circle, tumor. Bars, 5 mm. Raman measurement was carried out with a 5 × objective, 830-nm laser excitation, a laser power of 31.3 mW, acquisition time of 0.3 s and one time accumulation.

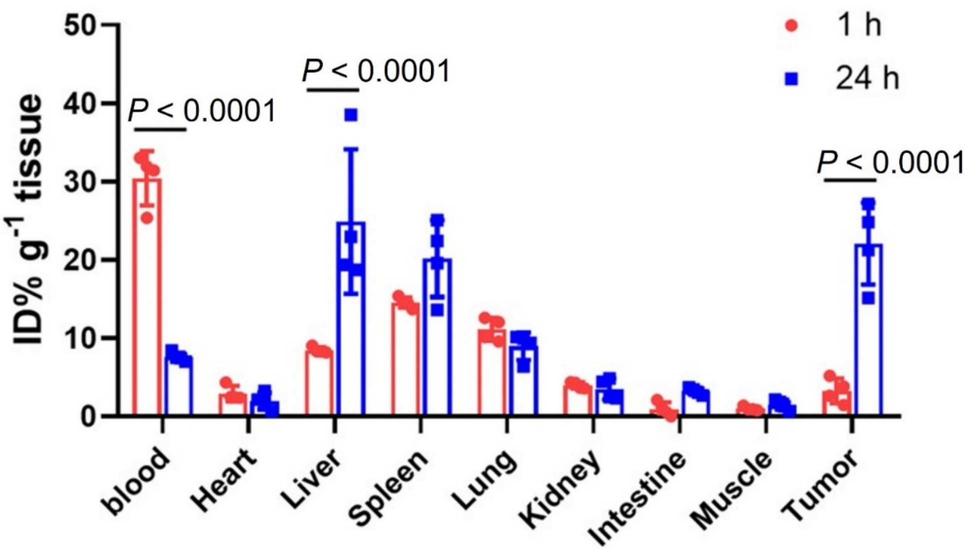

Supplementary Fig. 20 | Biodistribution of BBT in CT26-Luc orthotopic tumor-bearing mice at 1 h or 24 h after i.v. injection of BBT NPs at 40 mg kg^{-1} of BBT. The concentration of BBT in each tissue was calculated by measuring the fluorescence intensity of BBT extracted by toluene (Ex = 830 nm, Em = 1000 nm). ID% g^{-1} tissue, percentage of injection dose per g tissue. n = 4 biologically independent samples. Data are presented as the mean ± s.d., and two-way ANOVA with Sidak's post hoc test was used, with P values indicated on the graphs. $P < 0.05$ was considered statistically significant.

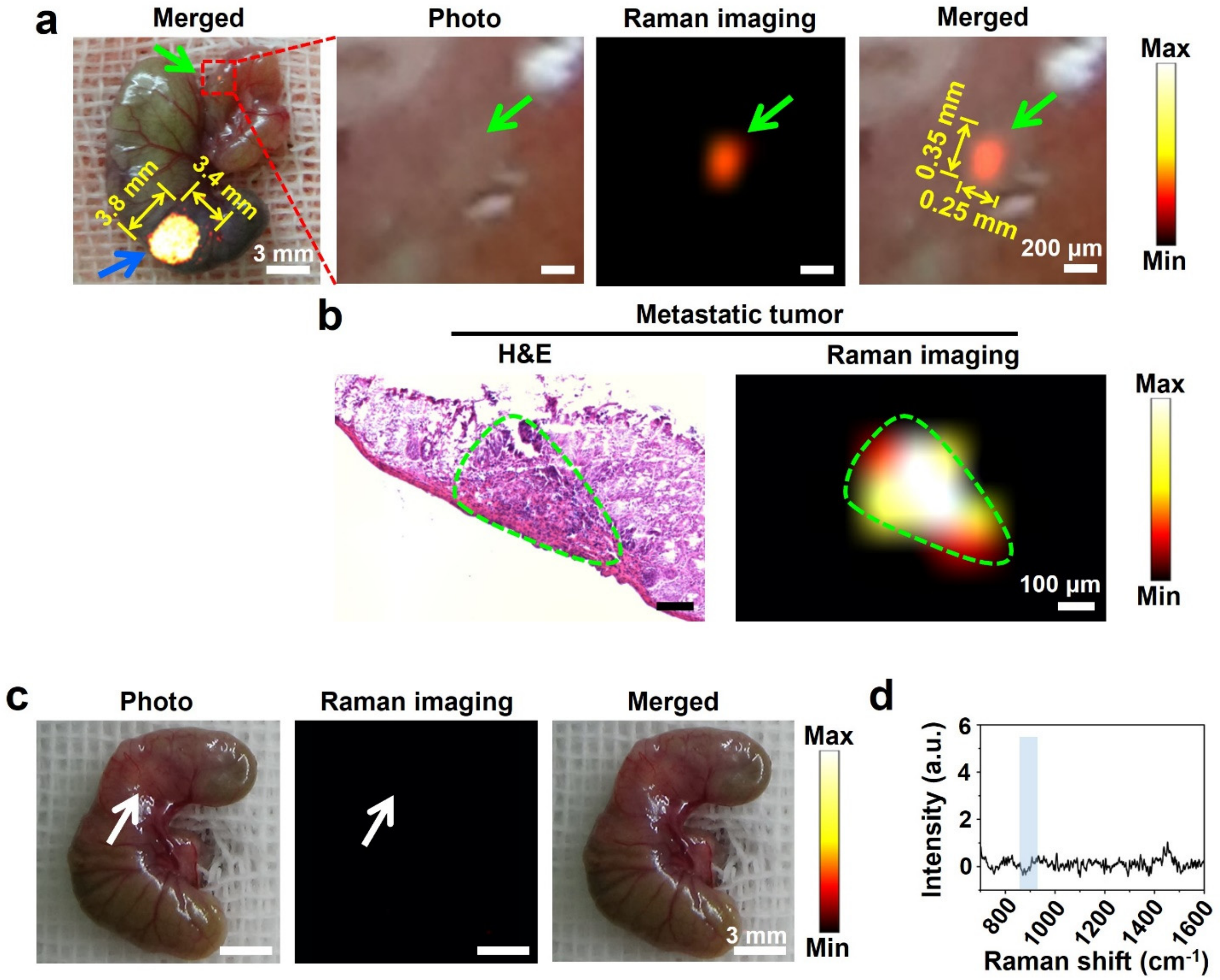

Supplementary Fig. 21 | Intraoperative Raman imaging (894 cm^{-1}) of orthotopic CT26-Luc colon tumor in mice following i.v. injection of BBT NPs (40 mg kg^{-1} of BBT) or PBS. a, Live Raman imaging of primary (blue arrow) and metastatic (green arrows) tumor lesions. **b**, H&E staining (8-μm thickness) and Raman imaging (50-μm thickness) of the two adjacent sections of the metastatic tumor indicated by the green arrows in (**a**), respectively. Green circles, tumor region. Image shown is representative of $n = 3$ independent replicates of experiments with similar results. **c**, The intraoperative Raman imaging of tumor in mice with PBS. Arrow, primary tumor. **d**, Raman spectrum of the position indicated by the arrow in (**c**), without showing peak at 894 cm^{-1} (blue column). Raman measurement was carried out with a 5 × objective, 830-nm laser excitation, laser powers of 31.3 mW in (**a**,**c**) or 62.6 mW in (**b**), acquisition time of 0.3 s and one time accumulation.

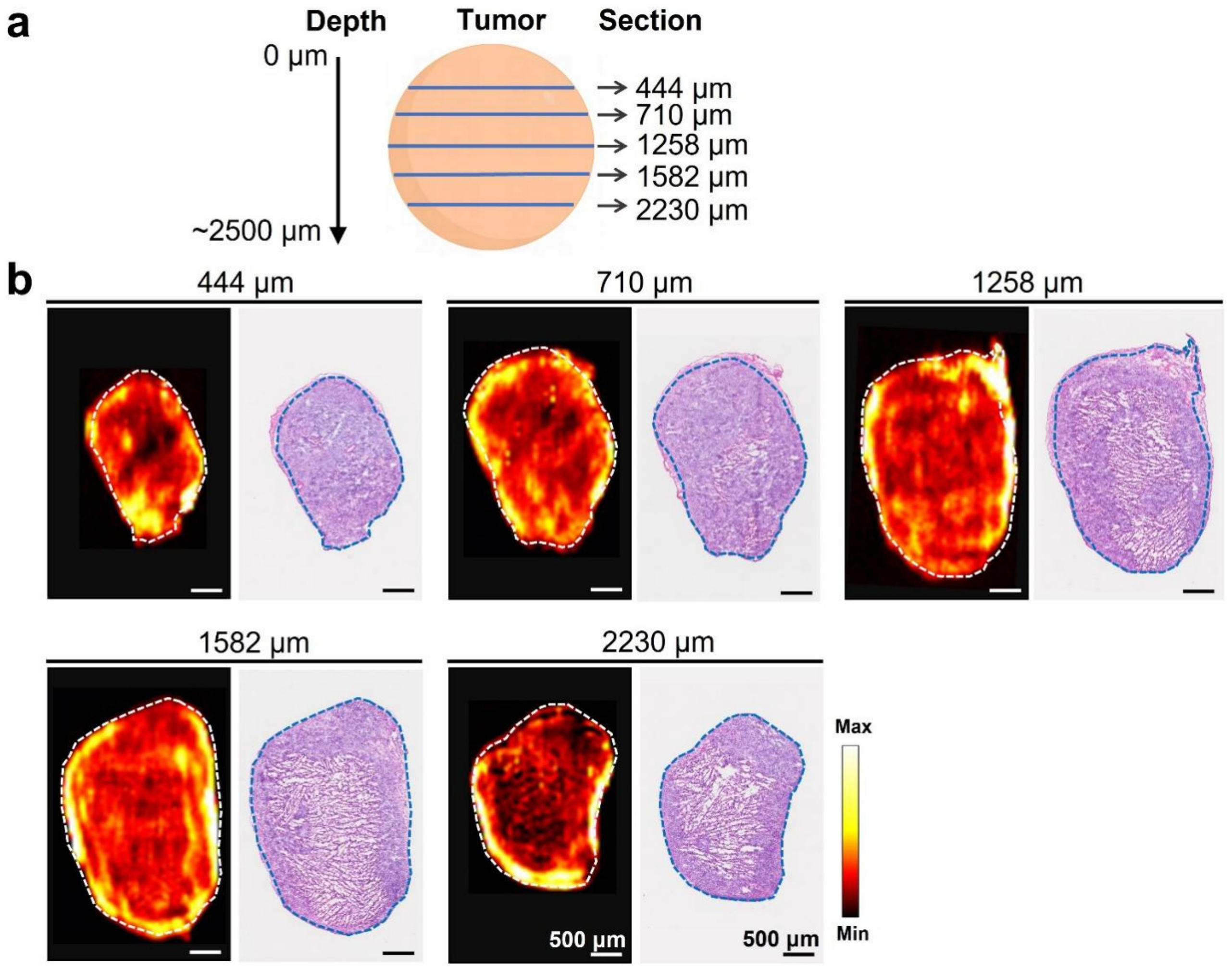

Supplementary Fig. 22 | Tumor tissue penetration of BBT NPs. a, Schematic diagram of sections of tumor with ~2.5 mm in length. Blues lines, tumor cross-section at different depths. **b**, Raman images and H&E-stained micrographs of tumor sections at different depths, with Raman signals uniformly distributed across the cross-section at 1258 μm central depth. Left, Raman images (894 cm^{-1}). Right, the H&E-stained micrographs. Bars, 500 μm. Raman measurement was carried out with a 5 × objective, 830-nm laser excitation, a laser power of 62.6 mW, acquisition time of 1 s and one time accumulation. Image shown is representative of $n = 3$ independent replicates of experiments with similar results.

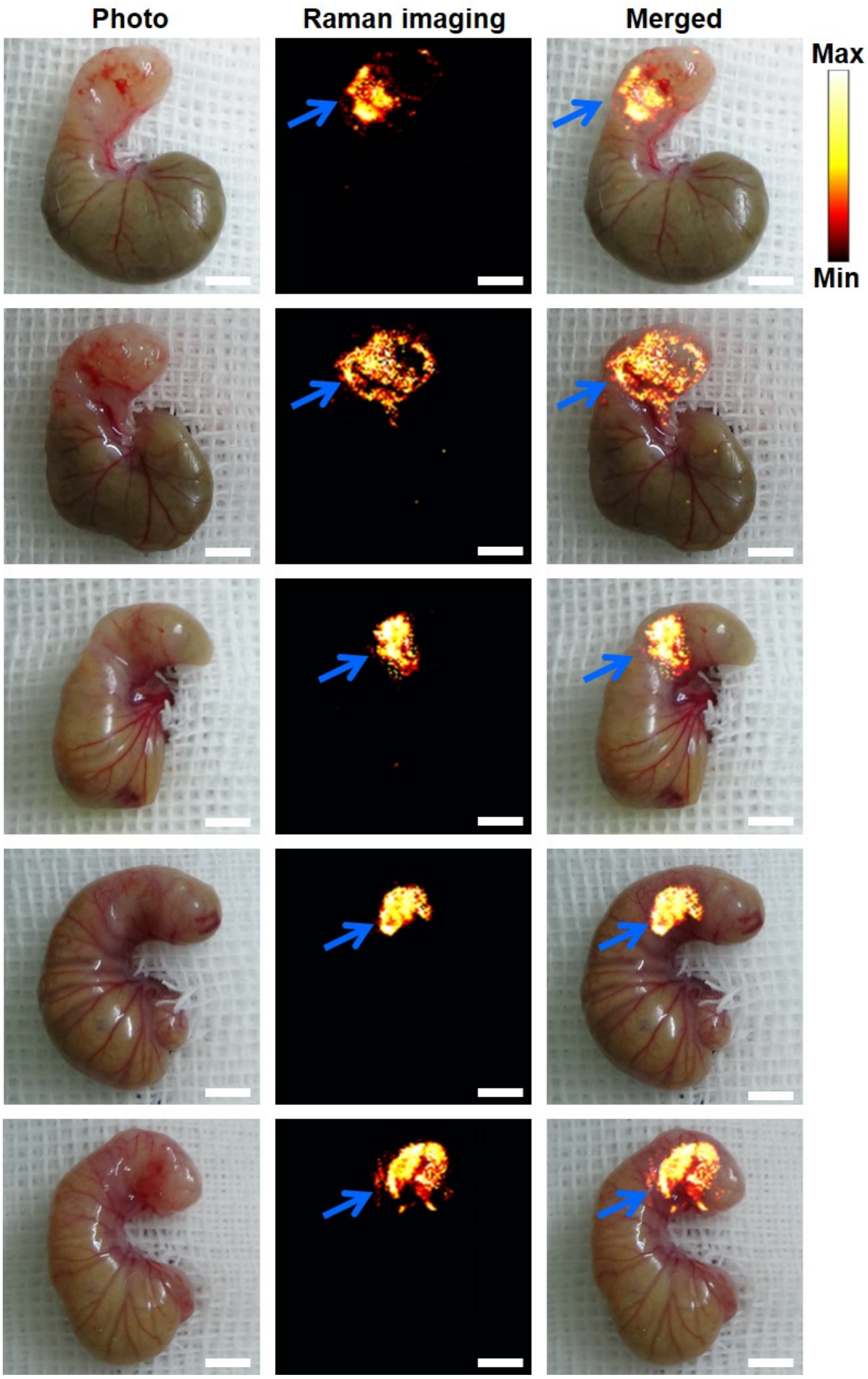

Supplementary Fig. 23 | Intraoperative Raman imaging of orthotopic colon tumor by BBT NPs. Intraoperative Raman imaging (894 cm^{-1}) merged with photographs of CT26-Luc tumor of mice following i.v. injection of BBT NPs at a dose of 1 mg kg^{-1} of BBT in additionally 5 independently repeated observations. Arrows, tumor. Raman measurement was carried out with a 5 × objective, 830-nm laser excitation, a laser power density of 31.3 mW, acquisition time of 0.3 s and one time accumulation. Bars, 3 mm.

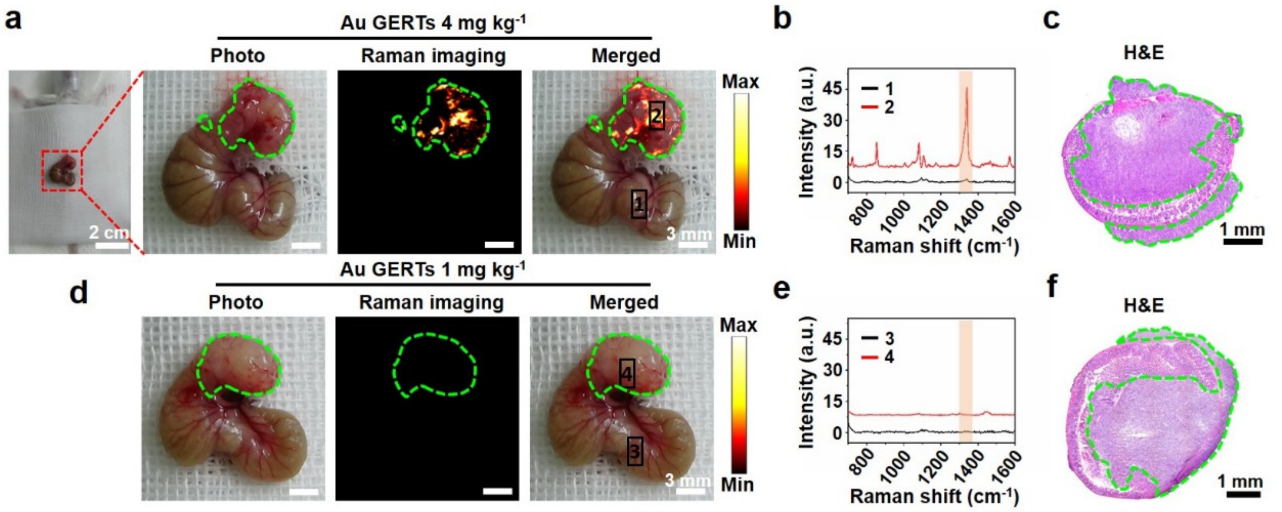

Supplementary Fig. 24 | Intraoperative Raman imaging of orthotopic mouse colon tumor by SERS. **a**, Live Raman imaging (1340 cm^{-1}) of orthotopic CT26-Luc tumor in mice following i.v. injected with NBT@Au GERTs at a dose of 4 mg kg^{-1} of Au. **b**, Raman spectra of sites 1 (cecum) and 2 (tumor) in (**a**), respectively. **c**, H&E staining of the tumor section following the imaging in (**a**). **d**, Raman imaging (1340 cm^{-1}) of the orthotopic CT26-Luc tumor in mice following i.v. injected with NBT@Au GERTs at a dose of 1 mg kg^{-1} of Au. **e**, Raman spectra of sites 3 (cecum) and 4 (tumor) in (**d**), respectively. **f**, H&E staining of the tumor section following the imaging in (**d**). Orange columns, peaks at 1340 cm^{-1}. Green circles, tumor region. Raman measurement was carried out with a 5 × objective, 830-nm laser excitation, a laser power of 31.3 mW, acquisition time of 0.3 s and one time accumulation. Image shown is representative of $n = 3$ independent replicates of experiments with similar results (**c**,**f**).

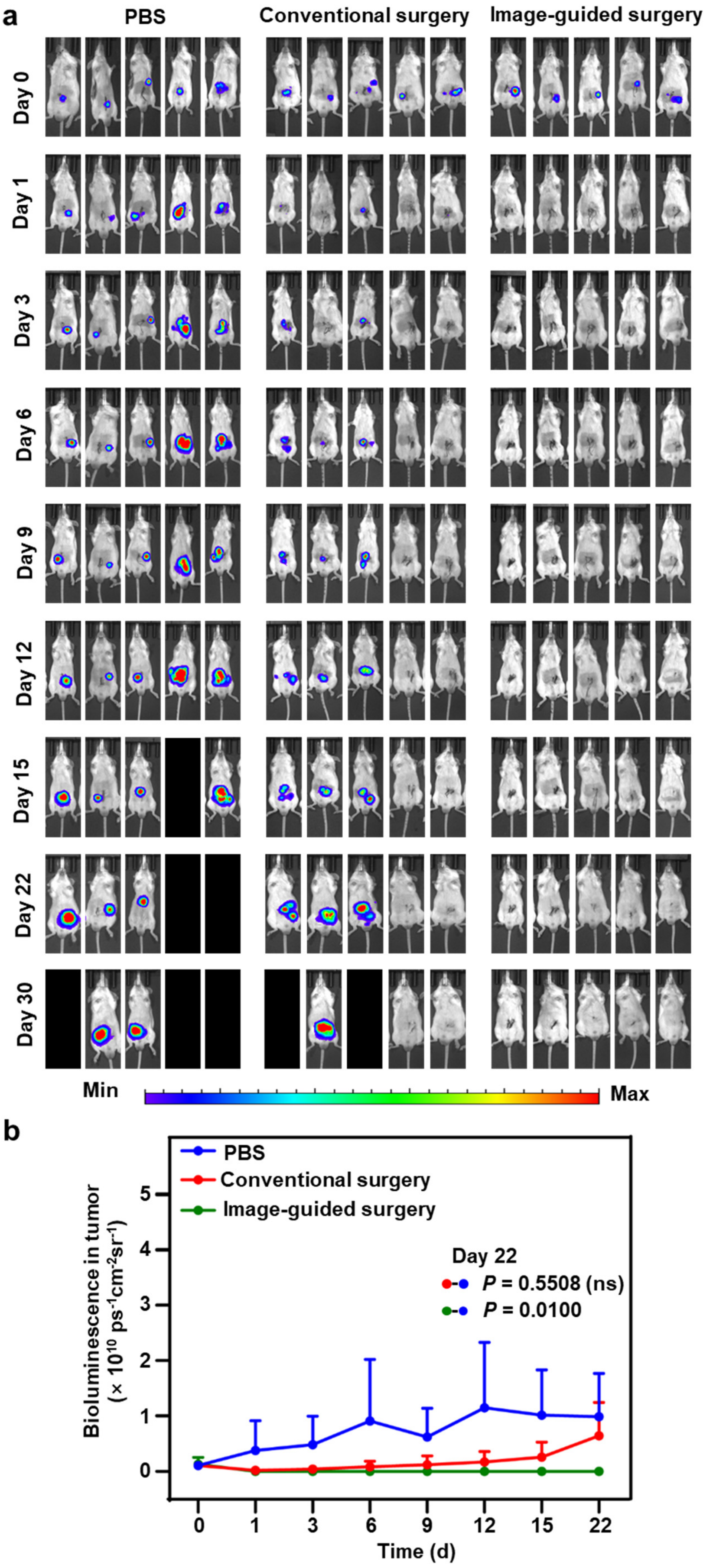
a
PBS
Conventional surgery
Image-guided surgery
Day 0
Day 1
Day 3
Day 6
Day 9
Day 12
Day 15
Day 22
Day 30
Min
Max
b
PBS
Conventional surgery
Image-guided surgery
Day 22
P = 0.5508 (ns)
P = 0.0100
Bioluminescence in tumor
(× 10^10 ps^-1cm^-2sr^-1)
Time (d)
0
1
2
3
4
5
0
1
3
6
9
12
15
22

Supplementary Fig. 25 | Tumor growth of mice following intraoperative SICTERS-based Raman image-guided surgery. a, Bioluminescence of the tumor-bearing mice after different treatments. Mice bearing orthotopic CT26-Luc tumor were divided into three treatment groups. PBS group, mice received PBS without the tumor resection. Conventional surgery group, mice received 40 mg kg^{-1} of BBT NPs, and the tumor resection by naked eyes after 24 h. Image-guided surgery group, mice received 40 mg kg^{-1} of BBT NPs, and the Raman image-guided tumor resection after 24 h. $n = 5$ biologically independent mice per group. **b**, Tumor growth curves of mice following different treatment in (**a**), measured by the bioluminescence. $n = 5$ biologically independent mice per group. Data are presented as the mean ± s.d.. Statistical significance was calculated via two-way ANOVA with Tukey's post hoc test, with *P* values indicated on the graphs. $P < 0.05$ was considered statistically significant. ns denotes no significant difference.

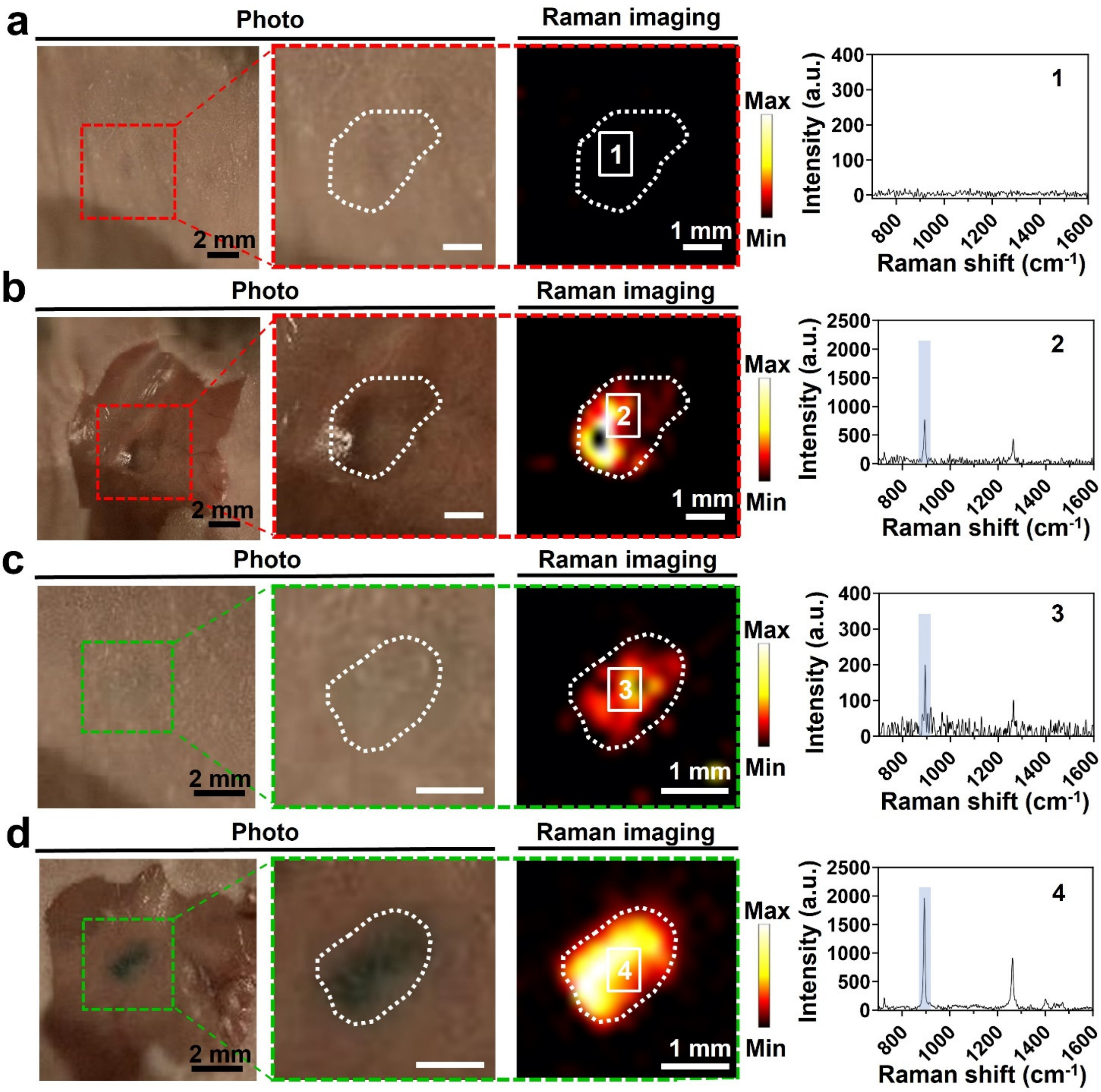

Supplementary Fig. 26 | *In vivo* Raman imaging of ALNs by BBT@Au NPs and BBT NPs. a,**b**, Photographs and Raman imaging (894 cm^{-1}) of ALNs after 15 min of the intradermal injection with the BBT@Au NPs (80 μg of Au). Raman imaging was performed before (**a**) and after (**b**) removing the skin, respectively. **c**,**d**, Photographs and Raman imaging (894 cm^{-1}) of ALNs after 15 min of the intradermal injection with the BBT NPs (80 μg of BBT). Raman imaging was performed before (**c**) and after (**d**) removing the skin, respectively. Raman spectra of sites 1, 2, 3 and 4 were showed in the right of each corresponding figure. White circles, ALNs. Blue columns, peaks at 894 cm^{-1} in the Raman spectra. Raman measurement was carried out with a 5 × objective, 830-nm laser excitation, a laser power of 62.6 mW, acquisition time of 0.3 s, and one time accumulation.

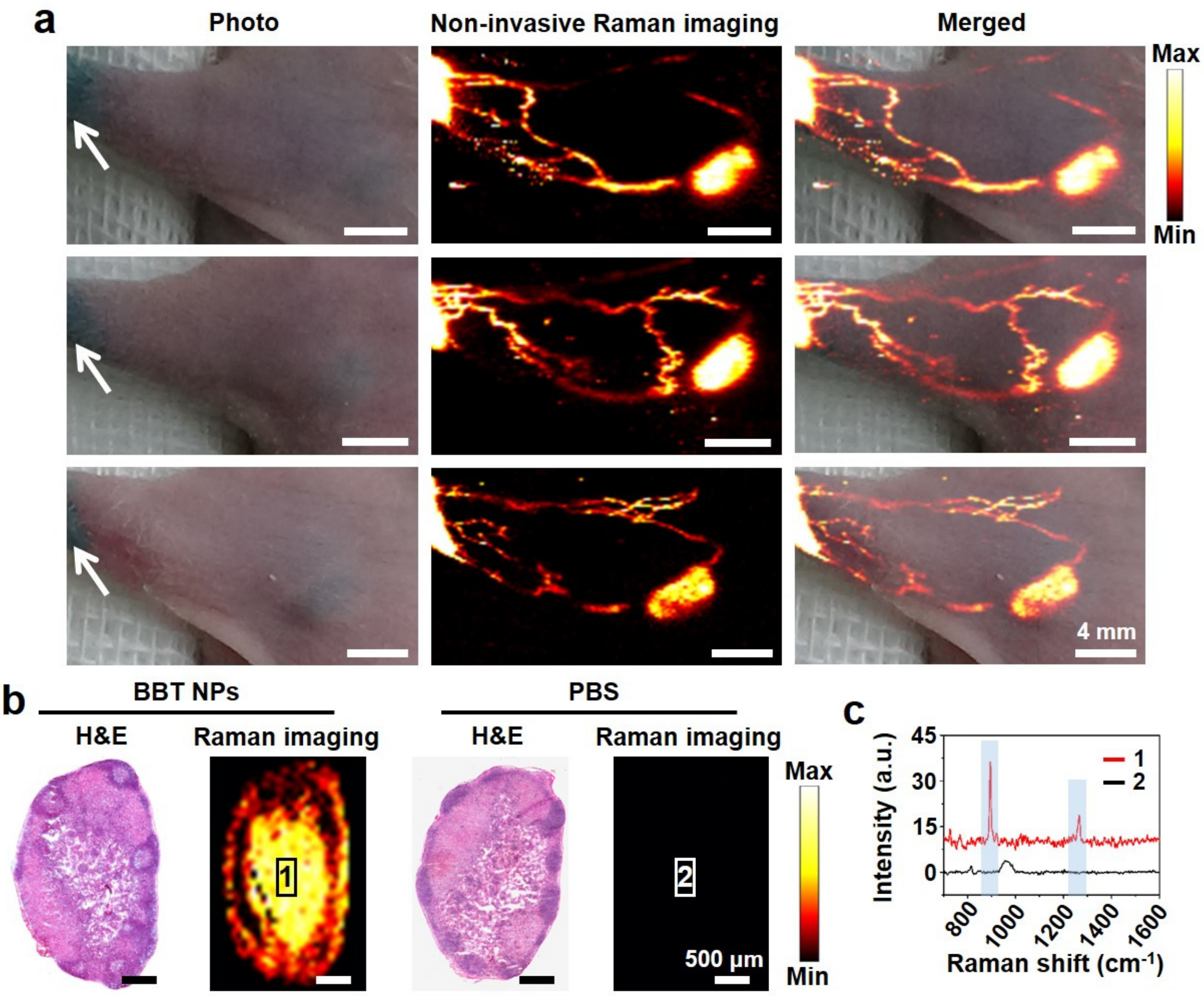

Supplementary Fig. 27 | *In vivo* Raman imaging of lymphatic drainage by SICTERS. a, Raman imaging (894 cm^{-1}) of lymphatic drainage of mice after 15 min of the intradermal injection with BBT NPs (150 μg of BBT) in the left front paw. n = 3 biologically independent mice. White arrows, the injection sites. **b,** Raman imaging (894 cm^{-1}, 50-μm thickness) and H&E staining (8-μm thickness) of the two adjacent cryo-sections of ALN samples from the BBT NPs-injected mice collected after the experiment in **Fig. 6f** or PBS control. Image shown is representative of n = 3 independent replicates of experiments with similar results. **c,** Raman spectra of sites in (**b**). Raman measurement was carried out with a 5 × objective, 830-nm laser excitation, a laser power of 62.6 mW in (**a**) or 6.3 mW in (**b**), acquisition time of 0.3 s, and one time accumulation.

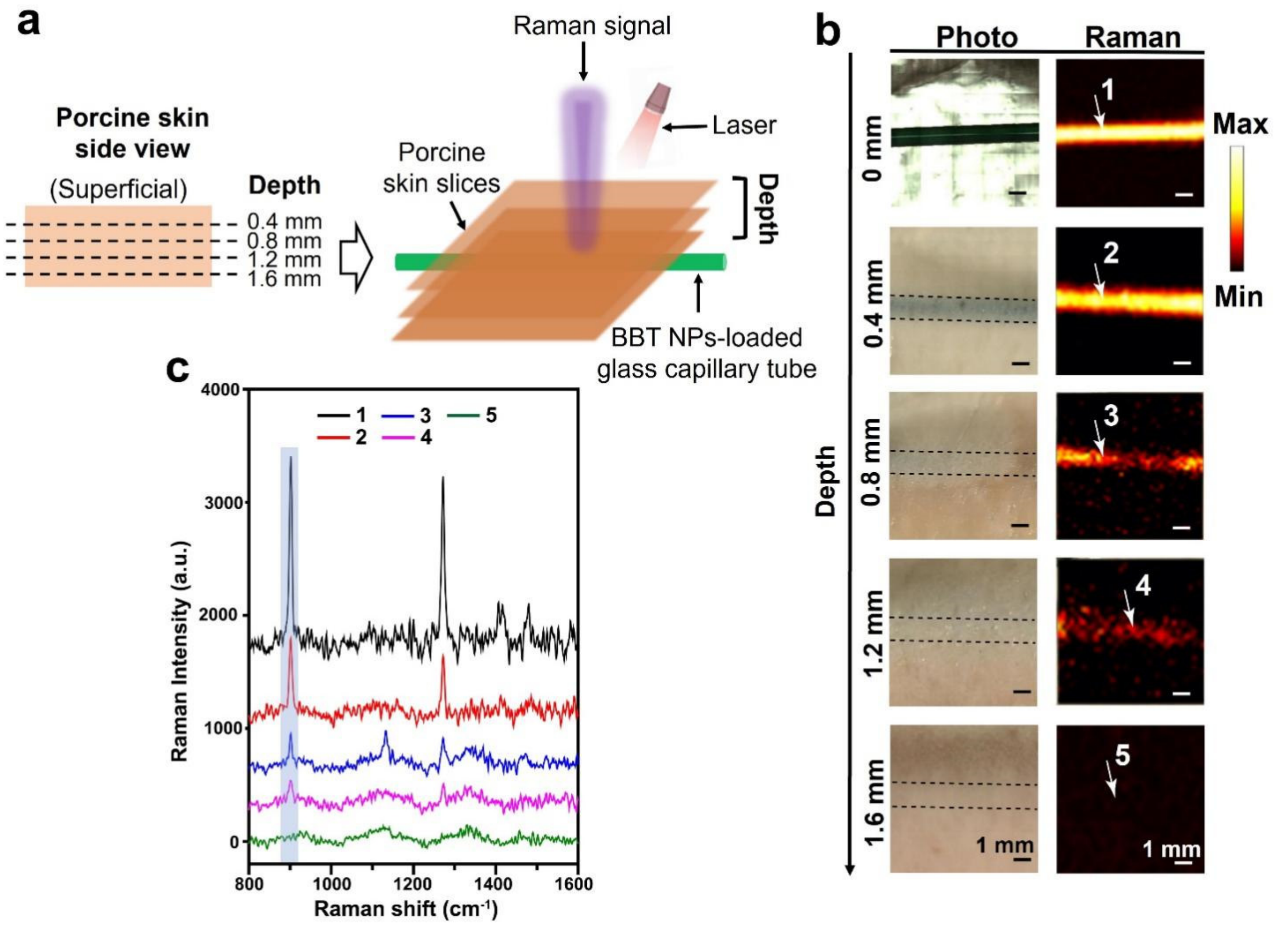

Supplementary Fig. 28 | *In vitro* depth analysis of SICTERS imaging by covering BBT NPs-loaded glass capillary tube with porcine skin slices. **a**, Illustration of the experimental setup. Porcine skin was sectioned into slices along the black dotted lines. The thickness of each slice of porcine skin was 0.4 mm. **b**, The Raman imaging of the BBT NPs (80 μg of BBT)-loaded glass capillary tube covered with or without porcine skin slices. **c**, Raman spectra of different regions in (**b**). Blue column, characteristic peaks of BBT NPs. Raman measurement was carried out with a 5 × objective, 830-nm laser excitation, a laser power of 62.6 mW, acquisition time of 1 s and one time accumulation.

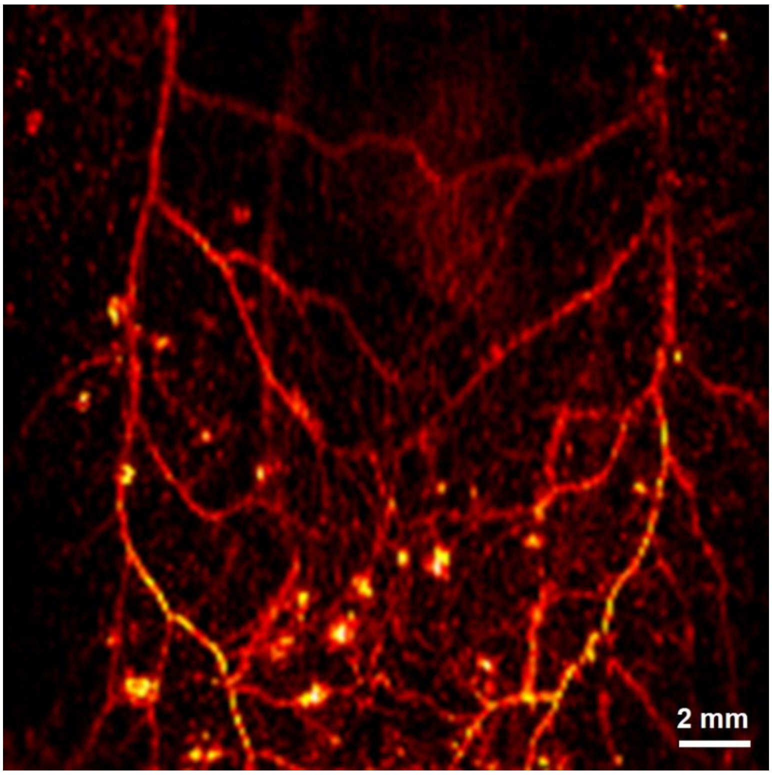

Supplementary Fig. 29 | The enlarged SICTERS image of Fig. 6h.

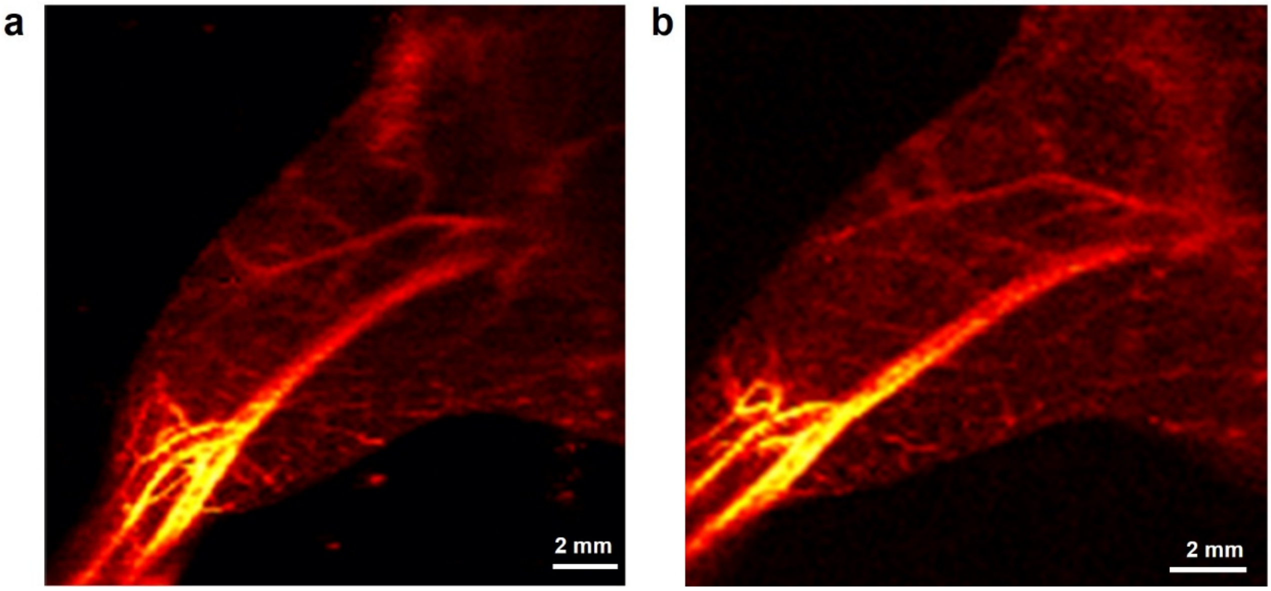

Supplementary Fig. 30 | *In vivo* Raman imaging (894 cm^{-1}) of blood vessels of mouse hindlimb following i.v. injection of BBT NPs. a, The enlarged SICTERS image of **Fig. 6l**. **b**, The SICTERS image of the repeated experiment in another mouse.

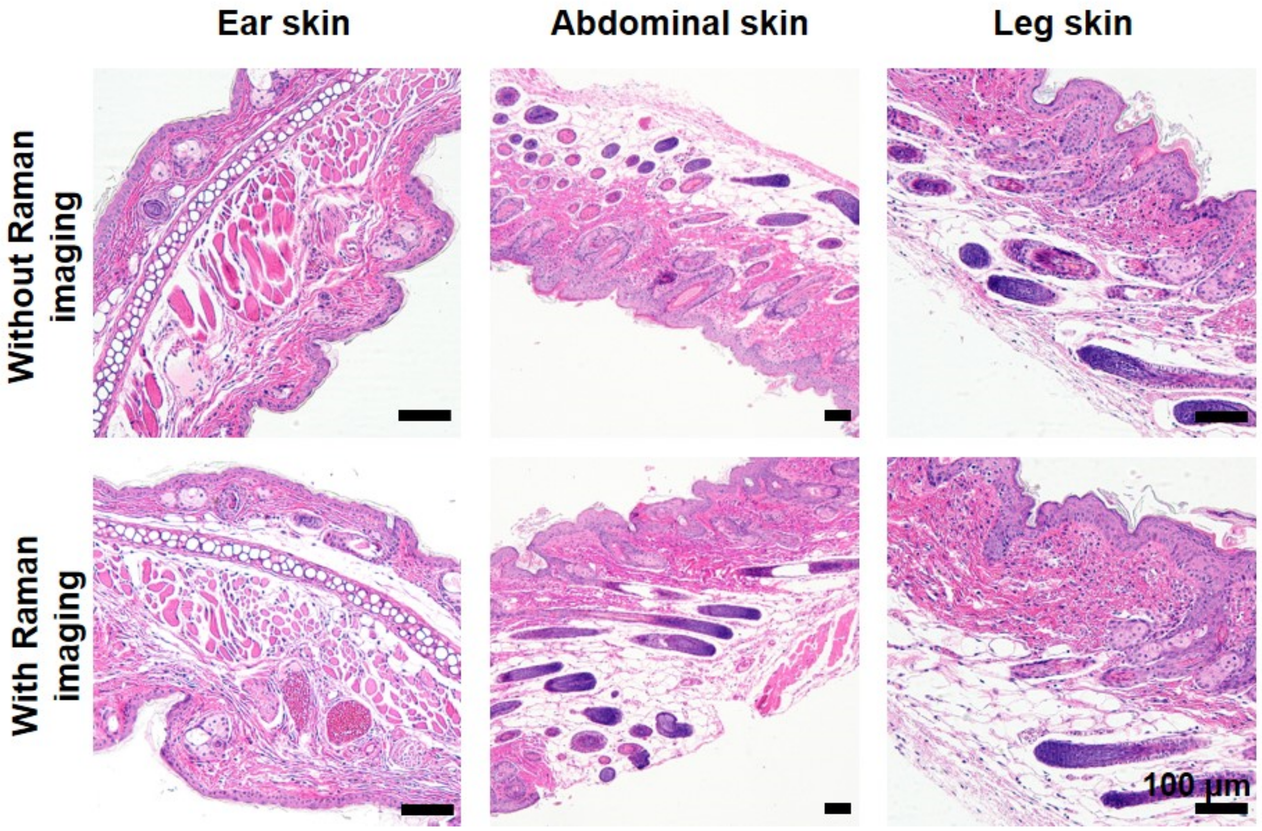

Supplementary Fig. 31 | Representative histological analysis of ear, abdominal and leg skin of mice with or without receiving Raman imaging by H&E staining. Raman measurement was carried out with a 5 × objective, 830-nm laser excitation, a laser power of 62.6 mW, acquisition time of 0.3 s and one time accumulation. For the group receiving Raman imaging, the samples were collected at 8 h post imaging. Image shown is representative of $n = 3$ independent replicates of experiments with similar results.

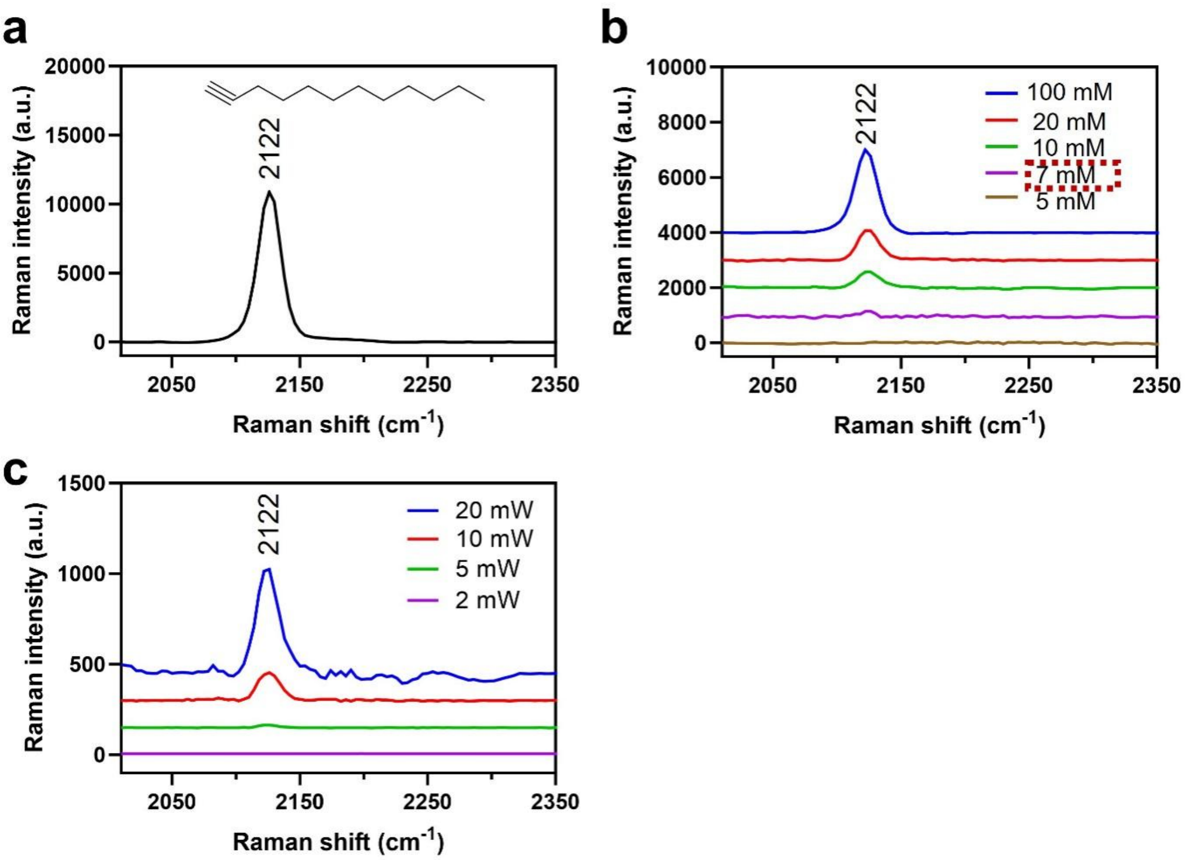

Supplementary Fig. 32 | Detection sensitivity measurement of stimulated Raman scattering (SRS)-based alkyne probes (1-dodecyne). a, Chemical structure and the Raman spectrum of 1-dodecyne measured with SRS instrument. **b,** Raman spectra of various concentrations of 1-dodecyne in dichloromethane. **c,** Raman spectra of 1-dodecyne in dichloromethane (10 mM) under laser radiation with different powers. A femtosecond laser with a fixed 1040 nm Stokes beam and a tunable 680 – 1300 nm pump beam was used. Raman measurement was carried out with a 10 × objective, a laser pump laser power of 20 mW and a laser Stokes laser power of 20 mW in (**a**) and (**b**). Raman measurement was carried out with a 10 × objective, pump laser power and Stokes laser power from 2 mW to 20 mW in (**c**).

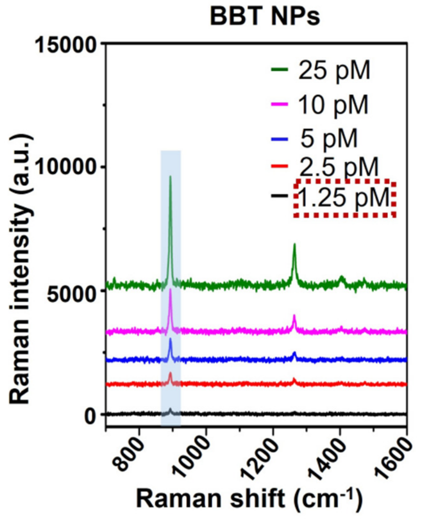

Supplementary Fig. 33 | Detection sensitivity measurement of SICTERS-based BBT NPs. Raman spectra of various concentrations of BBT NPs were measured. Blue column, peaks at 894 cm^{-1}. Raman measurement was carried out with an 830-nm laser excitation, a 5 × objective, a laser power of 62.6 mW, acquisition time of 1 s, and one time accumulation.

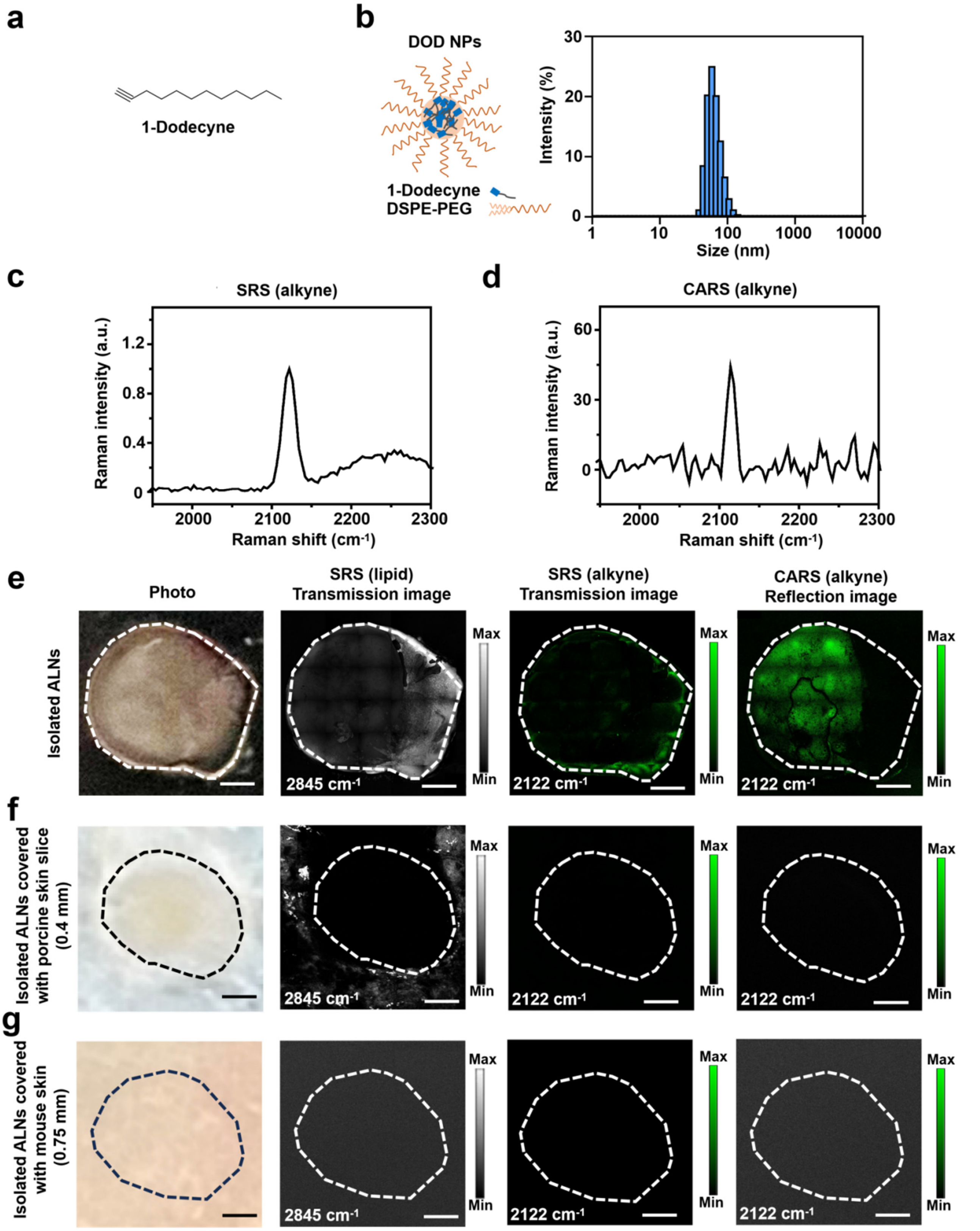

Supplementary Fig. 34 | Depth analysis of SRS or CARS imaging of mouse ALNs using alkyne-based probes. a, Chemical structure of 1-dodecyne. **b**, Schematic illustration of DOD NPs (left) and their size distribution (right). **c**, Raman spectrum of alkyne in DOD NPs (1.2 M of 1-dodecyne) with

SRS. **d**, Raman spectrum of alkyne in DOD NPs (1.2 M of 1-dodecyne) with CARS. Raman measurement was carried out with a 10 × objective, a laser pump laser power of 20 mW and a laser Stokes laser power of 20 mW in (**c**). Raman signal was collected by a 10 × objective and filtered by a 791 nm dichroic mirror, a 750 nm short pass filter, and a 710 nm bandpass filter in sequence in (**d**). **e-g**, The left front paw of nude mice was injected with DOD NPs (150 μg of 1-dodecyne) at 15 min before the imaging. The ALNs were isolated for bright-field (Photo) imaging, transmitted SRS imaging of the lipid signal (2845 cm^{-1}), and transmitted SRS and reflected CARS imaging of the alkyne signal (2122 cm^{-1}), respectively (**e**). The isolated ALNs were covered with a piece of porcine skin slice (~0.4 mm) followed by the different imaging (**f**). The isolated ALNs were covered with mouse skin (~0.75 mm) followed by the different imaging (**g**). Dotted circles, ALNs. Bars, 1 mm.

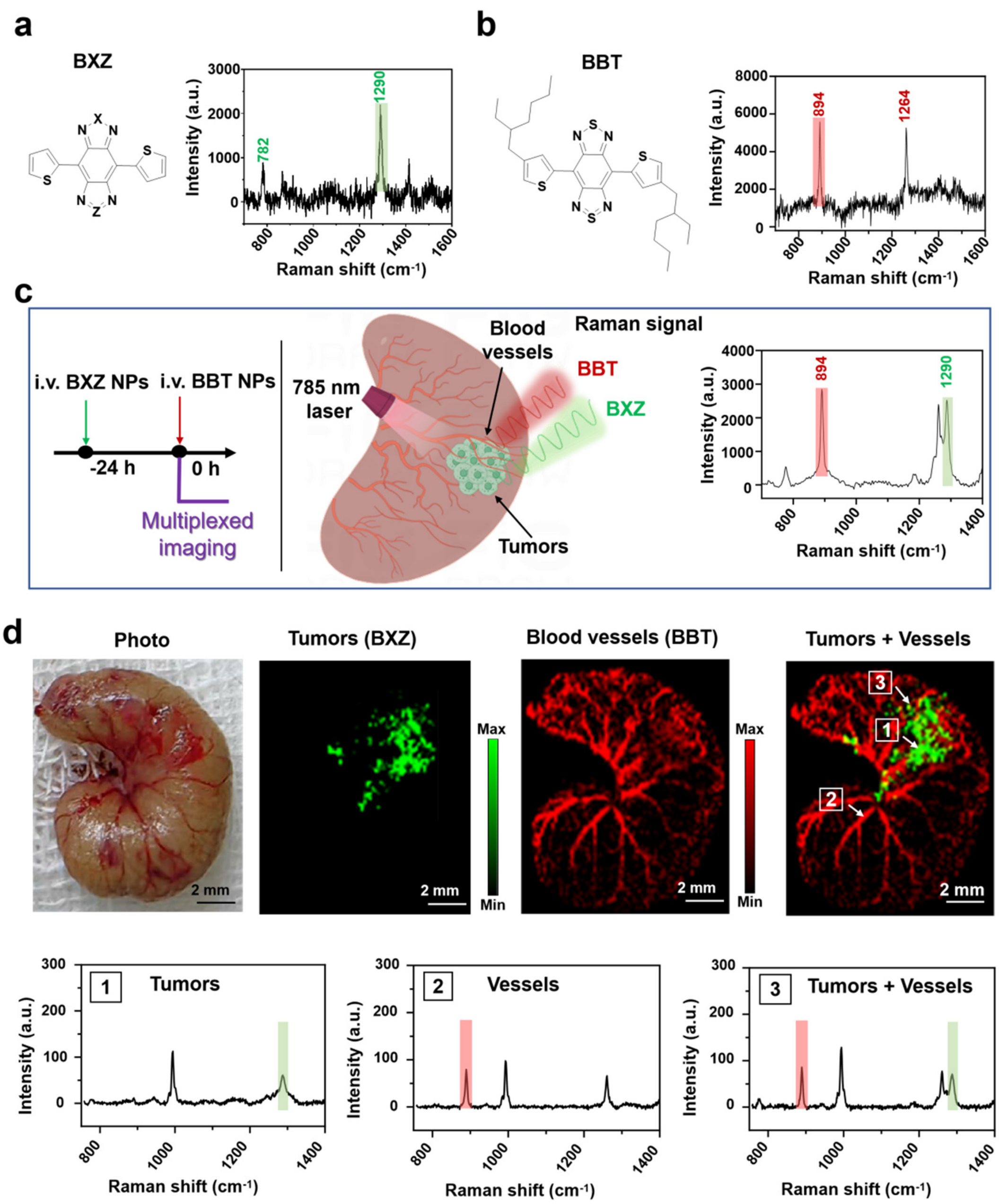

Supplementary Fig. 35 | Intraoperative multiplexed Raman imaging of tumors and blood vessels using SICTERS-based NPs. a, Chemical structure and Raman spectrum of BXZ (10 μM) in water/THF mixture (95:5, v/v), with characteristic peak at 1290 cm^{-1} (marked by green column) The X and Z represented elements or groups other than sulfur. **b**, Chemical structure and Raman spectrum of BBT (10 μM) in water/THF mixture (95:5, v/v), with characteristic peak at 894 cm^{-1} (marked by red column). **c**, Schematic illustration showing the dosage regimen and the intraoperative multiplexed Raman imaging protocol. **d**, Live Raman imaging of the cecum area with the CT26-Luc tumor of mice. Raman image of BXZ NPs (20 mg kg^{-1} of BXZ) with characteristic peak marked by the green column in the spectrum, representing the tumor region (green). The Raman image of BBT NPs (40 mg kg^{-1} of BBT) with characteristic peak marked by the red column in the spectrum, representing the blood vessels (red). Raman measurement was carried out with a 5 × objective, 785-nm laser excitation, laser power of 84.5 mW, one time accumulation, acquisition time of 1 s for (**a**) and (**b**), 0.15 s for (**d**), respectively.

Supplementary Tables

Supplementary Table 1 | Crystal data and structure refinement for mj23030_0m (BBT).

Identification code	mj23030_0m
Empirical formula	$C_{30}H_{38}N_4S_4$
Formula weight	582.88
Temperature/K	213.00
Crystal system	monoclinic
Space group	$P2_1/c$
a/Å	16.959(5)
b/Å	4.9659(13)
c/Å	17.889(4)
α/°	90
β/°	98.002(19)
γ/°	90
Volume/Å^3	1492.0(7)
Z	2
ρ_{calc}g/cm^3	1.297
μ/mm^{-1}	2.058
F(000)	620.0
Crystal size/mm^3	0.07 × 0.07 × 0.05
Radiation	GaKα (λ = 1.34139)
2Θ range for data collection/°	8.686 to 110.452
Index ranges	$-20 \leq h \leq 20$, $-4 \leq k \leq 6$, $-21 \leq l \leq 21$
Reflections collected	14049
Independent reflections	2778 [R_{int} = 0.0868, R_{sigma} = 0.0632]
Data/restraints/parameters	2778/7/174
Goodness-of-fit on F^2	1.069
Final R indexes [I>=2σ (I)]	R_1 = 0.1020, wR_2 = 0.2691
Final R indexes [all data]	R_1 = 0.1531, wR_2 = 0.3257
Largest diff. peak/hole / e Å^{-3}	0.58/-0.53

Supplementary Table 2 | The Raman scattering cross-sections for 4-NBT in various nanoparticles under the excitation at the different wavelengths.[a]

Reporter molecule	Raman scattering cross-section (cm^2 per molecule)	Number of reporter molecules per particle	Raman scattering cross-section (cm^2 per particle)
4-NBT	$(2.28 \pm 0.19) \times 10^{-28}$ (532 nm)<br>$(3.43 \pm 0.30) \times 10^{-29}$ (785 nm)<br>$(1.04 \pm 0.03) \times 10^{-29}$ (830 nm)		
4-NBT in NBT@Au NPs	$(3.01 \pm 0.51) \times 10^{-23}$ (532 nm)<br>$(3.09 \pm 0.34) \times 10^{-23}$ (785 nm)<br>$(1.06 \pm 0.09) \times 10^{-24}$ (830 nm)	2.39×10^4	$(7.20 \pm 1.21) \times 10^{-19}$ (532 nm)<br>$(7.39 \pm 0.82) \times 10^{-19}$ (785 nm)<br>$(2.52 \pm 0.21) \times 10^{-20}$ (830 nm)
4-NBT in NBT@Au GERTs	$(7.78 \pm 1.12) \times 10^{-23}$ (532 nm)<br>$(2.68 \pm 0.07) \times 10^{-21}$ (785 nm)<br>$(2.17 \pm 0.15) \times 10^{-21}$ (830 nm)	2.03×10^3	$(1.58 \pm 0.23) \times 10^{-19}$ (532 nm)<br>$(5.44 \pm 0.14) \times 10^{-18}$ (785 nm)<br>$(4.41 \pm 0.31) \times 10^{-18}$ (830 nm)
4-NBT in 4-NBT NPs	$(1.71 \pm 0.29) \times 10^{-28}$ (532 nm)<br>$(2.06 \pm 0.10) \times 10^{-29}$ (785 nm)<br>$(5.56 \pm 0.76) \times 10^{-30}$ (830 nm)		

[a]The cross-section values per molecule were calculated by measuring the Raman peak at 1340 cm^{-1} under the excitation at the wavelengths listed in the brackets. The largest cross-section values of each sample were selected for the comparative study in **Fig. 4d**. n =3 ~5 independent samples. Data are presented as the mean ± s.d..

Supplementary Table 3 | Hematologic analysis of mice after i.v. injection of BBT NPs.[a]

Item	Reference range	BBT NPs				
		1 d	7 d	14 d	30 d	60 d
WBC (10^9 L^{-1})	4.58-16.21	6.4 ± 0.4	5.3 ± 0.5	6.2 ± 1.8	4.8 ± 1.8	12.3 ± 0.9
LYMPH (10^9 L^{-1})	2.68-11.34	5.1 ± 0.3	4.0 ± 0.1	4.6 ± 1.3	3.8 ± 1.6	6.8 ± 1.4
LYMPH (%)	43.45-86.46	80.0 ± 1.7	75.7 ± 4.2	73.6 ± 1.3	77.3 ± 5.1	54.9 ± 7.7
NEUT (%)	7.30-41.77	16.7 ± 1.5	21.0 ± 3.7	22.6 ± 1.0	19.9 ± 4.8	37 ± 7.5
RBC (10^{12} L^{-1})	7.17-11.35	8.1 ± 0.6	7.9 ± 0.1	9.6 ± 0.4	9.8 ± 0.3	10.5 ± 0.6
HCT (%)	38.2-64.0	38.3 ± 1.7	38.1 ± 0.6	46.3 ± 2.0	46.1 ± 1.9	54.1 ± 1.8
MCV (fL)	47.5-66.7	47.3 ± 1.3	48.5 ± 0.4	48.4 ± 0.2	46.9 ± 0.4	51.5 ± 1.2
RDW (%)	14.7-19.1	18.0 ± 1.0	17.1 ± 0.2	17.0 ± 0.3	16.5 ± 0.9	15.2 ± 0.6
PLT (10^9 L^{-1})	469-2364	1094.3 ± 75.1	736.3 ± 61.5	1197.3 ± 93.3	1271.3 ± 253.3	996 ± 237.6
MPV (fL)	4.4-6.2	6.2 ± 0.4	5.9 ± 0.3	6.2 ± 0.3	6 ± 0.3	6.1 ± 0.2

[a] Blood samples were collected from ICR mice at 1, 7, 14, 30 and 60 d after *i.v.* injection of BBT NPs (40 mg kg^{-1}), respectively. Complete blood counts: blood levels of white blood cells (WBC), lymphocytes (LYMPH), neutrophils (NEUT), red blood cells (RBC), hematocrit (HCT), mean corpuscular volume (MCV), red cell volume distribution width (RDW), platelets (PLT), and mean platelet volume (MPV). Reference ranges of hematology data of healthy female ICR mice were obtained from Charles River Laboratories: (http://www.criver.com/). n = 3 biologically independent samples. Data are presented as mean ± s.d..

Supplementary Table 4 | Laser parameters used for different measurements in our SICTERS-based confocal Raman imaging system.

Laser wavelength	Objective	Laser spot area (cm^2)	Laser power (mW)	**Laser power density** (W cm^{-2})	Acquisition time (s)	Applica-tion
532 nm	5 ×	2.30×10^{-7}	0.79	3.43×10^3	10	*In vitro*
			15.8	6.87×10^4	1	*In vitro*
	20 ×	2.07×10^{-8}	1.60×10^{-2}	7.73×10^2	10	*In vitro*
			8.20×10^{-2}	3.96×10^3	10	*In vitro*
			8.45×10^{-5}	2.63×10^{-4}	10	*In vitro*
785 nm	**5 ×**	3.21×10^{-4}	84.5	2.63×10^2	1	*In vitro*
			**84.5**	**2.63×10^2**	**0.15**	***In vivo***
	20 ×	2.22×10^{-5}	8.20×10^{-2}	3.69	10	*In vitro*
			6.26×10^{-5}	2.10×10^{-4}	10	*In vitro*
			6.3	2.11×10^1	0.3	*In vitro*
830 nm	**5 ×**	2.98×10^{-4}	**31.3**	**1.05×10^2**	**0.3**	***In vivo***
			**62.6**	**2.10×10^2**	**0.3 or 1**	***In vivo***
			62.6	2.10×10^2	0.3 or 1	*In vitro*
	20 ×	2.17×10^{-5}	6.10×10^{-2}	2.81	0.2, 5 or 10	*In vitro*
	LW50 ×	2.49×10^{-6}	**22.0**	**8.84×10^3**	**0.3**	***In vivo***

References:

1 Qiu, Y. *et al.* Intraoperative detection and eradication of residual microtumors with gap-enhanced Raman tags. *ACS Nano* **12**, 7974-7985, doi:10.1021/acsnano.8b02681 (2018).

2 Zhang, Y. Q. *et al.* Ultraphotostable mesoporous silica-coated gap-enhanced Raman tags (GERTs) for high-speed bioimaging. *ACS Appl. Mater. Inter.* **9**, 3995-4005, doi:10.1021/acsami.6b15170 (2017).

3 Zhang, Y. *et al.* Ultrabright gap-enhanced Raman tags for high-speed bioimaging. *Nat. Commun.* **10**, 3905, doi:10.1038/s41467-019-11829-y (2019).
