# Supplementary material for: Self-stacked small molecules for ultrasensitive, substrate-free Raman imaging in vivo
Source: Nat Biotechnol. 2024 Aug 21;43(6):936–47. doi: 10.1038/s41587-024-02342-9 (PMC12167709; doi:10.1038/s41587-024-02342-9)
Supplement: Supplementary file 2 — Reporting Summary [file 41587_2024_2342_MOESM2_ESM.pdf]

nature portfolio

Corresponding author(s): Zeyu Xiao
Wei Lu
Xiaohong Fang

Last updated by author(s): Jun 20, 2024

Reporting Summary

Nature Portfolio wishes to improve the reproducibility of the work that we publish. This form provides structure for consistency and transparency in reporting. For further information on Nature Portfolio policies, see our Editorial Policies and the Editorial Policy Checklist.

Statistics

For all statistical analyses, confirm that the following items are present in the figure legend, table legend, main text, or Methods section.

n/a	Confirmed	
☐	☒	The exact sample size (*n*) for each experimental group/condition, given as a discrete number and unit of measurement
☐	☒	A statement on whether measurements were taken from distinct samples or whether the same sample was measured repeatedly
☐	☒	The statistical test(s) used AND whether they are one- or two-sided<br>*Only common tests should be described solely by name; describe more complex techniques in the Methods section.*
☒	☐	A description of all covariates tested
☒	☐	A description of any assumptions or corrections, such as tests of normality and adjustment for multiple comparisons
☐	☒	A full description of the statistical parameters including central tendency (e.g. means) or other basic estimates (e.g. regression coefficient) AND variation (e.g. standard deviation) or associated estimates of uncertainty (e.g. confidence intervals)
☐	☒	For null hypothesis testing, the test statistic (e.g. *F*, *t*, *r*) with confidence intervals, effect sizes, degrees of freedom and *P* value noted<br>*Give P values as exact values whenever suitable.*
☒	☐	For Bayesian analysis, information on the choice of priors and Markov chain Monte Carlo settings
☒	☐	For hierarchical and complex designs, identification of the appropriate level for tests and full reporting of outcomes
☒	☐	Estimates of effect sizes (e.g. Cohen's *d*, Pearson's *r*), indicating how they were calculated

Our web collection on statistics for biologists contains articles on many of the points above.

Software and code

Policy information about availability of computer code

Data collection	Data were collected using the software of the instrument described in each experiment.<br>Gaussian 09 software (DFT calculations)<br>SYBYL-6.9 (Molecular docking)<br>Zetasizer 7.12 software (Size measurement of particles)<br>ZetaView 8.04.02 SP2 software (Particle number measurement)<br>WiRE 4.3 software (Raman images)<br>Living Image 4.4 Software (bioluminescence animal imaging)
Data analysis	Graphpad Prism 10.0 software was used for statistical analyses.<br>Data Explorer (TM) 4.3 Software was used to analyze MALDI-TOF spectra.<br>MestReNova 6.1.0-6224 software was used to analyze Nuclear magnetic resonance spectra.<br>VEDA4 program was used to for vibrational assignments.<br>PKSolver 2.0 software was used to analyze pharmacokinetics.<br>MDI Jade 6 software was used to analyze XRD.<br>Mercury 3.8 software was used to analyze crystal structures.

For manuscripts utilizing custom algorithms or software that are central to the research but not yet described in published literature, software must be made available to editors and reviewers. We strongly encourage code deposition in a community repository (e.g. GitHub). See the Nature Portfolio guidelines for submitting code & software for further information.

Data

Policy information about availability of data

All manuscripts must include a data availability statement. This statement should provide the following information, where applicable:

- Accession codes, unique identifiers, or web links for publicly available datasets
- A description of any restrictions on data availability
- For clinical datasets or third party data, please ensure that the statement adheres to our policy

All data that support the findings of this study are available within the article and its Supplementary Information or from the corresponding author upon reasonable request. X-ray structure of HSA are publicly available on the PDB database (https://www.rcsb.org/structure/6WUW). Source data are provided with this paper.

Human research participants

Policy information about studies involving human research participants and Sex and Gender in Research.

Reporting on sex and gender	N/A
Population characteristics	N/A
Recruitment	N/A
Ethics oversight	N/A

Note that full information on the approval of the study protocol must also be provided in the manuscript.

Field-specific reporting

Please select the one below that is the best fit for your research. If you are not sure, read the appropriate sections before making your selection.

☒ Life sciences ☐ Behavioural & social sciences ☐ Ecological, evolutionary & environmental sciences

For a reference copy of the document with all sections, see nature.com/documents/nr-reporting-summary-flat.pdf

Life sciences study design

All studies must disclose on these points even when the disclosure is negative.

Sample size	Sample sizes were chosen based on previously published similar studies (Nat. Biotechnol. 2008,26, 83-90; Nat Mater. 2016, 15(2):235-42; Nat Biotechnol 2018, 36, 707-716). Sample sizes are clearly reported in the Figure Legends. In addition, we adhered to sample size requirements necessary (at least three samples) for determining statistical significance.
Data exclusions	No data were excluded from the analyses.
Replication	The in vitro, ex vivo, and in vivo imaging experiments were replicated independently for at least 3 times. In each group, $n \geq 3$ biological replicates was used.
Randomization	In all studies, samples were randomly allocated into experimental groups.
Blinding	The investigators were blinded to group allocation during data collection and analysis.

Reporting for specific materials, systems and methods

We require information from authors about some types of materials, experimental systems and methods used in many studies. Here, indicate whether each material, system or method listed is relevant to your study. If you are not sure if a list item applies to your research, read the appropriate section before selecting a response.

Materials & experimental systems

n/a	Involved in the study
☒	☐ Antibodies
☐	☒ Eukaryotic cell lines
☒	☐ Palaeontology and archaeology
☐	☒ Animals and other organisms
☒	☐ Clinical data
☒	☐ Dual use research of concern

Methods

n/a	Involved in the study
☒	☐ ChIP-seq
☒	☐ Flow cytometry
☒	☐ MRI-based neuroimaging

Eukaryotic cell lines

Policy information about cell lines and Sex and Gender in Research

Cell line source(s)	CT26-Luc from Imanis Life Sciences; NIH 3T3 from the American Type Culture Collection.
Authentication	None of the cell lines used were authenticated.
Mycoplasma contamination	All cell lines were tested negative for mycoplasma contamination.
Commonly misidentified lines (See ICLAC register)	None of commonly misidentified cell lines were used in this study.

Animals and other research organisms

Policy information about studies involving animals; ARRIVE guidelines recommended for reporting animal research, and Sex and Gender in Research

Laboratory animals	Mice were maintained in the animal facilities of Shanghai Jiao Tong University School of Medicine, under the following housing conditions: 12-hour light/ 12 hour dark cycle, 24 oC and 50% humidity. Crl:CD1(ICR) mice (female, 4-6 weeks), BALB/c mice (male, 6-8 weeks) and nude mice (male, 6-8 weeks) were purchased from Shanghai SLAC Laboratory Animal Co.,Ltd.
Wild animals	The study did not involve wild animals.
Reporting on sex	Crl:CD1(ICR) mice (female, 4-6 weeks), BALB/c mice (male, 6-8 weeks) and nude mice (male, 6-8 weeks) were used in the experiments. The major finding of this study is to develop a new technique for in vivo imaging. The applicability of the findings is not sex-specific; no sex-based analysis was performed.
Field-collected samples	The study did not involve samples collected from the field.
Ethics oversight	The Institutional Animal Care and Use Committee (IACUC) of Shanghai Jiao Tong University School of Medicine.

Note that full information on the approval of the study protocol must also be provided in the manuscript.

March 2021
