# Supplementary material for: Self-stacked small molecules for ultrasensitive, substrate-free Raman imaging in vivo
Source: Nat Biotechnol. 2024 Aug 21;43(6):936–47. doi: 10.1038/s41587-024-02342-9 (PMC12167709; doi:10.1038/s41587-024-02342-9)

checkCIF/PLATON report

Structure factors have been supplied for datablock(s) mj23030_0m

THIS REPORT IS FOR GUIDANCE ONLY. IF USED AS PART OF A REVIEW PROCEDURE FOR PUBLICATION, IT SHOULD NOT REPLACE THE EXPERTISE OF AN EXPERIENCED CRYSTALLOGRAPHIC REFEREE.

No syntax errors found. CIF dictionary Interpreting this report

Datablock: mj23030_0m

Bond precision:	C-C = 0.0066 A		Wavelength=1.34139
Cell:	a=16.959(5)	b=4.9659(13)	c=17.889(4)
	alpha=90	beta=98.002(19)	gamma=90
Temperature:	213 K		

	Calculated	Reported
Volume	1491.9(7)	1492.0(7)
Space group	P 21/c	P 1 21/c 1
Hall group	-P 2ybc	-P 2ybc
Moiety formula	C30 H38 N4 S4	C30 H38 N4 S4
Sum formula	C30 H38 N4 S4	C30 H38 N4 S4
Mr	582.88	582.88
Dx,g cm-3	1.298	1.297
Z	2	2
Mu (mm-1)	2.058	2.058
F000	620.0	620.0
F000'	623.27	
h,k,lmax	20,6,21	20,6,21
Nref	2859	2778
Tmin,Tmax	0.866,0.902	0.377,0.751
Tmin'	0.866	

Correction method= # Reported T Limits: Tmin=0.377 Tmax=0.751
AbsCorr = MULTI-SCAN

Data completeness= 0.972 Theta(max)= 55.226

R(reflections)= 0.1020(1446) wR2(reflections)= 0.3257(2778)

S = 1.069 Npar= 174

The following ALERTS were generated. Each ALERT has the format
test-name_ALERT_alert-type_alert-level.
Click on the hyperlinks for more details of the test.

Alert level C

```
PLAT029_ALERT_3_C _diffrn_measured_fraction_theta_full value Low .      0.978 Why?
PLAT084_ALERT_3_C High wR2 Value (i.e. > 0.25) ...................       0.33 Report
PLAT220_ALERT_2_C NonSolvent   Resd 1  C   Ueq(max)/Ueq(min) Range        3.3 Ratio
PLAT234_ALERT_4_C Large Hirshfeld Difference C9       --C10      .       0.21 Ang.
PLAT242_ALERT_2_C Low    'MainMol' Ueq as Compared to Neighbors of        C13 Check
PLAT260_ALERT_2_C Large Average Ueq of Residue Including        S1      0.128 Check
PLAT340_ALERT_3_C Low Bond Precision on  C-C Bonds ..............     0.0066 Ang.
PLAT906_ALERT_3_C Large K Value in the Analysis of Variance ......      7.307 Check
PLAT906_ALERT_3_C Large K Value in the Analysis of Variance ......      3.121 Check
PLAT911_ALERT_3_C Missing FCF Refl Between Thmin & STh/L=    0.600         59 Report
```

Alert level G

```
ABSMU01_ALERT_1_G  Calculation of _exptl_absorpt_correction_mu
                 not performed for this radiation type.
PLAT002_ALERT_2_G Number of Distance or Angle Restraints on AtSite          7 Note
PLAT003_ALERT_2_G Number of Uiso or Uij Restrained non-H Atoms ...          2 Report
PLAT072_ALERT_2_G SHELXL First  Parameter in WGHT  Unusually Large       0.20 Report
PLAT172_ALERT_4_G The CIF-Embedded .res File Contains DFIX Records          1 Report
PLAT177_ALERT_4_G The CIF-Embedded .res File Contains DELU Records          1 Report
PLAT192_ALERT_3_G A Non-default DELU Restraint Value for First Par     0.0050 Report
PLAT192_ALERT_3_G A Non-default DELU Restraint Value for SecondPar     0.0050 Report
PLAT333_ALERT_2_G Large Aver C6-Ring C-C Dist C1       -C3_a     .       1.42 Ang.
PLAT793_ALERT_4_G Model has Chirality at C9          (Centro SPGR)          R Verify
PLAT860_ALERT_3_G Number of Least-Squares Restraints .............          7 Note
PLAT910_ALERT_3_G Missing # of FCF Reflection(s) Below Theta(Min).          1 Note
PLAT912_ALERT_4_G Missing # of FCF Reflections Above STh/L=  0.600         22 Note
PLAT913_ALERT_3_G Missing # of Very Strong Reflections in FCF ....          1 Note
PLAT933_ALERT_2_G Number of HKL-OMIT Records in Embedded .res File         50 Note
PLAT978_ALERT_2_G Number C-C Bonds with Positive Residual Density.          0 Info
```

 0 **ALERT level A** = Most likely a serious problem - resolve or explain
 0 **ALERT level B** = A potentially serious problem, consider carefully
10 **ALERT level C** = Check. Ensure it is not caused by an omission or oversight
16 **ALERT level G** = General information/check it is not something unexpected

 1 ALERT type 1 CIF construction/syntax error, inconsistent or missing data
 9 ALERT type 2 Indicator that the structure model may be wrong or deficient
11 ALERT type 3 Indicator that the structure quality may be low
 5 ALERT type 4 Improvement, methodology, query or suggestion
 0 ALERT type 5 Informative message, check

It is advisable to attempt to resolve as many as possible of the alerts in all categories. Often the minor alerts point to easily fixed oversights, errors and omissions in your CIF or refinement strategy, so attention to these fine details can be worthwhile. In order to resolve some of the more serious problems it may be necessary to carry out additional measurements or structure refinements. However, the purpose of your study may justify the reported deviations and the more serious of these should normally be commented upon in the discussion or experimental section of a paper or in the "special_details" fields of the CIF. checkCIF was carefully designed to identify outliers and unusual parameters, but every test has its limitations and alerts that are not important in a particular case may appear. Conversely, the absence of alerts does not guarantee there are no aspects of the results needing attention. It is up to the individual to critically assess their own results and, if necessary, seek expert advice.

Publication of your CIF in IUCr journals

A basic structural check has been run on your CIF. These basic checks will be run on all CIFs submitted for publication in IUCr journals (*Acta Crystallographica*, *Journal of Applied Crystallography*, *Journal of Synchrotron Radiation*); however, if you intend to submit to *Acta Crystallographica Section C* or *E* or *IUCrData*, you should make sure that full publication checks are run on the final version of your CIF prior to submission.

Publication of your CIF in other journals

Please refer to the *Notes for Authors* of the relevant journal for any special instructions relating to CIF submission.

PLATON version of 28/11/2022; check.def file version of 28/11/2022

Datablock mj23030_0m - ellipsoid plot

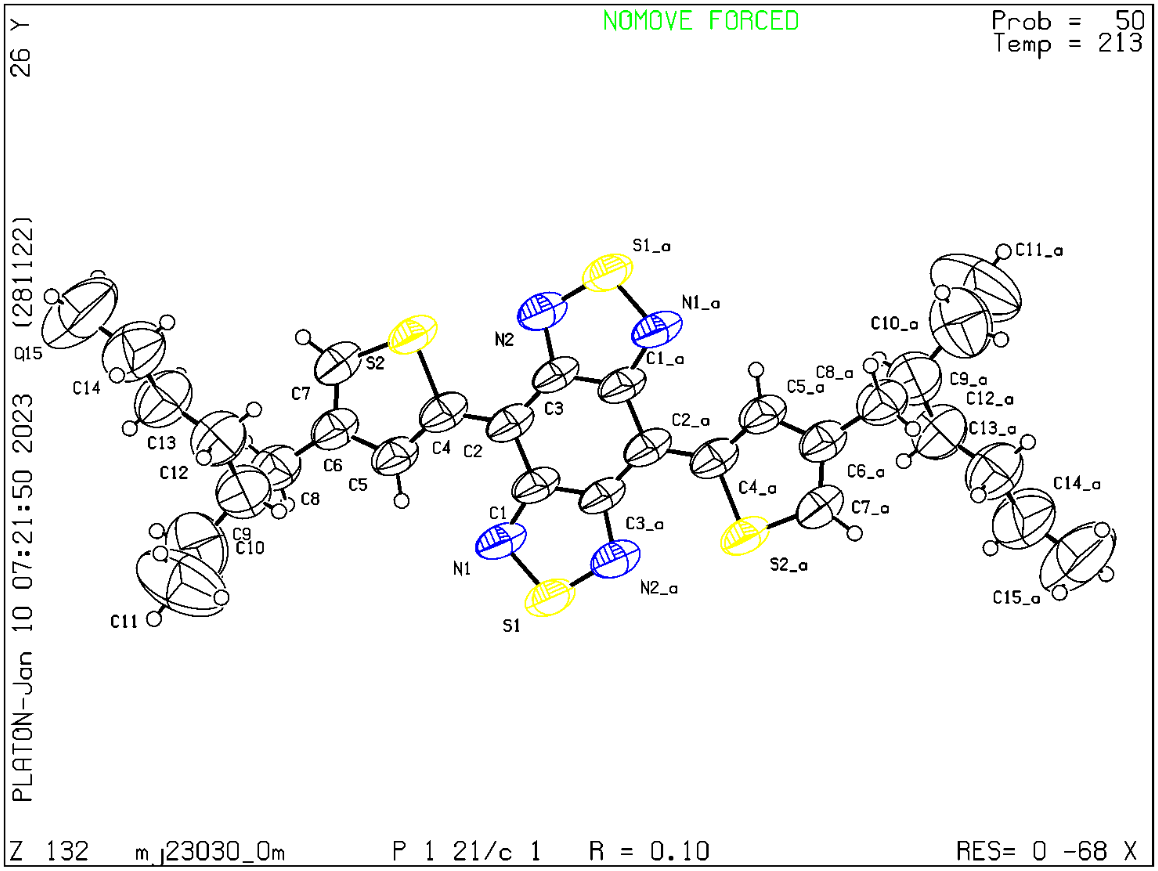

Supplement: Supplementary file 15 — CheckCIF file for BBT. [file 41587_2024_2342_MOESM15_ESM.pdf]
